# Stress-induced CD55 supports homologous recombination capacity and drives oxaliplatin resistance in colorectal cancer

**DOI:** 10.1186/s12964-026-03047-z

**Published:** 2026-07-10

**Authors:** Tianyang Wang, Xiaoyan Zhang, Xiaocan Yan, Guiying Wang, Lianmei Zhao

**Affiliations:** 1 Department of Hepatobiliary Surgery, The Second Hospital of Hebei Medical University, Shijiazhuang, 050000 China; 2 Department of Hematology, Key Laboratory of Hematology, The Second Hospital of Hebei Medical University, Shijiazhuang, 050000 China; 3 Department of Gastrointestinal Surgery, The Second Hospital of Hebei Medical University, Shijiazhuang, 050000 China; 4 Department of General Surgery, The Second Hospital of Hebei Medical University, Shijiazhuang, 050000 China; 5 Department of General Surgery, The Fourth Hospital of Hebei Medical University, Shijiazhuang, 050011 China; 6 Hebei Key Laboratory of Etiology Tracing and Individualized Diagnosis and Treatment for Digestive System Carcinoma, Shijiazhuang, 050000 China; 7 Research Center, The Fourth Hospital of Hebei Medical University, Shijiazhuang, 050011 China

**Keywords:** CD55, Colorectal cancer, Oxaliplatin resistance, Homologous recombination, DNA damage response, PARP inhibitors, PARPi-based combination sensitization, Stress signaling, Biomarker

## Abstract

**Background:**

Acquired resistance to oxaliplatin limits the efficacy of first-line chemotherapy in colorectal cancer (CRC), yet the stress-responsive signaling mechanisms that enable tumor cells to adapt to platinum-induced DNA damage remain incompletely understood. CD55, a glycosylphosphatidylinositol-anchored complement regulator, has recently been implicated in non-canonical cellular stress signaling beyond its classical immune-modulatory role. Here, we investigated whether stress-induced CD55 signaling contributes to oxaliplatin resistance by modulating DNA damage response and repair capacity.

**Methods:**

Oxaliplatin-resistant CRC cell lines were established through stepwise dose escalation. RNA sequencing with cross-validation against public datasets was used to identify candidate resistance genes. CD55 function was assessed using knockdown and overexpression approaches across seven CRC cell lines. Clinical correlations were evaluated using TCGA and GEO datasets. Homologous recombination (HR) capacity was measured using the DR-GFP reporter system, and DNA damage dynamics were assessed by γ-H2AX foci formation and comet assays. Drug interactions between oxaliplatin and PARP inhibitors were analyzed using the Chou–Talalay method. In vivo efficacy was evaluated in xenograft models.

**Results:**

CD55 was consistently upregulated in oxaliplatin-resistant CRC cells across three independent datasets and was induced by oxaliplatin treatment in a dose- and time-dependent manner, with stress-associated nuclear translocation observed following DNA damage. CD55 depletion sensitized all seven CRC cell lines to oxaliplatin (approximately 1.2- to 5.4-fold), with at least one siRNA reaching statistical significance in every line, irrespective of microsatellite instability or p53 status. In patient cohorts, elevated CD55 expression predicted poor response to FOLFOX therapy and independently correlated with reduced disease-free survival (hazard ratio = 3.47). Mechanistically, CD55 expression inversely correlated with genomic instability markers, and CD55 depletion significantly reduced HR efficiency in both parental and oxaliplatin-resistant CRC cells (~ 40–46% reduction), impaired BRCA1 induction, and prolonged γ-H2AX accumulation. In oxaliplatin-resistant cells, CD55 depletion converted the antagonistic oxaliplatin–olaparib interaction toward synergy in a dose-ratio–dependent manner (synergistic at 3:1 and 1:3, additive at 1:1; mean CI 0.58–0.95), reaching significance at the 3:1 ratio (*P* = 0.025). In xenograft models, CD55 depletion combined with dual-agent treatment reduced tumor burden by 62.8% in HCT116-OXR and consistently across all treatment arms in HT29-OXR.

**Conclusions:**

CD55 acts as a stress-responsive signaling regulator that partially supports homologous recombination capacity under genotoxic stress and promotes oxaliplatin resistance in CRC. These findings identify CD55 as both a predictive biomarker and a therapeutic vulnerability, and demonstrate that CD55 depletion can induce a partial HR impairment — operationally described as a “BRCAness-like” state — that is sufficient to sensitize HR-proficient tumors to PARP inhibition, thereby expanding the potential utility of PARP inhibitor–based combination strategies.

**Graphical Abstract:**

**Figure:**
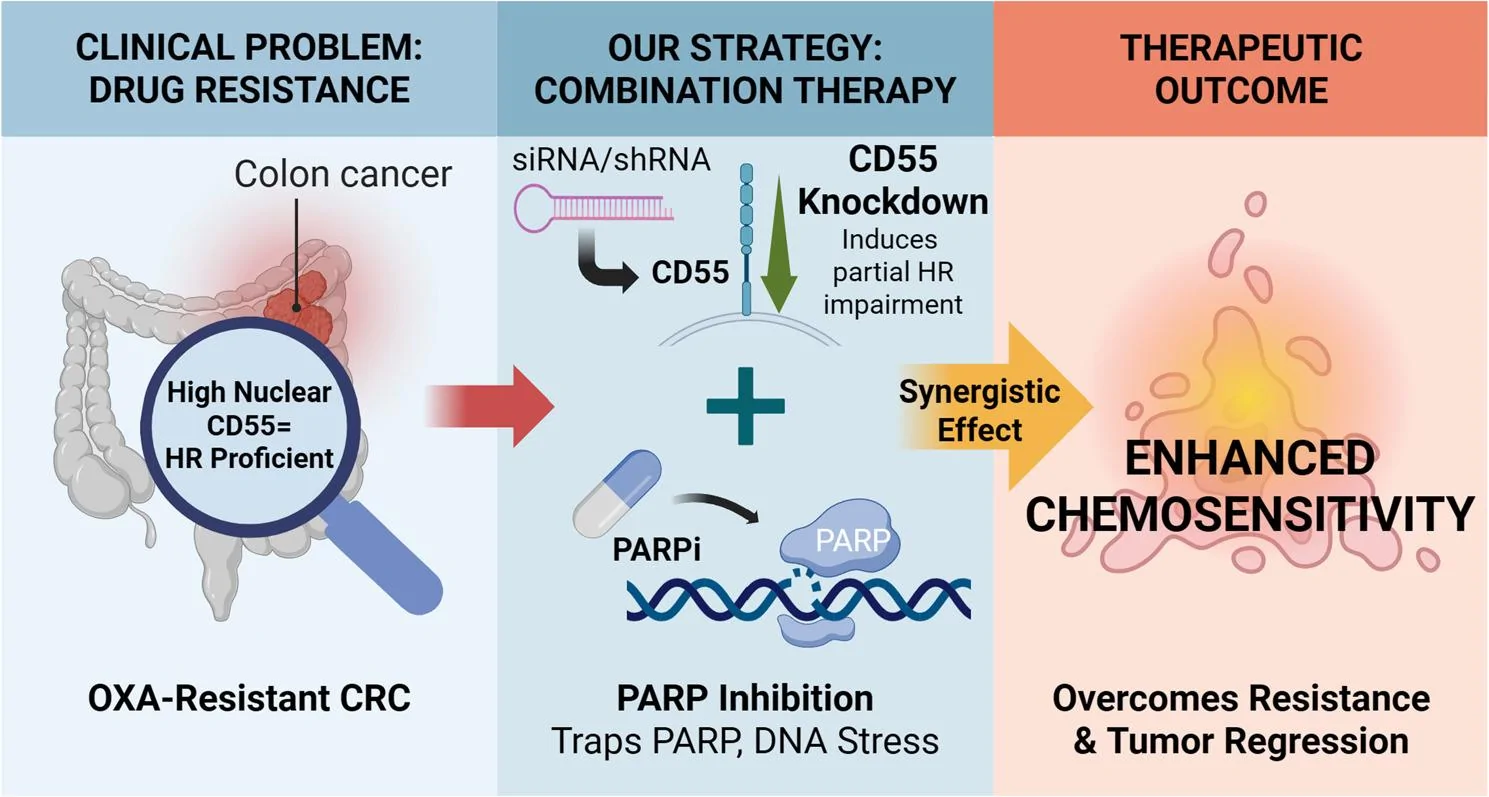

**Supplementary Information:**

The online version contains supplementary material available at https://doi.org/10.1186/s12964-026-03047-z.

## Introduction

Colorectal cancer (CRC) remains the third most commonly diagnosed malignancy worldwide, with over 1.9 million new cases and approximately 935,000 deaths reported annually [1, 2]. Oxaliplatin-based regimens, particularly FOLFOX (5-fluorouracil, leucovorin, and oxaliplatin), constitute the backbone of first-line treatment and have significantly improved patient outcomes [1]. However, the clinical benefit of oxaliplatin is frequently compromised by acquired resistance, wherein tumor cells adaptively rewire stress-response pathways to survive platinum-induced genotoxic insults [3, 4].

The cytotoxicity of oxaliplatin primarily derives from the formation of platinum-DNA adducts that impede transcription and replication, triggering replication stress and ultimately apoptosis [5–7]. To survive this genotoxic stress, tumor cells activate adaptive responses encompassing altered drug transport, enhanced detoxification, evasion of apoptosis, and critically, augmented DNA damage response (DDR) signaling [3, 6]. Among DDR pathways, homologous recombination (HR)-mediated repair of platinum-induced DNA lesions has emerged as a pivotal determinant of treatment sensitivity, as tumors with proficient HR can efficiently resolve cytotoxic DNA damage and evade cell death [3, 7, 8]. Tumors with HR deficiencies, exemplified by BRCA1/2-mutated breast and ovarian cancers, display exquisite sensitivity to platinum agents—a vulnerability that has been successfully exploited through synthetic lethality with poly(ADP-ribose) polymerase (PARP) inhibitors [4, 9, 10].

The success of PARP inhibitors in BRCA-mutant cancers has sparked considerable interest in extending this therapeutic strategy to HR-proficient tumors. However, PARP inhibitors have shown limited efficacy in colorectal cancer [11, 12], where germline BRCA mutations are rare (< 5%) [13, 14] and most tumors maintain proficient HR capacity [15]. This limitation has prompted the search for molecular targets whose inhibition could induce ‘functional BRCAness’—a state of acquired HR deficiency that renders otherwise resistant, HR-proficient tumors susceptible to PARP inhibitor-based synthetic lethality [16–18]. In this study, we use the term “BRCAness-like” in an operational sense to denote a partial HR impairment that is sufficient to confer PARP inhibitor sensitivity, rather than a complete BRCA1/2-deficient phenotype. Identifying druggable targets that regulate HR proficiency could therefore expand PARP inhibitor utility to a much broader patient population.

CD55, also known as decay-accelerating factor (DAF), is a glycosylphosphatidylinositol (GPI)-anchored glycoprotein that canonically functions to inhibit complement activation [19]. In cancer, CD55 is frequently overexpressed and has been associated with poor prognosis and enhanced metastatic potential across multiple malignancies, including colorectal cancer [20–22]. Intriguingly, emerging evidence reveals that CD55 participates in non-classical stress-responsive signaling beyond complement regulation, including roles in cellular adhesion, migration, and adaptation to microenvironmental stress [23, 24]. Notably, CD55 can undergo nuclear translocation under cellular stress and acquire non-canonical functions in modulating gene expression and chromatin regulation in ovarian cancer [25, 26]. However, whether CD55 functions as a stress-induced regulator of DNA damage response signaling to confer chemotherapy resistance in colorectal cancer remains unexplored.

In this study, we identify CD55 as a stress-induced signaling regulator that contributes to HR repair capacity under genotoxic stress and drives oxaliplatin resistance in CRC. We show that CD55 is consistently upregulated in oxaliplatin-resistant cells across multiple independent datasets and is induced by platinum treatment with concomitant nuclear translocation. Functional validation across seven genetically diverse cell lines demonstrates that CD55 depletion universally sensitizes CRC cells to oxaliplatin. Mechanistically, CD55 expression inversely correlates with genomic instability markers in patient tumors, and CD55 knockdown impairs HR efficiency and DNA damage resolution capacity. Therapeutically, CD55 depletion shifts antagonistic oxaliplatin–PARP inhibitor interactions toward synergy in a dose-ratio–dependent manner, creating a PARP inhibitor–sensitizing vulnerability in HR-proficient, oxaliplatin-resistant tumors. These findings establish CD55 as a stress-responsive coordinator of DNA repair signaling and a promising target for overcoming platinum resistance in colorectal cancer.

## Results

### Stress-adapted CRC cells acquire oxaliplatin resistance and display CD55 upregulation across independent datasets

**Fig. 1:**
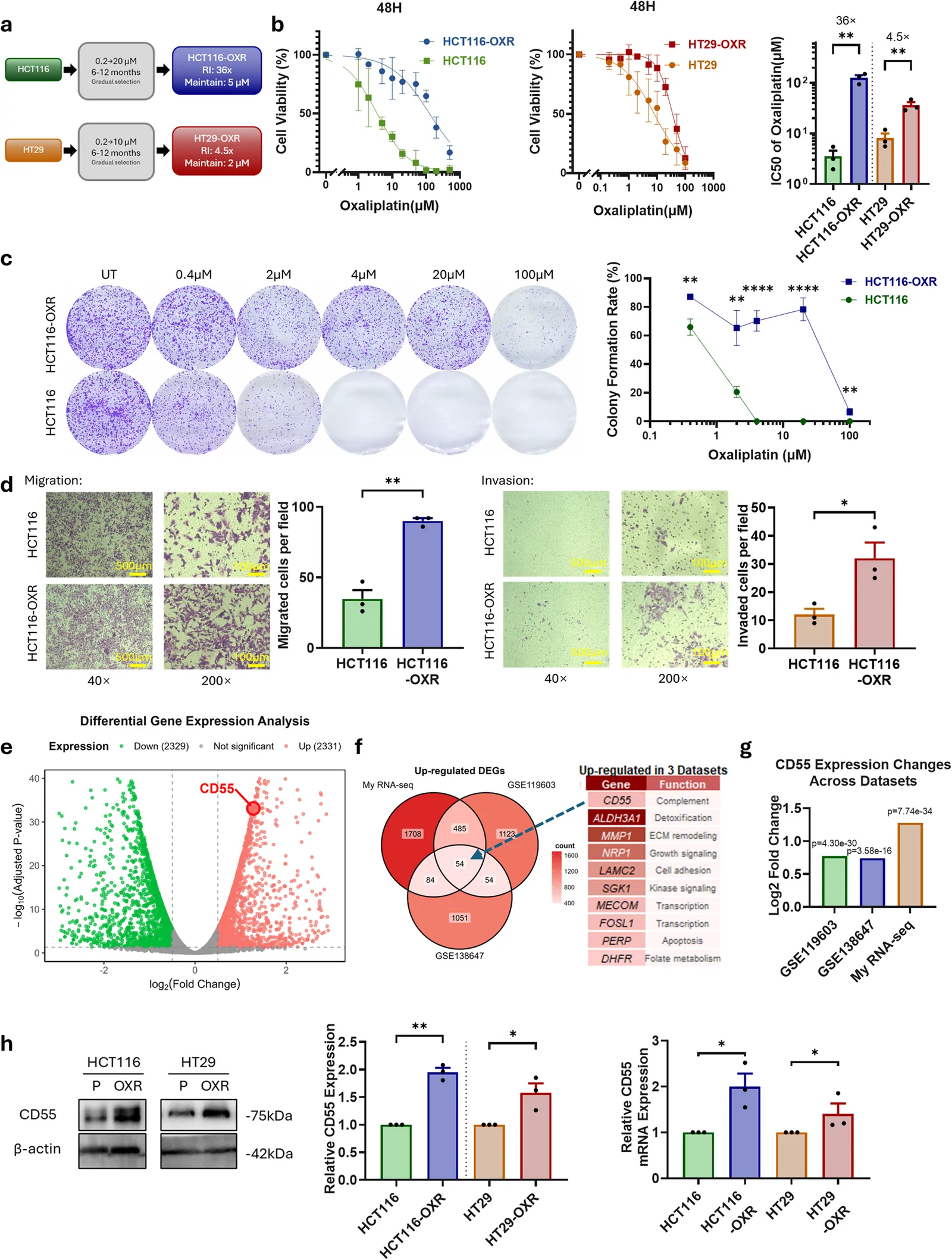
Stress-adapted CRC cells acquire oxaliplatin resistance and display CD55 upregulation across independent datasets. (**a**) Schematic workflow for generating oxaliplatin-resistant CRC cell lines through stepwise dose escalation. (**b**) CCK-8 viability assays showing absolute IC₅₀ values with individual biological replicates overlaid. HCT116-OXR: 125.2 ± 28.5 vs. 3.5 ± 1.8 µM (~ 36-fold); HT29-OXR: 36.0 ± 9.6 vs. 8.1 ± 3.3 µM (~ 4.5-fold). IC₅₀ are shown as arithmetic mean ± SEM, with statistical comparisons performed on log₁₀(IC₅₀) by two-tailed Welch’s t-test. (**c**) Colony formation assays under oxaliplatin treatment (0–4 µM). Representative images and quantification shown. (**d**) Transwell assays showing migration (90.0 ± 3.5 vs. 34.7 ± 11.0 cells/field) and invasion (29.7 ± 12.6 vs. 11.3 ± 3.3 cells/field). Scale bars: 500 μm (40×), 100 μm (200×). (**e**) Volcano plot from RNA-seq (*n* = 3/group; |log₂FC| > 0.5, FDR < 0.05). CD55 highlighted (log₂FC = 1.28). (**f**) Venn diagram showing 54 consistently upregulated and 179 downregulated genes across three datasets. Listed genes are grouped by function for display only; the complete quantitative ranking (by mean log₂FC and Fisher’s combined adjusted *p*-value) is provided in Supplementary Table S1. (**g**) CD55 expression across cohorts: log₂FC = 1.28 (this study), 0.77 (GSE119603), 0.74 (GSE138647). (**h**) Validation of CD55 upregulation at the protein (Western blot, left) and mRNA (qRT-PCR, right) levels in resistant versus parental cells. HCT116-OXR: 1.95-fold protein and 1.96-fold mRNA; HT29-OXR: 1.58-fold protein. For each assay, expression in resistant cells was normalized to its paired parental control, log₂-transformed, and tested against the null value of 0 (no change) by a two-tailed one-sample t-test (*n* = 3 independent experiments, df = 2). Protein: HCT116-OXR, *P* = 0.0039 (**); HT29-OXR, *P* = 0.047 (*). mRNA: HCT116-OXR, *P* = 0.041 (*); the HT29-OXR mRNA change was a non-significant trend (1.37-fold, *P* = 0.170, ns). Data are mean ± SEM. *P < 0.05, **P < 0.01; ns, not significant

To investigate acquired oxaliplatin resistance mechanisms, we established stable resistant derivatives of HCT116 (microsatellite-unstable) and HT29 (microsatellite-stable) colorectal cancer cell lines through 6-month stepwise dose escalation (Fig. 1a). The resulting HCT116-OXR and HT29-OXR cells displayed significantly elevated absolute IC₅₀ values compared to parental counterparts, with approximately 36-fold (125.2 ± 28.5 vs. 3.5 ± 1.8 µM) and 4.5-fold (36.0 ± 9.6 vs. 8.1 ± 3.3 µM) increases, respectively (Welch’s t-test on log₁₀IC₅₀, *P* < 0.01) (Fig. 1b, S1a). Resistant cells demonstrated enhanced clonogenic survival under oxaliplatin exposure (Fig. 1c, S1b) and exhibited increased migratory (90.0 ± 3.5 vs. 34.7 ± 11.0 cells/field, *P* < 0.01) and invasive capacities (29.7 ± 12.6 vs. 11.3 ± 3.3 cells/field, *P* < 0.05) (Fig. 1d).

To systematically identify molecular mediators of this resistant phenotype, we performed RNA-sequencing comparing resistant and parental HCT116 cells. Differential expression analysis identified CD55 among the significantly upregulated genes (log₂FC = 1.28, *P* < 0.001) (Fig. 1e). Importantly, cross-validation with two independent public datasets from oxaliplatin-resistant HCT116 models (GSE119603 and GSE138647) confirmed that CD55 upregulation is a conserved feature of oxaliplatin resistance: CD55 was significantly elevated in all three cohorts (log₂FC = 1.28, 0.77, and 0.74, respectively; all *P* < 0.001), placing it among only 54 genes showing concordant upregulation across datasets (Fig. 1f-g; full list and ranking in Supplementary Table S1). These 54 genes were enriched for cell-migration, extracellular-structure-organization and glycosaminoglycan-metabolism processes (Supplementary Fig. S1h), consistent with the enhanced migratory and invasive phenotype of resistant cells (Fig. 1d). Among the 54 concordant genes, CD55 was selected for functional validation based on biological plausibility rather than magnitude alone, given its non-canonical, stress-induced nuclear function in platinum resistance [26]. Western blotting confirmed CD55 protein upregulation in both HCT116-OXR (1.95-fold) and HT29-OXR (1.58-fold) cells, with concordant mRNA upregulation in HCT116-OXR (1.96-fold) and enhanced immunofluorescence intensity (1.86-fold) (Fig. 1h, S1c). These findings suggest that CD55 upregulation represents an adaptive response to chronic platinum exposure, prompting further investigation of its functional role in chemoresistance.

### Disrupting CD55 signaling sensitizes genetically diverse CRC cells to oxaliplatin

**Fig. 2:**
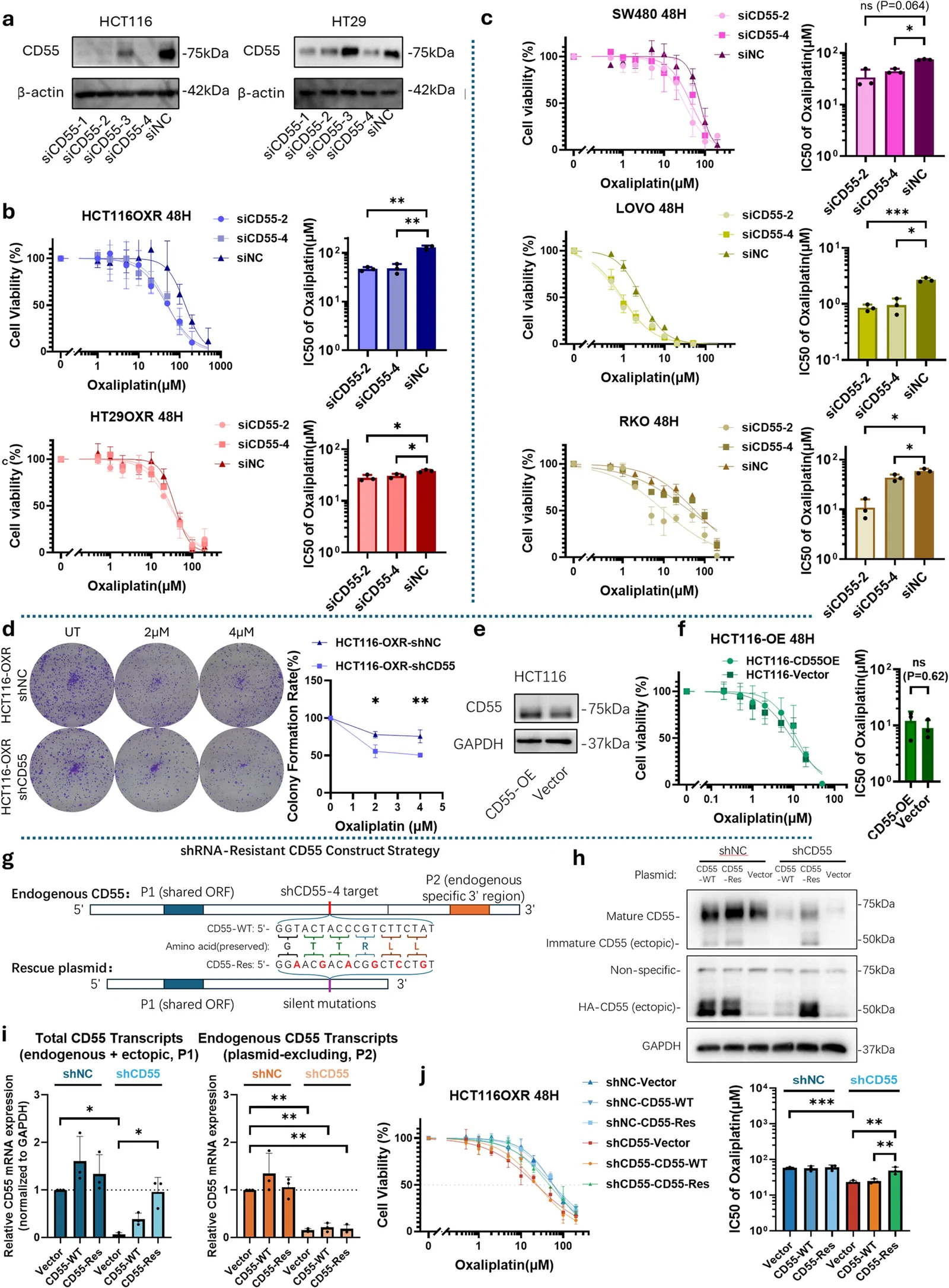
Disrupting CD55 signaling sensitizes genetically diverse CRC cells to oxaliplatin.(**a**) Western blot validation of siRNA knockdown efficiency in HCT116 and HT29 cells. Among four siRNAs, siCD55-4 achieved optimal knockdown (18.9% residual protein in HCT116, 30.7% in HT29; *P* < 0.001). β-actin, loading control. (**b**) Absolute IC₅₀ changes in resistant cells following CD55 knockdown. HCT116-OXR: 128.5 ± 12.5 µM (siNC) to 46.6 ± 4.5 µM (siCD55-2; 2.8-fold) and 48.0 ± 11.1 µM (siCD55-4; 2.7-fold); HT29-OXR: 38.0 ± 2.0 to 28.0 ± 3.8 µM (siCD55-2) and 30.5 ± 2.9 µM (siCD55-4; 1.2–1.4-fold). (**c**) Absolute IC₅₀ changes in SW480 (74.1 ± 1.8 to 33.4 ± 14.2 / 44.4 ± 4.9 µM), LOVO (2.7 ± 0.2 to 0.85 ± 0.12 / 0.95 ± 0.30 µM), and RKO (59.1 ± 6.4 to 10.9 ± 5.1 / 43.3 ± 6.6 µM). (**d**) Colony formation assays with stable shCD55 in HCT116-OXR cells under oxaliplatin treatment (10–14 days). (**e**) Western blot confirming CD55 overexpression (2.0-fold) in HCT116 cells. (**f**) Effect of CD55 overexpression on oxaliplatin sensitivity (IC₅₀: 9.1 ± 3.5 µM vector vs. 12.0 ± 5.8 µM CD55-OE, *P* = 0.62, ns). (**g**) Schematic of the shRNA-resistant rescue strategy. Silent substitutions (underlined) within the shCD55-4 target site generate CD55-Res, which escapes shCD55 silencing while preserving the encoded amino acid sequence. CD55-WT and empty vector serve as controls. P1, primers within the shared ORF detecting total CD55 (endogenous + ectopic); P2, primers in an alternate 3′ region detecting endogenous CD55 only. (**h**) Western blot (48 h): anti-CD55 marks mature (~ 75 kDa) and precursor (~ 50 kDa) bands; HA quantified at ~ 50 kDa (non-specific anti-HA at ~ 75 kDa). CD55-Res, not CD55-WT, restored mature CD55 (~ 48%) and gave ~ 3-fold higher HA precursor (*P* < 0.001). (**i**) qRT-PCR (P1/P2): P2 confirmed equal endogenous knockdown across conditions; P1 showed restoration only with CD55-Res. (**j**) Oxaliplatin IC₅₀ (six conditions): CD55-Res restored resistance (29.3 → 85.9 µM, *P* = 0.0012); CD55-WT did not (40.0 µM, ns).Data: mean ± SEM, *n* = 3 independent biological replicates; IC₅₀ compared on log₁₀(IC₅₀) by Welch’s unpaired two-tailed t-test (b, c, f) and rescue panels (h–j) by one-way ANOVA with Tukey’s HSD. *P < 0.05, **P < 0.01, ***P < 0.001; ns, not significant

To determine whether CD55 functionally contributes to chemoresistance, we established both transient (siRNA) and stable (shRNA) knockdown models. Among four siRNAs tested, siCD55-4 achieved optimal knockdown efficiency, reducing CD55 protein to 18.9% in HCT116 and 30.7% in HT29 cells (*P* < 0.001) (Fig. 2a, S2a). Stable knockdown lines demonstrated 60–74% protein reduction across multiple cell lines (Figure S2b).

CD55 depletion with two independent siRNAs sensitized all seven cell lines to oxaliplatin, with at least one siRNA reaching statistical significance in every line. In resistant models, HCT116-OXR IC₅₀ decreased from 128.5 ± 12.5 µM (siNC) to 46.6 ± 4.5 µM (siCD55-2; 2.8-fold, *P* < 0.001) and 48.0 ± 11.1 µM (siCD55-4; 2.7-fold, *P* < 0.01), while HT29-OXR showed 1.2- to 1.4-fold sensitization (38.0 ± 2.0 to 28.0 ± 3.8 and 30.5 ± 2.9 µM; both *P* < 0.05) (Fig. 2b, S2c). In parental cells, HCT116 IC₅₀ decreased approximately 4.0- to 4.3-fold (7.3 ± 3.0 to 1.8 ± 1.0 µM, *P* < 0.05, siCD55-2; and to 1.7 ± 1.3 µM for siCD55-4, *P* = 0.060), and HT29 showed 1.4- to 1.7-fold reduction (29.5 ± 5.2 to 17.2 ± 3.5 µM, *P* < 0.05 for siCD55-4) (Figure S2d). The sensitization extended to three additional lines: SW480 (1.7- to 2.2-fold; 74.1 ± 1.8 to 44.4 ± 4.9 µM, *P* < 0.05 for siCD55-4), LOVO (2.8- to 3.2-fold; 2.7 ± 0.2 to 0.85 ± 0.12 µM, *P* < 0.001), and RKO (1.4- to 5.4-fold; 59.1 ± 6.4 to 10.9 ± 5.1 µM, *P* < 0.05) (Fig. 2c, S2e). The magnitude of sensitization varied by genetic context, but the direction was consistent across all seven lines. Colony formation assays confirmed these findings, with stable CD55 knockdown reducing colony formation by 20–25% under oxaliplatin treatment (*P* < 0.05) (Fig. 2d).

CD55 overexpression showed a trend toward increased resistance (IC₅₀: 9.1 ± 3.5 µM vector vs. 12.0 ± 5.8 µM CD55-OE), though this did not reach statistical significance (*P* = 0.62) (Fig. 2e-f, S2f). To investigate the mechanism of sensitization, we examined apoptotic responses.

Flow cytometry revealed that CD55 knockdown significantly enhanced oxaliplatin-induced apoptosis, increasing total apoptotic rates from 33.4% to 47.3% in HCT116 cells and from 17.4% to 26.6% in HCT116-OXR cells. Late apoptosis was particularly elevated in resistant cells with CD55 knockdown (19.7% vs. 9.2%, *P* < 0.001) (Figure S2g). Molecularly, CD55 depletion reduced BCL-2 expression while increasing BAX levels, resulting in a decreased BCL-2/BAX ratio (*P* < 0.01) (Figure S2h). CD55 knockdown also reduced invasive capacity by 50.5% in HCT116 cells and 73.3% in HCT116-OXR cells (Figure S2i).

To exclude off-target effects of the CD55 shRNA, we generated an shRNA-resistant CD55 construct (CD55-Res) bearing silent substitutions within the shCD55-4 target site, and compared it to empty vector and wild-type CD55 (CD55-WT), which differs from CD55-Res only by these silent substitutions (Fig. 2g). In HCT116-OXR cells stably expressing shCD55, a primer pair specific to endogenous CD55 transcripts (P2) confirmed equivalent shRNA-mediated knockdown across all plasmid conditions, whereas a primer pair detecting total CD55 (P1) showed transcript restoration only with CD55-Res (Fig. 2i). At the protein level, CD55 was detected as a mature ~ 75 kDa glycoform and an under-glycosylated ~ 50 kDa precursor. CD55-Res, but not CD55-WT, restored mature CD55 in the shCD55 background (~ 48% of wild-type level, *P* < 0.01), and yielded a ~ 3-fold higher ectopic HA-CD55 precursor signal than CD55-WT (*P* < 0.001), directly demonstrating the sequence specificity of the shRNA (Fig. 2h, S2j). Functionally, CD55-Res significantly restored oxaliplatin IC₅₀ (29.3 → 85.9 µM, *P* = 0.0012; Fig. 2j, S2k), whereas CD55-WT did not rescue. The magnitude of IC₅₀ recovery (~ 46%) closely matched mature CD55 protein recovery (~ 48%), consistent with a direct dose–response relationship between mature CD55 abundance and oxaliplatin resistance, and indicating that incomplete rescue reflects partial restoration of mature CD55 protein rather than off-target effects. Rescue of homologous recombination repair by CD55-Res is described in subsequent sections. Together, these data establish that the observed phenotypes are specifically attributable to CD55 loss-of-function.

### High CD55 associates with poor chemotherapy response and an HR-proficient clinical phenotype

**Fig. 3:**
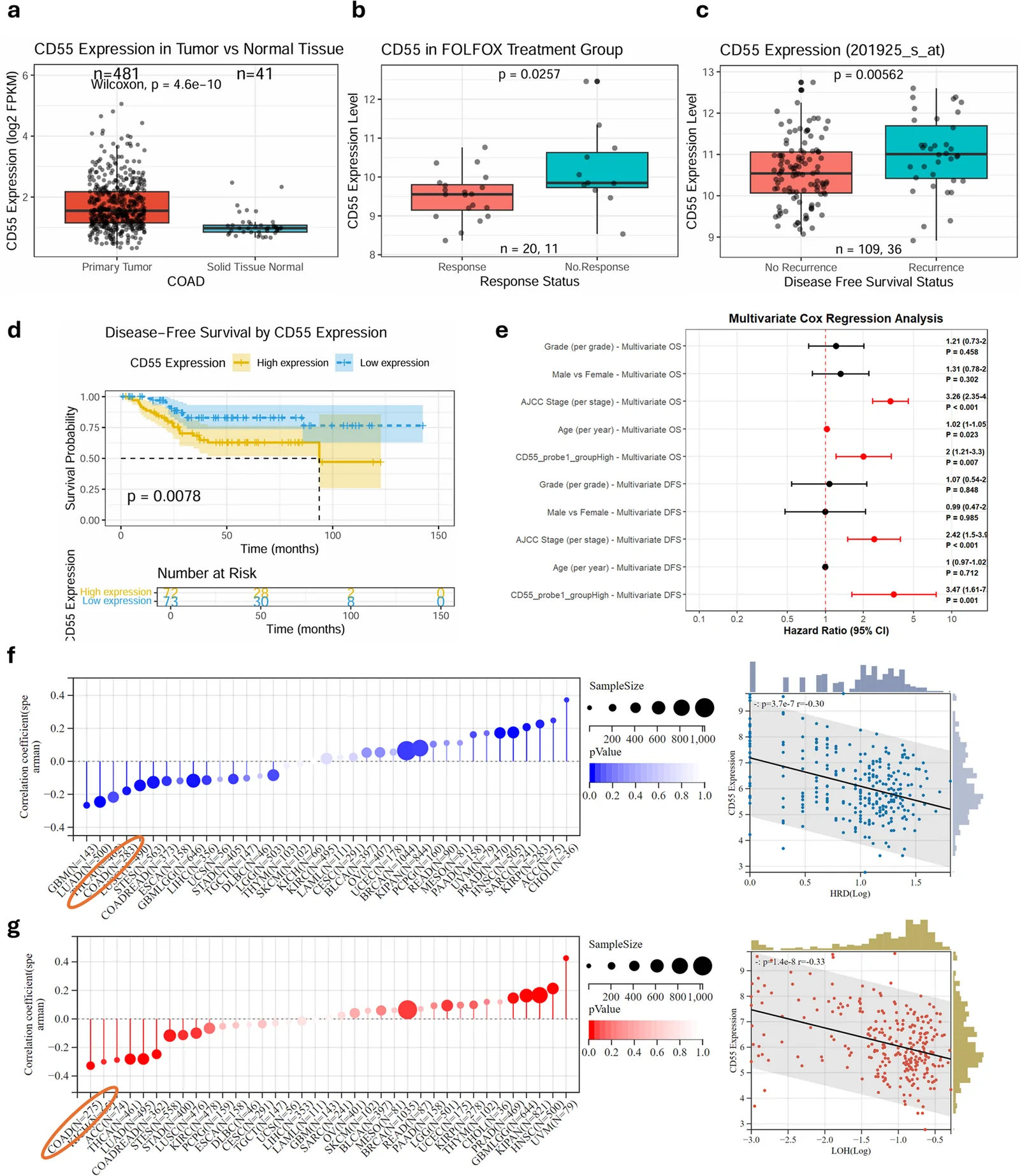
High CD55 associates with poor chemotherapy response and an HR-proficient clinical phenotype. (**a**) TCGA-COAD analysis: CD55 elevated in tumors (*n* = 481) vs. normal (*n* = 41) (*P* < 0.0001). Wilcoxon test. (**b**) GSE72970 (*n* = 31): FOLFOX non-responders (*n* = 11) showed higher CD55 than responders (*n* = 20) (*P* < 0.05). Limma moderated t-test. (**c**) GSE17536 (*n* = 145): CD55 elevated in recurrence group (*n* = 36) vs. recurrence-free (*n* = 109) (*P* < 0.01). Probe: 201925_s_at. Limma moderated t-test. (**d**) Kaplan-Meier disease-free survival analysis in GSE17536 cohort stratified by CD55 expression: high (*n* = 72) vs. low (*n* = 73); 25 vs. 11 recurrence events (*P* < 0.01, log-rank test).(**e**) Forest plot of multivariate Cox regression analysis showing hazard ratios with 95% confidence intervals for various clinical outcomes. CD55 high expression was identified as an independent risk factor for disease-free survival (HR = 3.47, 95% CI: 1.61–7.5, *P* < 0.05). Red dots indicate statistically significant associations (*P* < 0.05). Cox proportional hazards model for multivariate analysis. (**f**) Correlation between CD55 expression and HRD scores. Left: pan-cancer analysis (37 tumor types). Right: COAD-specific (*n* = 283); *R* = − 0.30, *P* = 3.7 × 10⁻⁷. (**g**) Correlation between CD55 expression and LOH scores. Left: pan-cancer. Right: COAD-specific (*n* = 275); *R* = − 0.33, *P* = 1.4 × 10⁻⁸. Spearman correlation analysis

To assess the clinical relevance of our findings, we analyzed CD55 expression patterns across multiple patient cohorts. TCGA-COAD analysis revealed significantly elevated CD55 expression in colorectal tumor tissues (*n* = 481) compared to adjacent normal tissues (*n* = 41) (*P* < 0.0001) (Fig. 3a). CD55 expression levels were independent of tumor stage, patient age, and gender (Figure S3a).

We next examined whether CD55 expression predicts chemotherapy response. Analysis of the GSE72970 dataset, comprising 31 patients treated with FOLFOX regimen, demonstrated that non-responders exhibited significantly higher CD55 expression compared to responders (*P* < 0.05) (Fig. 3b), directly supporting our in vitro findings that CD55 mediates oxaliplatin resistance.

The prognostic significance of CD55 was evaluated in the GSE17536 cohort (*n* = 145). Patients who experienced disease recurrence showed significantly higher CD55 expression levels compared to recurrence-free patients (*P* < 0.01) (Fig. 3c). Kaplan-Meier survival analysis demonstrated that patients with high CD55 expression (*n* = 72) had significantly shorter disease-free survival compared to those with low expression (*n* = 73) (25 vs. 11 recurrence events; *P* < 0.01) (Fig. 3d). Overall survival analysis revealed similar trends, with 30 deaths in the high expression group versus 14 in the low expression group (*P* < 0.01) (Figure S3b).

Multivariate Cox regression analysis confirmed CD55 as an independent prognostic factor for disease-free survival (HR = 3.47, 95% CI: 1.61–7.50, *P* < 0.01) and overall survival (HR = 1.99, 95% CI: 1.21–3.30, *P* < 0.01), maintaining significance alongside AJCC staging (Fig. 3e). These clinical data establish CD55 as both a predictive biomarker for chemotherapy response and a prognostic indicator in colorectal cancer.

To explore potential mechanisms underlying CD55-mediated resistance, we investigated the relationship between CD55 expression and DNA repair capacity using TCGA pan-cancer data. Intriguingly, CD55 expression showed a significant negative correlation with homologous recombination deficiency (HRD) scores across multiple cancer types, with colorectal adenocarcinoma (COAD) showing a particularly robust inverse relationship (Fig. 3f, left panel). In COAD samples specifically (*n* = 283), CD55 expression negatively correlated with HRD score (*R* = − 0.30, *P* = 3.7 × 10⁻⁷) (Fig. 3f, right panel).

Similarly, CD55 expression inversely correlated with loss of heterozygosity (LOH) scores, another marker of genomic instability associated with HR deficiency. Pan-cancer analysis confirmed this negative correlation across tumor types (Fig. 3g, left panel), with COAD samples (*n* = 275) showing significant inverse correlation (*R* = − 0.33, *P* = 1.4 × 10⁻⁸) (Fig. 3g, right panel).

These clinical correlations suggest that high CD55 expression is associated with HR-proficient, genomically stable tumors, providing rationale for subsequent functional investigation of CD55’s role in HR repair.

### CD55 contributes to homologous recombination repair capacity

**Fig. 4:**
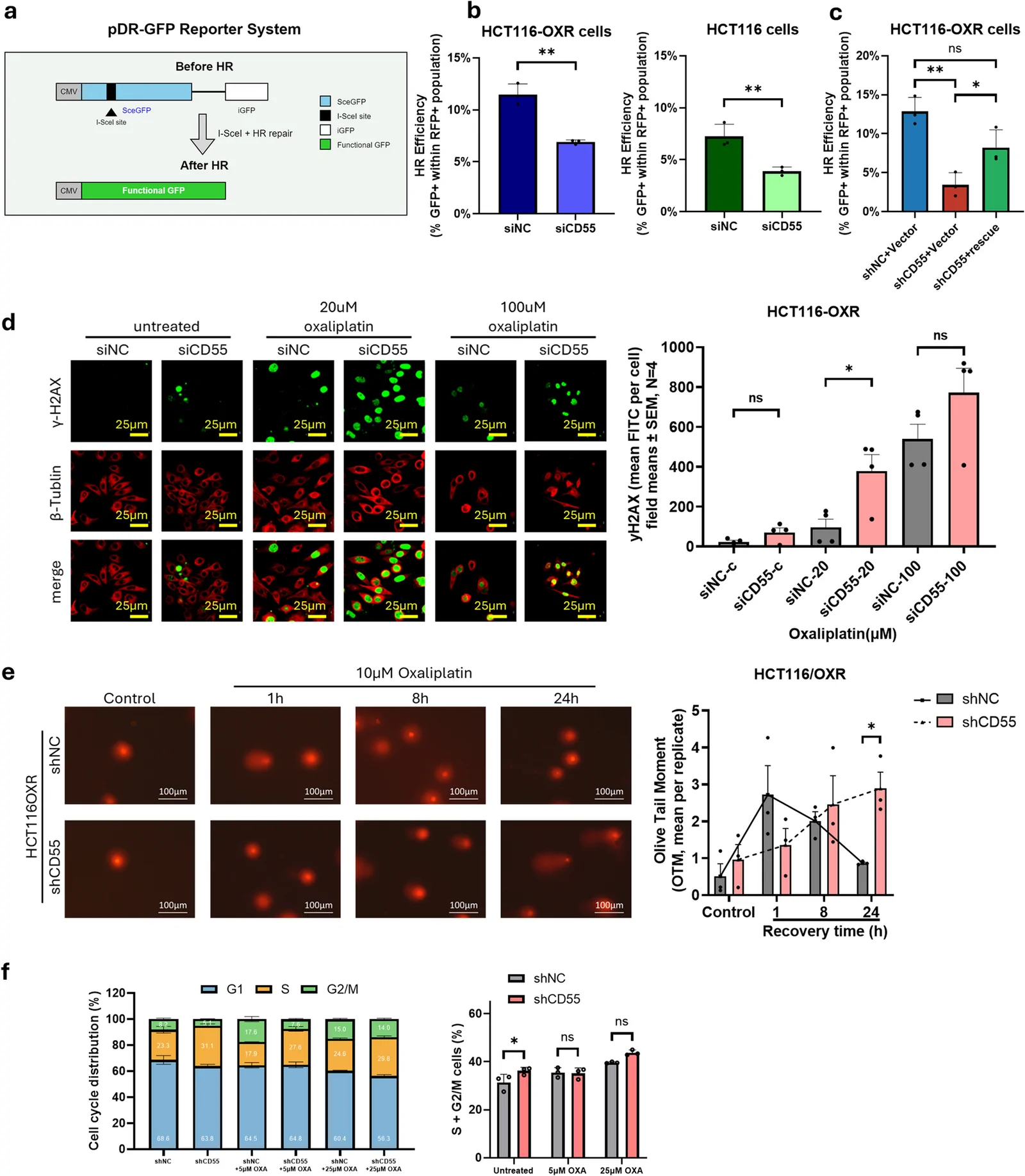
CD55 contributes to homologous recombination repair capacity. (**a**) Schematic of the DR-GFP reporter assay: I-SceI induces a DSB whose HR-mediated repair restores GFP. (**b**) HR efficiency in HCT116-DR and HCT116-OXR-DR cells transfected with siNC or siCD55. CD55 knockdown reduced HR efficiency from 7.24% to 3.90% (HCT116-DR, ~ 46%) and from 11.47% to 6.91% (HCT116-OXR-DR, ~ 40%); resistant cells showed ~ 1.6-fold higher baseline HR. (**c**) shRNA-resistant rescue in HCT116-OXR-DR cells. CD55 knockdown reduced HR efficiency from 12.85% (shNC + Vector) to 3.43% (shCD55 + Vector), and re-expression of shRNA-resistant CD55 restored it to 8.22%, no longer significantly different from shNC + Vector. (**d**) Representative γ-H2AX immunofluorescence images and quantification of mean nuclear γ-H2AX intensity in HCT116-OXR cells treated with oxaliplatin (0, 20, 100 µM, 48 h) following CD55 knockdown (siCD55) or control (siNC). Intensity increased dose-dependently and was elevated by CD55 knockdown, most clearly at 20 µM. Scale bar: 25 μm. (**e**) Representative alkaline comet images and Olive Tail Moment (OTM) quantification in HCT116-OXR cells treated with oxaliplatin (10 µM, 1 h) and analysed at 0 (untreated), 1, 8, and 24 h of recovery. shNC cells largely resolved damage, whereas CD55-depleted cells retained elevated OTM at 24 h (2.89 vs. 0.87, ~ 3.3-fold). Scale bar: 100 μm. (**f**) Combined S + G2/M (HR-permissive) fraction from PI cell-cycle analysis of HCT116-OXR cells (shNC vs. shCD55; untreated, 5, 25 µM oxaliplatin). CD55 knockdown did not reduce this fraction (untreated 31.4% → 36.2%; 5 µM 35.5% → 35.2%; 25 µM 39.6% → 43.8%). Data are mean ± SEM (*n* = 3 independent experiments; *N* = 4 for d). Statistical tests: (b, f) unpaired two-tailed Student’s t-test; (c) one-way ANOVA with Tukey’s post-hoc test; (d) two-way ANOVA with Šídák’s correction; (e) two-tailed Welch’s t-test (shCD55 vs. shNC at each recovery time point). *P < 0.05, **P < 0.01, ***P < 0.001; ns, not significant

To directly test whether CD55 regulates HR repair, we employed the DR-GFP reporter system, which quantifies HR efficiency by measuring GFP restoration following I-SceI–induced double-strand breaks (DSBs) (Fig. 4a). Single-copy reporter integration in both HCT116-DR and HCT116-OXR-DR clones was confirmed by RPP30-normalized genomic qPCR using two independent GFP primer sets (Figure S4a). Baseline analysis revealed that HCT116-OXR-DR resistant cells exhibited modestly elevated HR efficiency compared to parental HCT116-DR cells (11.47 ± 1.91% vs. 7.24 ± 0.61%, approximately 1.6-fold; *P* < 0.05), consistent with enhanced HR proficiency contributing to their capacity to resolve platinum-induced DNA damage. Importantly, CD55 knockdown significantly reduced HR efficiency in both cell contexts. In parental HCT116-DR cells, CD55 depletion decreased HR efficiency from 7.24% to 3.90% (~ 46% reduction, *P* = 0.0098). In HCT116-OXR-DR cells, HR efficiency dropped from 11.47% to 6.91% (~ 40% reduction, *P* = 0.0016) (Fig. 4b, S4b). To confirm that this HR defect was specifically attributable to CD55 depletion rather than off-target effects, we performed a rescue experiment by re-expressing shRNA-resistant CD55 in HCT116-OXR-DR cells. CD55 knockdown reduced HR efficiency from 12.85% (shNC + Vector) to 3.43% (shCD55 + Vector; *P* = 0.0022), whereas re-expression of shRNA-resistant CD55 (shCD55 + rescue) restored HR efficiency to 8.22% — significantly higher than shCD55 + Vector (*P* = 0.049) and no longer significantly different from shNC + Vector (*P* = 0.056) (Fig. 4c, S4c). These data confirm that CD55 specifically supports efficient HR-mediated repair capacity in both parental and resistant contexts.

We further assessed DNA damage accumulation using complementary approaches. Because 48-h oxaliplatin exposure produced predominantly diffuse pan-nuclear γH2AX staining rather than discrete foci, we quantified the mean nuclear γH2AX intensity per cell using an automated segmentation pipeline. γH2AX intensity increased dose-dependently (*P* < 0.0001), and CD55 knockdown significantly elevated overall γH2AX levels (*P* = 0.0042), with the clearest between-group difference at 20 µM oxaliplatin (*P* < 0.05) (Fig. 4d, S4d). Alkaline comet assays examining repair kinetics showed that control (shNC) cells largely resolved oxaliplatin-induced DNA damage over the recovery period, whereas CD55-depleted cells retained significantly elevated Olive Tail Moment at 24 h in both HCT116 (3.23 vs. 0.91, ~ 3.5-fold; Welch *p* = 0.033) and HCT116-OXR (2.89 vs. 0.87, ~ 3.3-fold; Welch *p* = 0.044) cells; the groups did not differ at the untreated, 1 h or 8 h time points (all Šídák-adjusted *P* > 0.2) (Fig. 4e, S4e). This pattern—comparable initial damage with a specific defect in late (24 h) resolution upon CD55 depletion—is consistent with compromised HR-mediated repair rather than altered damage sensing.

To exclude the possibility that the impaired HR efficiency and persistent γ-H2AX in CD55-depleted cells were secondary to cell cycle redistribution—given that HR is restricted to S/G2 phases—we analysed cell cycle distribution by flow cytometry. CD55 knockdown did not deplete the S/G2 compartment in any condition; rather, the combined S + G2/M fraction was preserved or modestly increased (e.g., 31.4% to 36.2% untreated, *P* < 0.05; 39.6% to 43.8% at 25 µM oxaliplatin, *P* = 0.075), with a relative accumulation of cells in S phase (Fig. 4f, S4f). Thus, the reduced HR efficiency and prolonged γ-H2AX in CD55-depleted cells reflect a genuine functional HR defect rather than a loss of HR-competent cells.

### Genotoxic stress induces stress-responsive CD55 and drives nuclear redistribution of a CD55 sub-pool

**Fig. 5:**
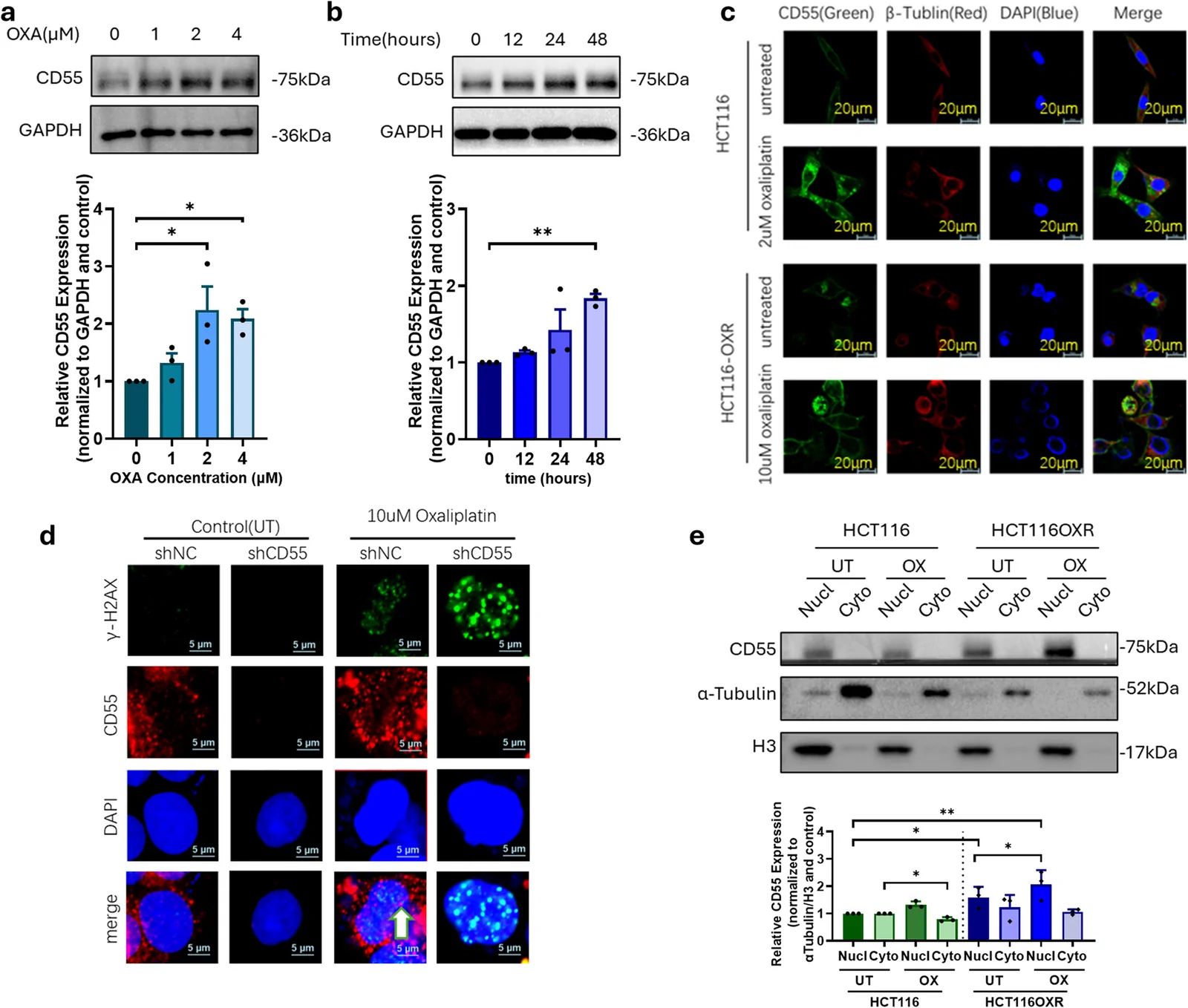
Genotoxic stress induces stress-responsive CD55 and drives nuclear redistribution of a CD55 sub-pool. **a** Dose-dependent CD55 upregulation in HCT116-OXR cells (oxaliplatin 0–4 µM, 48 h; Western blot): ~1.55-fold (1 µM), ~ 2.05-fold (2 µM) and ~ 1.99-fold (4 µM) relative to control (geometric mean, *n* = 3). **b** Time-dependent CD55 upregulation (oxaliplatin 2 µM; 0, 12, 24, 48 h), increasing progressively and maximal at 48 h (~ 1.83-fold). **c** Representative immunofluorescence of CD55 subcellular localization in HCT116 and HCT116-OXR cells ± oxaliplatin (2 µM for HCT116; 10 µM for HCT116-OXR). CD55 (green), β-tubulin (red), DAPI (blue). Approximately 40% of HCT116-OXR cells displayed nuclear CD55 after treatment. Scale bar, 20 μm; ≥100 cells per condition. **d** Validation of nuclear CD55 specificity by dual immunofluorescence using an independent anti-CD55 antibody (mouse monoclonal, sc-51733; green) co-stained with γ-H2AX (rabbit, #83307-2-RR; red) and DAPI (blue) in shNC versus shCD55 HCT116-OXR cells ± oxaliplatin (10 µM). Nuclear CD55 signal was reduced by 74.7% upon CD55 knockdown (*P* < 0.0001), whereas γ-H2AX foci were retained, confirming that the nuclear signal is CD55-specific. Scale bar, 20 μm; ≥100 cells per condition, *n* = 3. **e** Nuclear–cytoplasmic fractionation showing increased nuclear CD55 after oxaliplatin (1.3-fold in HCT116, 2.0-fold in HCT116-OXR; *P* < 0.05); resistant cells exhibited elevated baseline nuclear CD55. α-Tubulin, cytoplasmic marker; Histone H3, nuclear marker. Data are mean ± SEM; *n* = 3 independent experiments (≥ 100 cells per condition in c, d). For (**a**, **b**), log₂-transformed densitometry was analyzed by repeated-measures one-way ANOVA with Geisser–Greenhouse correction and a test for linear trend (dose *P* = 0.018; time *P* = 0.005), followed by Dunnett’s test versus control (significant at 2 and 4 µM in a, and at 48 h in b)

We observed that oxaliplatin induced CD55 as a stress-responsive gene. At the protein level, CD55 increased with oxaliplatin concentration to a plateau of approximately 2-fold at 2–4 µM (~ 2.05-fold at 2 µM; test for linear trend *P* = 0.018) and accumulated progressively over time to a maximum of ~ 1.83-fold at 48 h (test for linear trend *P* = 0.005) (Fig. 5a–b). At the transcript level, CD55 mRNA rose ~ 1.5-fold at 1 µM but fell back by the cytotoxic 4 µM dose, while likewise increasing progressively over time to ~ 2.9-fold at 48 h (trend *P* = 0.029) (Figure S5a-b), establishing CD55 as a bona fide stress-responsive gene. Furthermore, immunofluorescence revealed that following oxaliplatin treatment a sub-pool of CD55 redistributed from predominantly membrane localization into punctate cytoplasmic and nuclear/perinuclear signal, with approximately 40% of HCT116-OXR cells displaying nuclear CD55 (Fig. 5c, S5c). To confirm the specificity of this nuclear signal, we performed dual immunofluorescence with an independent anti-CD55 antibody of distinct host and clonality (mouse monoclonal, sc-51733) co-stained for γ-H2AX in shNC versus shCD55 HCT116-OXR cells (Fig. 5d, S5d). Nuclear CD55 was essentially abolished upon CD55 knockdown (reduced by 74.7%, *P* < 0.0001), whereas γ-H2AX foci were retained, confirming that the nuclear signal is CD55-specific. Nuclear-cytoplasmic fractionation confirmed increased nuclear CD55 (1.3-fold in parental, 2.0-fold in resistant cells; *P* < 0.05), with resistant cells showing elevated baseline nuclear levels (Fig. 5e). Together, these observations indicate that a sub-pool of CD55 undergoes stress-induced redistribution to the nucleus upon DNA damage.

### CD55 loss uncouples RAD51 recruitment from RAD51 expression and impairs HR execution under genotoxic stress

Our finding that γ-H2AX persists in CD55-depleted cells despite the presence of repair factors raised a central question: at which step does HR fail when CD55 is lost? We addressed this from the apex of the cascade downward, separating damage sensing from repair execution.

**Fig. 6:**
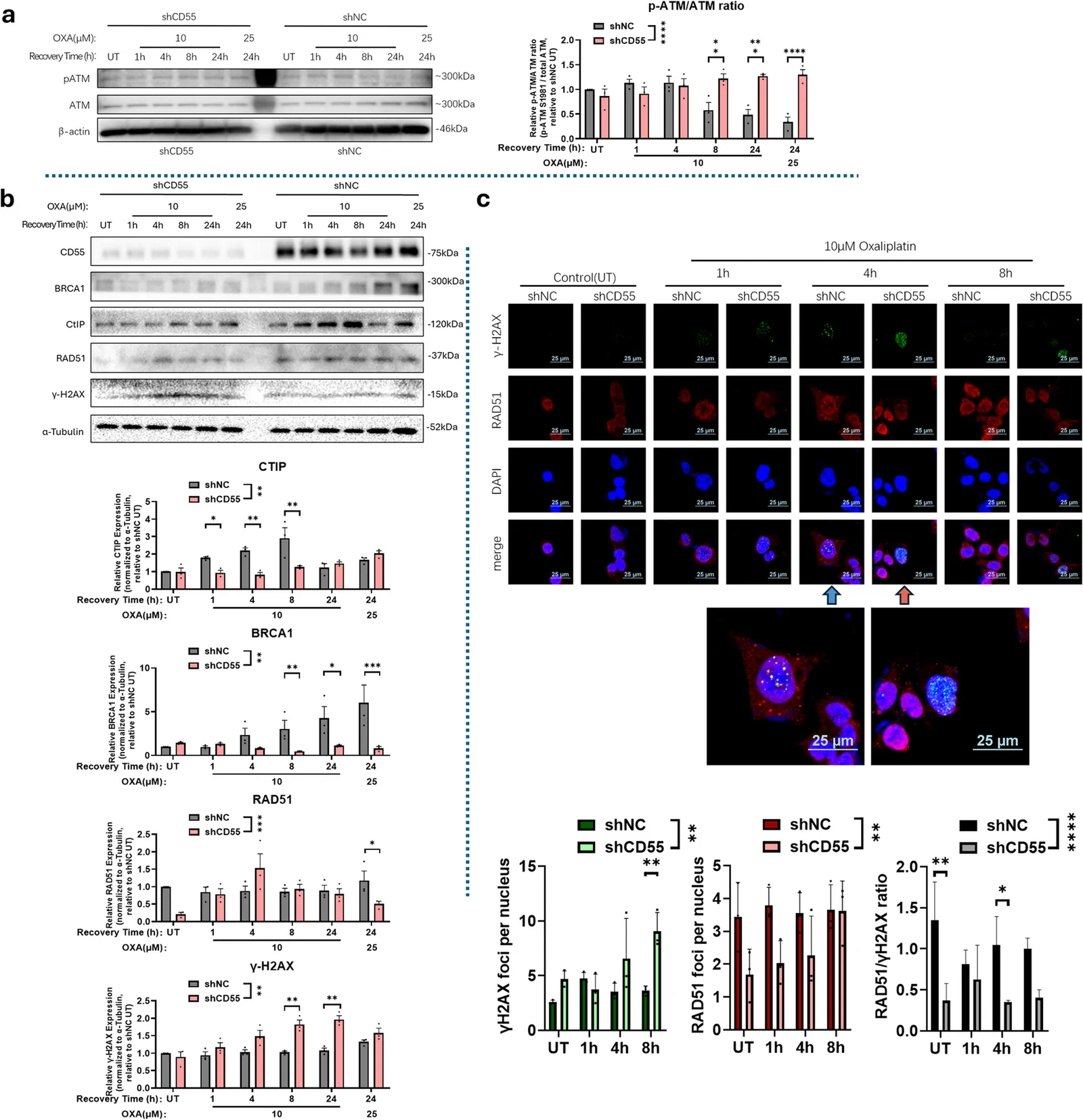
CD55 loss uncouples RAD51 recruitment from RAD51 expression and impairs HR execution. (**a**) Western blot of p-ATM (Ser1981) and total ATM across oxaliplatin recovery (10 µM, UT–24 h; 25 µM, 24 h); β-actin, loading control. Quantification: p-ATM/total-ATM, relative to shNC-UT within each blot. p-ATM/ATM was equivalent at UT–4 h (adjusted *P* > 0.7) and diverged thereafter, with shCD55 cells sustaining elevated p-ATM (8 h, *P* = 0.0025; 24 h/10 µM, *P* = 0.0003; 24 h/25 µM, *P* < 0.0001). Two-way ANOVA: genotype × time F(5,24) = 11.03, *P* < 0.0001; genotype F(1,24) = 26.20, *P* < 0.0001. Total ATM was unchanged (Figure S6a). (**b**) Western blot of CtIP, BRCA1, RAD51 and γ-H2AX in the same recovery series; α-tubulin, loading control; CD55 confirms knockdown. Quantification (relative to shNC-UT) compares shCD55 with shNC at each matched time/dose. In shNC cells, CtIP peaked ≈ 2.3-fold (4–8 h) and BRCA1 rose ≈ 5–6-fold (24 h); both were blunted in shCD55 cells. Baseline RAD51 was reduced in shCD55 cells (≈ 20% of control) but rose ≈ 7-fold after damage (4 h), whereas γ-H2AX accumulated persistently (8 h and 24 h/10 µM). (**c**) Dual immunofluorescence of γ-H2AX (green), RAD51 (red), DAPI (blue) and merge in untreated cells and after 10 µM oxaliplatin (1, 4, 8 h); boxed 4-h cells enlarged below. Scale bars, 25 μm. Quantification of RAD51 foci/nucleus, γ-H2AX foci/nucleus and the RAD51:γ-H2AX ratio: CD55 depletion reduced RAD51 foci (genotype *P* = 0.0029), increased γ-H2AX foci (genotype *P* = 0.0020; interaction *P* = 0.022; 9.05 vs. 3.63 at 8 h, *P* = 0.0028) and approximately halved the ratio (genotype *P* = 0.0001). 1,370 nuclei scored across three independent experiments. Data are mean ± SEM; *n* = 3. Two-way ANOVA with Šídák’s test. *P < 0.05, **P < 0.01, ***P < 0.001, ****P < 0.0001; ns, not significant

Damage sensing was intact. ATM autophosphorylation (Ser1981, normalized to total ATM) was not reduced by CD55 depletion—if anything it was sustained. p-ATM/ATM was equivalent in shNC and shCD55 cells early (UT–4 h; all adjusted *P* > 0.7) but diverged as repair proceeded: control cells resolved p-ATM, whereas CD55-depleted cells held it elevated (8 h, *P* = 0.0025; 24 h/10 µM, *P* = 0.0003; 24 h/25 µM, *P* < 0.0001), with a significant genotype × time interaction (F(5,24) = 11.03, *P* < 0.0001) and unchanged total ATM (Fig. 6a, S6a). Sustained apical signaling in the absence of resolution indicates a downstream, not a sensing, defect.

Time-course immunoblotting localized that defect (Fig. 6b, S6b). Relative to matched shNC controls, CD55 depletion blunted the damage-induced inductions of CtIP (≈ 2.3-fold, 4–8 h in controls) and BRCA1 (5–6-fold, 24 h in controls), indicating impaired upstream coordination of resection and mediator loading. RAD51 was the informative exception: although baseline RAD51 was low in CD55-depleted cells (≈ 20% of control), damage drove a compensatory ≈ 7-fold rise, restoring total protein. Yet γ-H2AX still accumulated, signaling that abundant RAD51 protein was insufficient for repair.

To test whether RAD51 was functionally engaged independent of its abundance, we co-stained RAD51 and γ-H2AX nuclear foci across recovery (Fig. 6c). During this early window (≤ 8 h after 10 µM oxaliplatin), both markers resolved into discrete, countable foci—distinct from the diffuse pan-nuclear γ-H2AX produced by prolonged high-dose exposure (48 h, Fig. 4d), which we quantify as mean nuclear intensity—and were therefore enumerated per nucleus. Despite the compensatory protein increase, CD55-depleted cells formed fewer RAD51 foci (two-way ANOVA, genotype *P* = 0.0029) and accumulated γ-H2AX foci (genotype *P* = 0.0020; interaction *P* = 0.022; 9.05 vs. 3.63 foci/nucleus at 8 h, Šídák *P* = 0.0028). This RAD51-foci reduction was not secondary to cell-cycle redistribution, as CD55 depletion did not diminish the HR-permissive S/G2 fraction. The RAD51:γ-H2AX ratio—productive loading relative to damage—was approximately halved (genotype *P* = 0.0001). RAD51 recruitment, not RAD51 abundance, is therefore the rate-limiting failure when CD55 is lost.

Together these data resolve the paradox: CD55-depleted cells sense damage normally—indeed ATM stays active—but cannot execute repair, because CtIP/BRCA1 coordination is blunted and RAD51 loading is uncoupled from RAD51 expression. CD55 thus operates between ATM activation and productive RAD51 recruitment to support HR execution under genotoxic stress.

### Loss of CD55 converts the oxaliplatin–olaparib interaction from antagonism to synergy in resistant cells

**Fig. 7:**
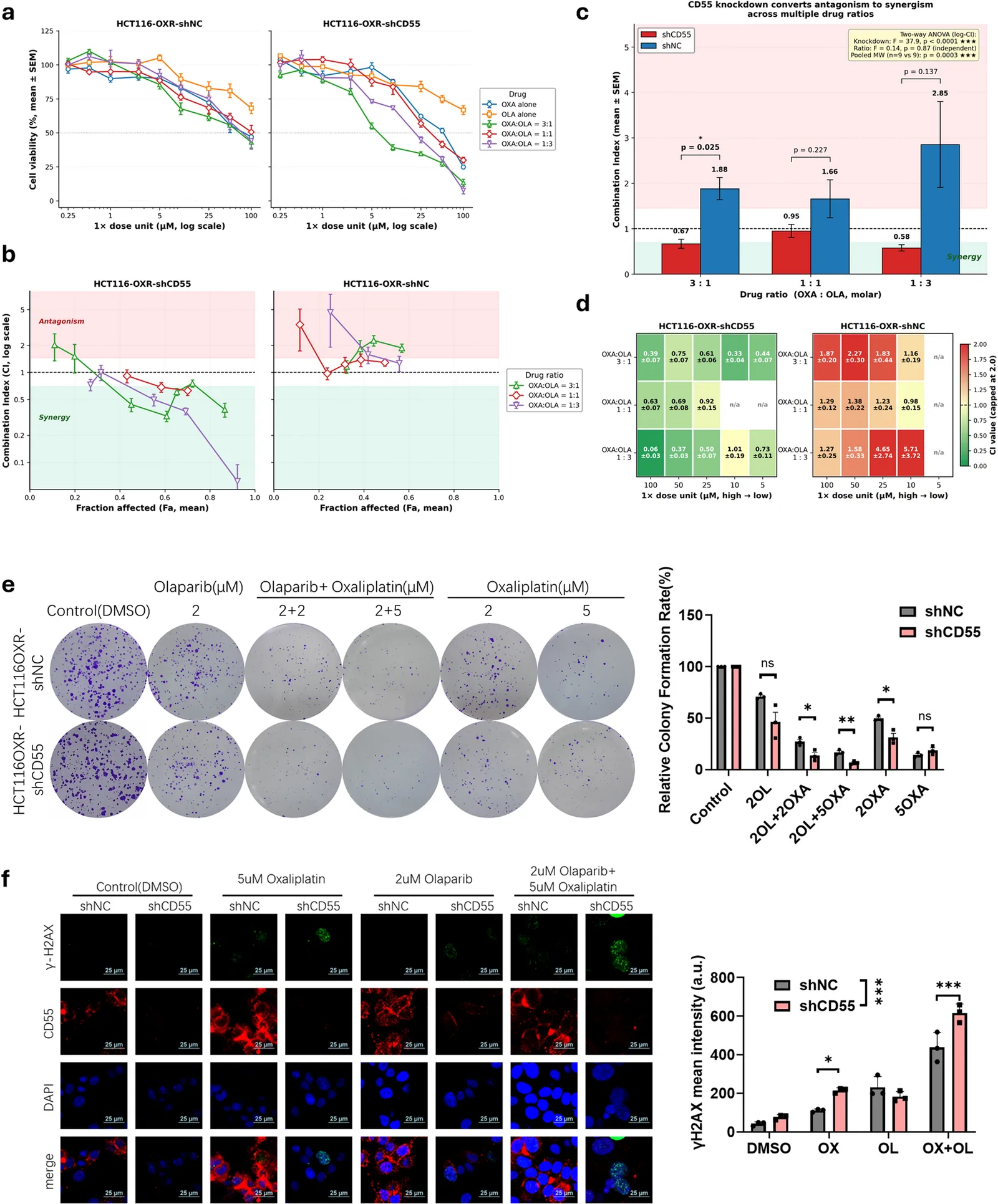
CD55 depletion converts the oxaliplatin–olaparib interaction from antagonism to synergy in oxaliplatin-resistant CRC cells. (**a**) Dose–response of oxaliplatin (OXA) and olaparib (OLA), alone and combined at fixed molar ratios (OXA: OLA = 3:1, 1:1, 1:3), in HCT116-OXR cells expressing control (shNC) or CD55-targeting (shCD55) shRNA (48-h CCK-8; 1× dose unit in µM, log scale). Olaparib showed negligible single-agent activity in both backgrounds. (**b**) Combination index (CI) versus fraction affected (Fa) for each ratio in shCD55 and shNC cells; shaded bands mark synergy (CI < 1) and antagonism (CI > 1). CI is interpreted only at Fa > 0.1. shCD55 points fall predominantly within the synergy range, whereas shNC points lie at or above additivity across all ratios. (**c**) Mean CI across the three ratios; CD55 knockdown lowered CI at every ratio, reaching synergy at 3:1 and 1:3 and additivity at 1:1. (**d**) Per-dose CI heatmap (colour capped at 2.0); high-CI cells at low-effect doses reflect unstable low-Fa estimates and are excluded from the summary CI (Supplementary Table S3). (**e**) Long-term clonogenic survival of HCT116-OXR cells treated with OLA, OXA, or their combinations; representative colonies and quantification. (**f**) Mean nuclear γ-H2AX intensity after 48-h DMSO, OXA (5 µM), OLA (2 µM), or OXA + OLA; representative confocal images (γ-H2AX, DAPI; scale bar 25 μm) and quantification. Data are mean ± SEM, *N* = 3 biological replicates (f, ≥ 100 nuclei/condition); CI averaged over Fa > 0.1. Statistics: (c) Welch’s two-tailed t-test (3:1 *P* = 0.025; 1:1 *P* = 0.227; 1:3 *P* = 0.137); two-way ANOVA on log-CI — knockdown F = 37.9, *P* < 0.0001; ratio F = 0.14, *P* = 0.87 (no ratio effect or interaction); replicates pooled across ratios, Mann–Whitney *n* = 9 vs. 9, *P* = 0.0003. (e) Student’s t-test with Holm–Šídák correction. (f) Two-way ANOVA with Šídák correction. *P < 0.05, **P < 0.01, ***P < 0.001; ns, not significant

Because CD55 depletion impairs HR and disrupts coordinated DDR signaling, we tested whether it could create a “BRCAness-like” state that sensitizes HR-proficient cells to PARP inhibition. Using the median-effect (Chou–Talalay) method across three fixed molar ratios (OXA: OLA = 3:1, 1:1, 1:3; three biological replicates; CI interpreted only at fa > 0.1), we found that the mode of drug interaction was governed by CD55 status (Fig. 7a and b). In CD55-intact HCT116-OXR cells (shNC), oxaliplatin and olaparib were antagonistic across all three ratios (mean CI 1.66–2.85), consistent with efficient HR-mediated resolution of platinum lesions. CD55 depletion reversed this pattern, lowering mean CI to 0.67 (3:1), 0.95 (1:1), and 0.58 (1:3) — synergy at 3:1 and 1:3, additivity at 1:1 (Fig. 7c and d). The shift reached significance at 3:1 (shCD55 0.67 vs. shNC 1.88, *P* = 0.025, Welch’s t-test) and was directionally consistent at the other ratios (Fig. 7c; Figure S7a; Supplementary Table S3), with synergy most pronounced at intermediate-to-high effect (3:1: CI 0.33–0.44 at fa ≈ 0.45–0.61); the weak 1:1 interaction indicates the conversion is dose-ratio–dependent.

This conversion was specific to the resistant background. In parental cells, olaparib had negligible single-agent activity and the 1:1 combination remained antagonistic irrespective of CD55 status (all *P* > 0.05) (Figure S7b), indicating that the intact, stress-adapted HR of HCT116-OXR cells is the relevant target of CD55 loss.

Two orthogonal assays linked the CI shift to a functional repair defect. First, long-term colony formation (Fig. 7e; Figure S7c): CD55 knockdown significantly potentiated the combination in HCT116-OXR cells (2 + 2 µM, 50.4% reduction, *P* < 0.05; 2 + 5 µM, 60.1% reduction, *P* < 0.01), whereas olaparib monotherapy gave no significant effect (*P* = 0.059). Second, nuclear γ-H2AX intensity across DMSO, OXA, OLA, and OXA + OLA arms (Fig. 7f; Figure S7d) was treatment- and CD55-dependent (two-way ANOVA interaction F(3,16) = 9.023, *P* = 0.0010), peaking in shCD55 + OXA + OLA (616.1 vs. 438.1 a.u. in shNC, *P* = 0.0002) and showing no CD55-dependent increase under olaparib alone. Together, these data show that CD55 loss drives supra-additive, unresolved DNA damage when oxaliplatin and olaparib are combined — providing the per-cell, DNA-damage readout that underlies the population-level CI synergy.

### Targeting CD55 enhances combination efficacy and improves tolerability in vivo

**Fig. 8:**
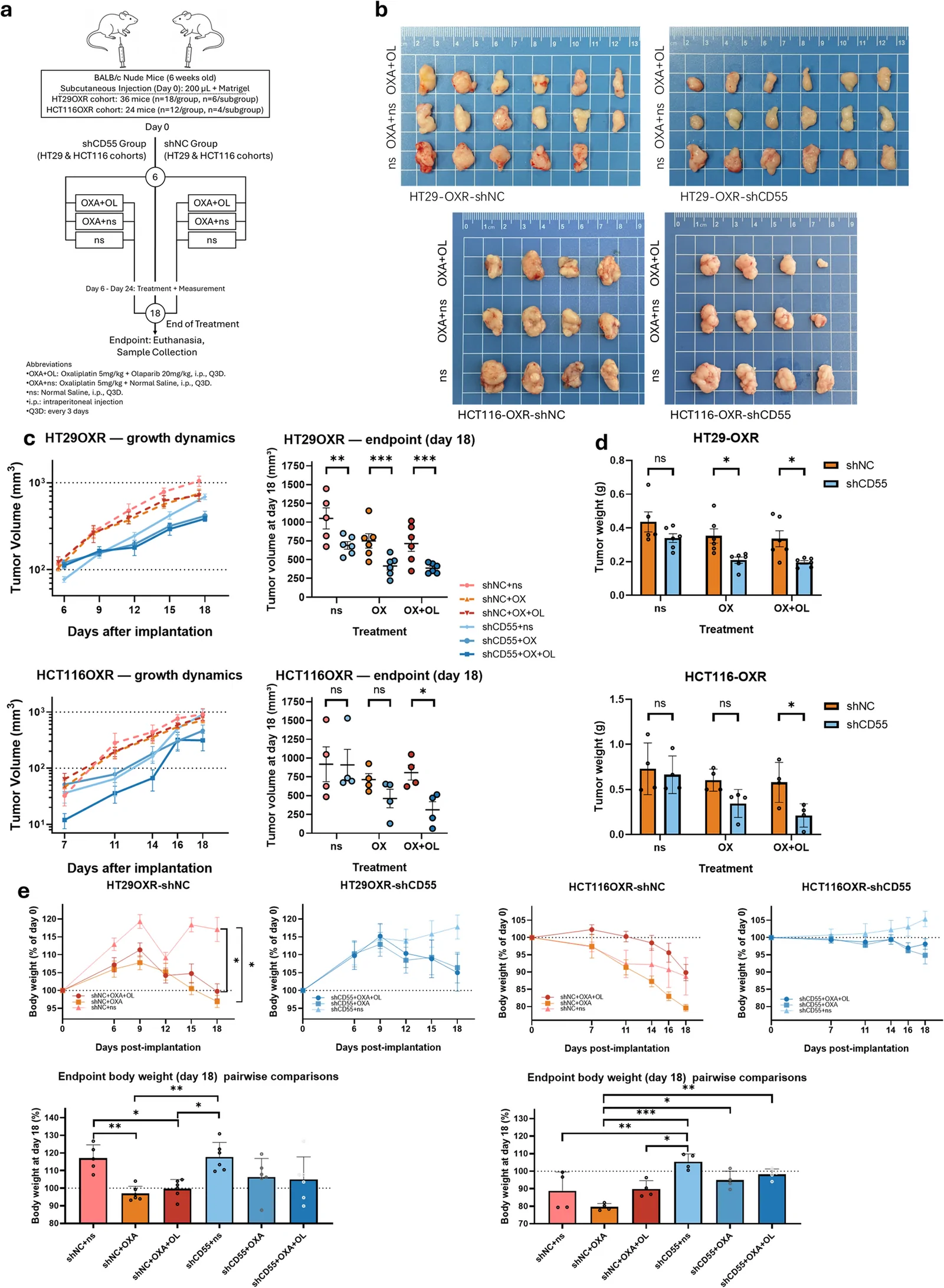
Targeting CD55 enhances combination efficacy and improves tolerability in vivo. (**a**) Experimental design. BALB/c nude mice (6 weeks old) bearing subcutaneous HT29-OXR (*n* = 6/group) or HCT116-OXR (*n* = 4/group) xenografts stably expressing shCD55 or control shRNA (shNC) received vehicle (ns), oxaliplatin (OXA, 5 mg/kg), or oxaliplatin plus olaparib (OXA + OL, 5 + 20 mg/kg) by intraperitoneal injection every 3 days from day 6 to day 18. (**b**) Representative excised tumors from all treatment arms in both models at endpoint. (**c**) Tumor-volume growth dynamics (left) and endpoint tumor volume at day 18 (right) for HT29-OXR (top) and HCT116-OXR (bottom). (**d**) Final tumor weights for shNC versus shCD55 across treatment arms in HT29-OXR (top) and HCT116-OXR (bottom). (**e**) Body weight (% of day 0) over time for each model and genotype (top) and day-18 pairwise comparisons (bottom). Statistics: Tumor growth was modeled by a linear mixed-effects model accounting for repeated measurements and per-animal random effects, cross-validated in GraphPad Prism. Body-weight trajectories were analyzed by two-way repeated-measures ANOVA with Tukey’s post hoc test. Data are presented as mean ± SEM. *P < 0.05, **P < 0.01, ***P < 0.001; ns, not significant

To validate these findings in vivo, we established subcutaneous xenografts in two independent oxaliplatin-resistant backgrounds—an adequately powered HT29-OXR model (*n* = 6 per group) and the original HCT116-OXR model (*n* = 4 per group) (Fig. 8a; representative tumors, Fig. 8b). Tumor growth was analyzed by a linear mixed-effects model accounting for repeated measurements and per-animal random effects, cross-validated in GraphPad Prism; the complete statistical summary (model output, endpoint contrasts and per-group normality tests) is provided in Supplementary Table S4, and model diagnostics and individual growth trajectories for every animal are provided in Figure S8a–c.

CD55 knockdown suppressed tumor growth in both models. In HT29-OXR, CD55 status exerted a highly significant overall effect (*P* < 0.001), with reduced endpoint tumor volume in every arm—including under oxaliplatin monotherapy (vehicle, *P* = 0.008; oxaliplatin, *P* < 0.001; combination, *P* < 0.001). The overall effect was reproduced in HCT116-OXR (*P* = 0.005), reaching arm-level significance in the combination arm (*P* = 0.020) and trending concordantly elsewhere (Fig. 8c). Final tumor weights mirrored these results: in HT29-OXR, weight was reduced under both oxaliplatin (*P* = 0.026) and combination therapy (*P* = 0.030), with a significant overall CD55 effect (*P* = 0.0002); in HCT116-OXR, the reduction reached significance under combination therapy (0.21 g vs. 0.58 g, *P* = 0.048) and followed the same trend under oxaliplatin (0.34 g vs. 0.60 g) (Fig. 8d).

Importantly, this enhanced efficacy came without added systemic toxicity. CD55 depletion was not itself toxic—in HT29-OXR, vehicle-treated shCD55 and shNC animals gained weight identically (day 18: 117.8% vs. 117.1%, *P* = 0.89)—yet under treatment shCD55 animals were protected from the chemotherapy-associated weight loss that emerged in shNC animals only after oxaliplatin ± olaparib (Fig. 8e). Because the genotypes were matched at baseline, CD55 loss does not compound oxaliplatin toxicity but instead widens the therapeutic window. In HCT116-OXR, shCD55 animals were likewise heavier than shNC animals, though here the difference was already present in vehicle-treated mice (88.8% vs. 105.4%, *P* = 0.047), consistent with reduced tumor-burden–associated cachexia from superior tumor control.

Quantitative IHC confirmed sustained CD55 knockdown across all arms in both cohorts, using two independent anti-CD55 antibodies (HCT116-OXR, Figure S8d; HT29-OXR, Figure S8e). Consistent with the in vitro CD55–BRCA1 axis, BRCA1 was markedly reduced in shCD55 HT29-OXR tumors at baseline (Tukey-adjusted *P* = 0.002; Figure S8e). In HCT116-OXR, Ki-67 reached its lowest level in the shCD55 + combination group (Tukey-adjusted *P* = 0.038 vs. shNC + vehicle; Figure S8d). γH2AX was elevated in shCD55 tumors in both cohorts (Figure S8d, e), and cleaved caspase-3 was selectively induced under combination therapy in HT29-OXR (Figure S8e).

## Discussion and conclusion

**Fig. 9:**
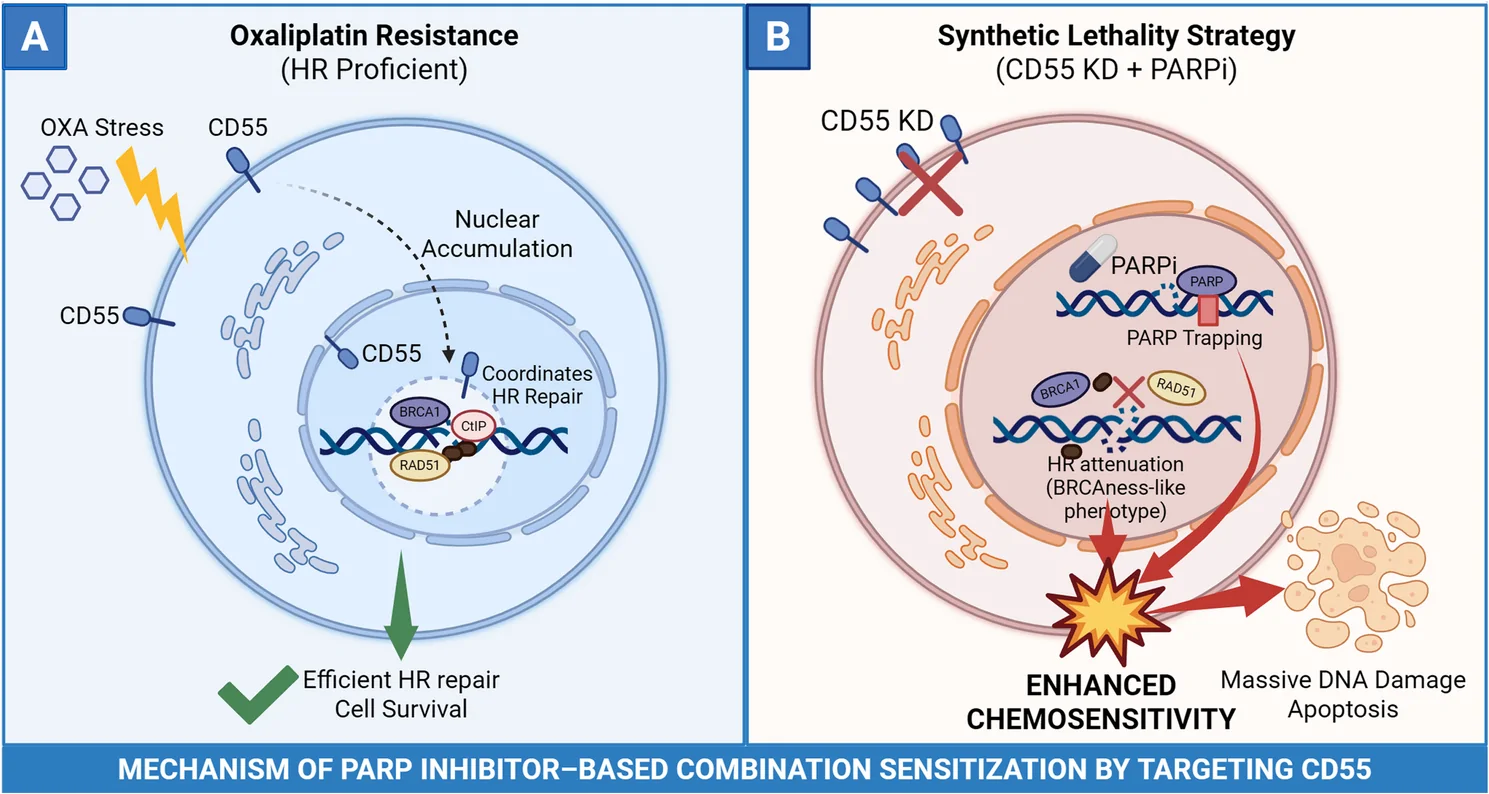
Proposed model of stress-induced CD55 signaling in supporting homologous recombination capacity and the PARP inhibitor–based combination-sensitization strategy upon CD55 inhibition. (**a**) Under genotoxic stress, CD55 is induced and supports HR capacity by promoting coordinated BRCA1, CtIP, and RAD51 dynamics, enabling efficient resolution of platinum-induced DNA damage and promoting cell survival. (**b**) CD55 depletion disrupts this stress-adaptive response, impairing HR repair and producing a partial HR deficit (‘BRCAness-like’ state). Combined with PARP inhibition, this enhanced sensitivity leads to accumulation of unresolved DNA damage and apoptosis

Acquired resistance to oxaliplatin represents a major clinical challenge in colorectal cancer management, yet the stress-responsive signaling programs that enable tumor adaptation to platinum-induced genotoxic stress remain incompletely understood [27]. In this study, we identify CD55 as a stress-induced signaling regulator that supports homologous recombination (HR) capacity and promotes oxaliplatin resistance in colorectal cancer. We show that CD55 is acutely induced by platinum treatment and undergoes intracellular redistribution, including nuclear accumulation, following DNA damage. Functional disruption of this stress-adaptive CD55 pathway compromises HR efficiency and sensitizes oxaliplatin-resistant cells to PARP inhibitor–based combination therapy, offering a mechanistically informed strategy to overcome platinum resistance in colorectal cancer (Fig. 9).

### CD55 as a stress-responsive signaling mediator of oxaliplatin resistance

The identification of CD55 as a resistance mediator is supported by convergent evidence from multiple independent sources. CD55 upregulation was consistently observed across our RNA-sequencing data and two publicly available datasets (GSE119603, GSE138647), with validation at both mRNA and protein levels. This reproducibility across different experimental systems strengthens confidence in CD55 as a bona fide resistance factor rather than a context-specific artifact. More importantly, we provide direct evidence that CD55 is not merely passively overexpressed in resistant cells but is acutely induced by oxaliplatin treatment in a dose- and time-dependent manner. This stress-responsive induction, coupled with the observation of nuclear accumulation following DNA damage, positions CD55 as an adaptive stress-responsive signaling component that contributes to cellular responses to genotoxic insults.

The functional significance of CD55 in chemoresistance is underscored by the universal sensitization observed upon its depletion. CD55 knockdown enhanced oxaliplatin sensitivity in all seven colorectal cancer cell lines tested, regardless of microsatellite status (MSI-H: HCT116, RKO, LOVO; MSS: HT29, SW480), p53 status (wild-type: HCT116, LOVO, RKO; mutant: HT29, SW480), or BRAF mutation status [28]. The magnitude of sensitization varied—from approximately 1.2-fold in HT29-OXR to 5.4-fold in RKO cells—potentially reflecting context-dependent interactions between CD55 depletion and underlying DNA repair deficiencies, such as mismatch repair defects. Despite this quantitative variability, at least one siRNA produced significant sensitization in every line and the direction of effect was uniform across diverse molecular subtypes, supporting a general role for CD55 in promoting oxaliplatin resistance.

The sensitization effect of CD55 depletion is associated with augmented apoptotic responses. Flow cytometry analysis revealed that CD55 knockdown significantly enhanced oxaliplatin-induced apoptosis, with particularly pronounced effects on late apoptosis in resistant cells (19.7% vs. 9.2%). At the molecular level, CD55 depletion reduced BCL-2 expression while increasing BAX levels, resulting in a decreased BCL-2/BAX ratio that favors mitochondrial apoptotic pathway activation. These findings are consistent with previous reports linking CD55 to pro-survival signaling in other cancer types [29], and suggest that CD55 contributes to chemoresistance through both support of DNA repair capacity and modulation of apoptotic signaling. This dual function—promoting damage resolution while suppressing cell death—may explain the robust and universal sensitization observed across genetically diverse cell lines.

The asymmetric phenotype—robust sensitization upon CD55 depletion versus a modest, non-significant IC50 shift upon overexpression—indicates that CD55 is necessary but not sufficient for oxaliplatin resistance. Several non-exclusive factors may contribute to this asymmetry. First, parental HCT116 cells already express functional CD55, and its contribution to HR support may approach a functional ceiling, such that further elevation produces limited additional gain. Second, our data suggest that CD55-mediated resistance depends not only on protein abundance but also on stress-induced functional activation, including the nuclear redistribution observed in chronically adapted resistant cells. Whether ectopic CD55 overexpression in cells lacking chronic platinum adaptation achieves an equivalent activated state remains to be formally tested. Importantly, this asymmetry does not diminish the translational relevance of our findings, because loss-of-function is the clinically actionable direction. The strong and reproducible sensitization observed across seven genetically diverse CRC cell lines supports CD55 as a tractable vulnerability in resistant tumors.

### CD55 supports homologous recombination capacity under genotoxic stress

A key finding of this study is the functional association between CD55 and HR capacity. In TCGA-COAD samples, CD55 expression negatively correlated with both HRD scores (*R* = − 0.30) and LOH scores (*R* = − 0.33), suggesting that CD55-high tumors tend to maintain genomic stability through proficient HR [30]. This clinical correlation was functionally validated using the DR-GFP reporter system, which directly demonstrated that CD55 knockdown induces a partial but reproducible reduction in HR efficiency (~ 46% in parental and ~ 40% in resistant HCT116 cells). The observation that resistant cells exhibit a moderately elevated baseline HR capacity provides a partial functional explanation for their enhanced ability to resolve platinum-induced DNA damage. Although the magnitude of HR impairment upon CD55 depletion is more modest than the near-complete HR collapse typically reported for BRCA1/2-mutated cells [18], it is nonetheless functionally sufficient to shift antagonistic oxaliplatin–olaparib interactions toward synergy in the resistant context (mean CI 0.58–0.95 across ratios). This quantitative distinction underlies our operational use of the term “BRCAness-like” rather than “BRCAness” — denoting a partial, drug-sensitizing HR deficit rather than a complete BRCA-deficient phenotype.

The characterization of DDR protein dynamics revealed that CD55 depletion is associated with altered DNA damage response signaling. In CD55-depleted cells, BRCA1 induction following DNA damage was attenuated, CtIP response was diminished, and γ-H2AX accumulated persistently despite compensatory RAD51 elevation. This pattern—impaired upstream coordination with compensatory but ineffective downstream activation—suggests that CD55 influences early coordination of DDR signaling rather than acting as a core repair factor. Dual foci analysis showed that, although RAD51 protein rose after damage in CD55-depleted cells, this was not matched by functional RAD51 foci while γH2AX accumulated, indicating the compensatory RAD51 induction is functionally insufficient. The temporal analysis using comet assays further supports this interpretation: CD55-depleted cells showed normal initial damage induction but impaired resolution over time, consistent with compromised repair capacity rather than altered damage sensing.

The stress-induced redistribution of a CD55 sub-pool to the nucleus following DNA damage provides potential insight into how a predominantly membrane-anchored protein might influence intracellular DNA repair processes. GPI-anchored proteins are canonically tethered to the outer plasma membrane, suggesting that nuclear entry requires proteolytic release and active trafficking. Recent work in ovarian cancer demonstrates that cell-surface CD55 can be cleaved, internalized, and trafficked to the nucleus, where it binds the epigenetic regulator ZMYND8 and modulates H3K27 trimethylation to promote platinum resistance [26]. While the precise trafficking mechanism and nuclear binding partners in CRC remain to be defined, these findings suggest that nuclear CD55 may link membrane stress sensing to chromatin-level regulation of DNA repair gene expression. This model is based on functional and correlative evidence and does not imply that CD55 is a canonical chromatin regulator. Future studies employing membrane-marker-controlled fractionation, targeted glycoproteomics, and proximity-labeling will be necessary to definitively confirm the modest nuclear pool and identify the nuclear interactome of CD55 in colorectal cancer cells.

Regardless of the precise molecular mechanism, our functional data consistently demonstrate that CD55 supports HR capacity, as evidenced by reduced HR efficiency, impaired DDR coordination, prolonged damage accumulation, and enhanced drug sensitivity upon CD55 depletion. These findings position CD55 as a stress-responsive modulator of HR competence in colorectal cancer.

### Clinical and therapeutic implications

The translational significance of our findings is supported by clinical correlations across multiple patient cohorts. In the GSE72970 dataset, FOLFOX non-responders exhibited significantly higher CD55 expression than responders, directly supporting our functional findings in a clinical context. Furthermore, high CD55 expression independently predicted worse disease-free survival (HR = 3.47) and overall survival (HR = 1.99) in multivariate analysis, establishing CD55 as a prognostic biomarker beyond its role in treatment response. The stronger association with recurrence risk compared to mortality may reflect CD55’s multifaceted contributions to tumor progression, including effects on invasive capacity observed in our in vitro studies [31, 32]. These findings suggest that CD55 assessment could identify high-risk patients who might benefit from intensified surveillance or enrollment in trials of CD55-targeted combination strategies.

From a therapeutic standpoint, a central implication of our findings is the interaction between CD55 suppression and PARP inhibition. PARP inhibitors are highly effective in HR-deficient backgrounds but show limited efficacy in HR-proficient colorectal cancers, where germline BRCA mutations are rare (< 5%) [13, 14]. In oxaliplatin-resistant cells with intact CD55, oxaliplatin combined with olaparib was antagonistic across all tested ratios, consistent with efficient resolution of platinum-induced lesions by proficient HR machinery. CD55 depletion shifted this interaction away from antagonism toward synergy (mean CI 0.58–0.95), reaching synergy (CI < 0.7) at the 3:1 and 1:3 ratios and at intermediate-to-high effect levels, with the difference from control being statistically significant at the 3:1 ratio (shCD55 0.67 vs. shNC 1.88, *P* = 0.025, Welch’s t-test). Notably, in parental and CD55-overexpressing cells—where olaparib displayed negligible single-agent activity, as expected for an HR-proficient, PARPi-insensitive context—the combination remained antagonistic at the 1:1 ratio; CD55 depletion lowered the combination index without reaching significance. This pattern indicates that the antagonism-to-synergy shift is most clearly unmasked in the resistant, high-HR background in which olaparib retains measurable activity, rather than representing a uniform synergy across all genetic contexts. Mechanistically, the shift is consistent with a convergent assault on DNA repair: oxaliplatin induces crosslinks and double-strand breaks that require HR for resolution, while PARP inhibition blocks single-strand break repair; in CD55-depleted cells with partially compromised HR, this combined burden is less efficiently resolved.

The stress-responsive nature of CD55 induction may have additional therapeutic implications. Tumors that mount robust CD55 responses to initial chemotherapy exposure may be particularly dependent on this pathway and thus more vulnerable to CD55-targeted interventions during subsequent treatment cycles. This suggests a potential strategy of sequential or adaptive therapy, where CD55 status is monitored during treatment to identify optimal windows for combination intervention.

Several therapeutic modalities could be considered for clinical translation. Monoclonal antibodies targeting CD55 have shown promise in preclinical models, with a chimeric anti-CD55 antibody demonstrating complement activation, tumor growth suppression, and synergy with 5-FU chemotherapy in CRC [33]. RNA-based knockdown approaches, as demonstrated in our shRNA studies, provide proof-of-concept that CD55 silencing achieves the desired sensitization phenotype.

An important observation was the improved treatment tolerability when CD55 was inhibited. Control mice receiving oxaliplatin experienced weight loss approaching the 80% safety threshold, whereas CD55-depleted tumor-bearing mice maintained near-normal weight during both monotherapy and combination treatment. CD55 depletion was associated with preserved body weight under chemotherapy, suggesting it does not aggravate—and may mitigate—treatment-related toxicity. We interpret this as a promising trend rather than definitive evidence of a tumor-selective therapeutic index, which will require dedicated toxicity assessment, it may relate to differential CD55 dependence between tumor and normal tissues, or to reduced systemic inflammation when tumor burden is effectively controlled. This favorable therapeutic index supports continued development of CD55-targeted strategies.

### Limitations and future directions

Several limitations warrant acknowledgment. First, while we establish a functional requirement for CD55 in sustaining HR capacity under stress conditions through multiple orthogonal approaches, the precise molecular mechanism by which CD55 influences DNA repair remains incompletely defined. Whether CD55 directly interacts with repair machinery, modulates transcription of HR genes, or functions through chromatin modification requires dedicated investigation. We also did not assay DNA end resection directly; RPA32/pRPA foci and native BrdU/IdU assays are a planned next step. Importantly, this mechanistic gap parallels early PARP inhibitor development, where clinical success was achieved based on synthetic lethality observations before complete mechanistic understanding [34]. The robust therapeutic effects demonstrated here provide sufficient rationale for clinical exploration while mechanistic studies proceed in parallel.

Second, our immunodeficient xenograft models cannot assess CD55’s role in tumor-immune interactions. As a complement regulator, CD55 inhibition might enhance complement-mediated tumor clearance, potentially amplifying therapeutic effects beyond what we observed. Evaluating immune contributions will require syngeneic or humanized mouse models in future studies.

Third, although our cell line panel was designed to span MSI, TP53, and BRAF/KRAS heterogeneity, we did not separately profile other modulators of oxaliplatin sensitivity, including CTR1 expression dynamics, ERCC1-driven NER induction, and immunogenic cell death markers (calreticulin, ATP, HMGB1). These factors may contribute to the variable magnitude of CD55-mediated sensitization and warrant systematic dissection in future studies, particularly in immunocompetent models.

Fourth, biomarker implementation will require standardized IHC protocols and prospective validation in clinical cohorts receiving oxaliplatin-based therapy. Although CD55 upregulation was reproducible across cell-line datasets and its clinical relevance was supported in multiple patient cohorts, prospective qPCR/IHC validation in an oxaliplatin-treated patient cohort remains a planned next step. The inverse correlation between CD55 expression and HRD scores further suggests that CD55 could serve as a surrogate marker for HR proficiency, but this hypothesis requires validation in independent datasets with matched genomic and expression date.

Finally, body weight in xenograft studies can be influenced both by drug exposure and by tumor burden, and our two models illustrate this: the body-weight pattern in HT29-OXR appears drug-driven, while in HCT116-OXR a tumor-burden contribution is also present. Dedicated hematology and organ-histology assessment will be needed to characterize tolerability independently of these factors.

## Conclusions

This study identifies CD55 as a stress-induced signaling regulator that supports homologous recombination (HR) capacity and promotes oxaliplatin resistance in colorectal cancer. CD55 is acutely induced by platinum treatment and undergoes intracellular redistribution, including nuclear accumulation, following DNA damage. Functional disruption of CD55 signaling universally sensitizes CRC cells to oxaliplatin, compromises HR capacity, and shifts oxaliplatin–PARP inhibitor interactions from antagonism to synergy (in oxaliplatin-resistant cells) with improved tolerability. Together, these findings position CD55 as both a predictive biomarker for chemotherapy response and a therapeutic vulnerability for inducing a ‘BRCAness-like’ phenotype, providing a functionally grounded strategy to extend PARP inhibitor–based combination approaches beyond BRCA-mutant cancers to HR-proficient, platinum-resistant colorectal cancer.

## Materials and methods

### Cell culture and reagents

Human colorectal cancer cell lines HCT116, HT29, SW480, RKO, and LOVO were obtained from the American Type Culture Collection (ATCC, Manassas, VA, USA). HCT116, HT29, RKO, and SW480 cells were cultured in high-glucose Dulbecco’s Modified Eagle Medium (DMEM; Gibco, Grand Island, NY, USA) supplemented with 10% fetal bovine serum (FBS; Gibco) and 1% penicillin/streptomycin (P/S; Gibco). LOVO cells were cultured in Ham’s F-12 K medium (Gibco) supplemented with 10% FBS and 1% P/S. All cells were maintained at 37 °C in a humidified atmosphere containing 5% CO₂ and routinely subcultured every 72 h with fresh medium changes.

The cell line panel was selected to span the major molecular axes governing oxaliplatin response in CRC, including MSI status (MSI-H: HCT116, RKO, LOVO; MSS: HT29, SW480), TP53 status (wild-type: HCT116, LOVO, RKO; mutant: HT29, SW480), and BRAF/KRAS mutation background. All lines express the copper transporter CTR1; detailed molecular annotations are provided in Table 1.

**Table 1:** Molecular annotations of the colorectal cancer cell line panel

Cell Line	MSI Status	MMR Defect	TP53	BRAF	KRAS
HCT116	MSI-H	MLH1 biallelic mut.	WT	WT	G13D
RKO	MSI-H	MLH1 hypermethyl.	WT	V600E	WT
LOVO	MSI-H	MSH2 biallelic mut.	WT	WT	G13D
HT29	MSS	Proficient	R273H	V600E	WT
SW480	MSS	Proficient	R273H/P309S	WT	G12V

Oxaliplatin (HY-17371) and olaparib (HY-10162) were purchased from MedChemExpress (MCE, Monmouth Junction, NJ, USA). Oxaliplatin was dissolved in basic culture medium for immediate use. Olaparib stock solutions were prepared in dimethyl sulfoxide (DMSO), stored at − 80 °C, and diluted to working concentrations immediately before each experiment.

### Establishment of oxaliplatin-resistant cell lines

Oxaliplatin-resistant cell lines were established through stepwise dose escalation. Logarithmic growth phase HCT116 and HT29 cells were seeded at a density of 5 × 10⁵ cells/mL in culture dishes. After cell attachment, the medium was replaced with medium containing 0.2 µM oxaliplatin. Cells were cultured under this condition for 5–7 days until growth recovery. Subsequently, the oxaliplatin concentration was gradually increased: for HCT116 cells, the concentration was progressively increased to 20 µM; for HT29 cells, to 10 µM, with cells achieving stable growth at each concentration level before escalation. The entire resistance induction process lasted approximately 6 months. The final resistant cell lines, designated HCT116-OXR and HT29-OXR, were capable of stable growth under their respective final oxaliplatin concentrations and were maintained in medium containing oxaliplatin to preserve the resistant phenotype. For all functional experiments described in this study, HCT116-OXR and HT29-OXR cells were transferred to oxaliplatin-free medium and passaged for at least 2 weeks prior to use, to exclude acute drug-persistence effects. Periodic re-assessment of IC50 during routine maintenance confirmed that the resistance phenotype remained stable across this drug-free window.

### Cell viability assay

Cell viability was assessed using the Cell Counting Kit-8 (CCK-8; AR1199, Boster Biological Technology., Ltd., Wuhan, China). Cells were seeded in 96-well plates at a density of 3 × 10³ cells per well and allowed to adhere overnight. Cells were then treated with various concentrations of oxaliplatin or olaparib for 48 hours. CCK-8 reagent (10 µL) was added to each well and incubated for 2 hours at 37°C. Absorbance was measured at 450 nm using a microplate reader (Thermo Fisher Scientific, Waltham, MA, USA). Viability was normalized to vehicle-treated controls (defined as 100%), and absolute IC₅₀ values—defined as the concentration producing 50% of the normalized response—were determined in GraphPad Prism 8.0 (GraphPad Software, San Diego, CA, USA) using the ‘log(agonist) vs. normalized response — variable slope’ model, with the top and bottom plateaus constrained to 100% and 0% and the Hill slope fitted freely. The zero-dose point was excluded from the regression (log(0) undefined). In all cell lines the highest tested concentration reduced viability below 50%; accordingly, all IC₅₀ values were interpolated within the experimental range, and fitted curves were not extrapolated beyond the tested concentrations.

### RNA interference: siRNA-mediated transient knockdown

For CD55 knockdown, four specific siRNAs targeting CD55 (NM_000574.5) were designed and synthesized by GenePharma (Shanghai, China):

-  siCD55-1: 5’-GAAGAGUUCUGCAAUCGUA(dTdT)-3’
-  siCD55-2: 5’-CAAACAGCCUUAUAUCACU(dTdT)-3’
-  siCD55-3: 5’-CGUGACCAUUAUGGAUAUA(dTdT)-3’
-  siCD55-4: 5’-GGUACUACCCGUCUUCUAU(dTdT)-3’
-  Negative control siRNA (siNC): 5’-UUCUCCGAACGUGUCACGU(dTdT)-3’

Transient transfection was performed using siRNA-mate Plus transfection reagent (GenePharma) according to the manufacturer’s protocol. Cells were seeded in 6-well plates at approximately 60% confluence and transfected with siRNAs at a final concentration of 30 nM. Cells were harvested 48 h post-transfection for subsequent analyses.

### RNA interference: stable knockdown

For stable knockdown, the most effective siRNA sequence (siCD55-4: 5’-GGTACTACCCGTCTTCTAT-3’) was used to design short hairpin RNA (shRNA). The shRNA sequence was cloned into the pCLenti-U6-shRNA-CMV-Puro-WPRE lentiviral vector. The negative control shRNA sequence was 5’-TTCTCCGAACGTGTCACGT-3’. Double-stranded DNA fragments were synthesized, annealed, and cloned into BamHI/EcoRI-digested vectors. Recombinant plasmids were verified by sequencing and used for lentivirus production in 293T cells. Target cells were infected with lentiviruses at a multiplicity of infection (MOI) of 10, and stable cell lines were selected with puromycin (2 µg/mL) for 2 weeks.

### CD55 overexpression

For CD55 overexpression, the full-length human CD55 coding sequence was PCR-amplified and cloned into the pcSLenti-CMV-MCS-3×FLAG-PGK-Puro-wpre3 vector, which introduces a 3×FLAG tag at the C-terminus. After sequence verification, recombinant plasmids were co-transfected with lentiviral packaging plasmids into 293T cells. HCT116 cells were infected with CD55-overexpressing lentiviruses using the same protocol as for shRNA, generating stable CD55 overexpression cell lines (CD55-OE) and corresponding empty vector controls (Vector).

### shRNA-resistant CD55 rescue experiment

To exclude potential off-target effects of CD55 shRNA and validate the specificity of CD55-mediated phenotypes, rescue experiments were performed using an shRNA-resistant CD55 expression construct. Silent mutations were introduced into the shCD55 target sequence within the wild-type human CD55 open reading frame (pCMV-CD55(human)-3×HA-Neo; MiaoLingBio, China; Cat# P85216; CD55 reference sequence NM_000574.5) by site-directed mutagenesis, generating the shRNA-resistant construct pCMV-CD55(human)-mut-3×HA-Neo (designated CD55-Res). The mutated target sequence was 5’-GG*A*AC*G*AC*A*CG*G*CT*C*CT*G*T-3’ (substituted nucleotides underlined; the encoded amino acid sequence was preserved). Three plasmids were used in parallel to control for specificity: (i) the empty vector pCMV-MCS-3×HA-Neo (MiaoLingBio, Cat# P13306) as the vector control (Vector); (ii) the wild-type CD55 expression plasmid (Cat# P85216, designated CD55-WT) as an additional control to confirm shRNA-mediated silencing of exogenous CD55 transcripts; and (iii) the shRNA-resistant CD55-Res construct as the rescue condition. All constructs were verified by Sanger sequencing.

HCT116-OXR cells stably expressing shNC or shCD55 were seeded in 24-well plates and transiently transfected with P13306 (Vector), P85216 (CD55-WT), or CD55-Res using Lipofectamine 3000 (Invitrogen, L3000015) according to the manufacturer’s protocol. Briefly, 0.5 µg of plasmid DNA was diluted in 25 µL of Opti-MEM medium, followed by the addition of 1.0 µL of P3000 Enhancer reagent. In parallel, 0.75 µL of Lipofectamine 3000 reagent was diluted in 25 µL of Opti-MEM. The two mixtures were combined, incubated at room temperature for 15 min, and added dropwise to cells at approximately 70% confluence in 24-well plates. To preserve transient transfection efficiency, no G418 antibiotic selection was applied.

For Western blot and qRT-PCR analyses, cells were harvested 48 h post-transfection. For downstream functional assays, transfected cells were trypsinized at 24 h post-transfection and reseeded for CCK-8 cell viability assays (96-well plates) or DR-GFP homologous recombination assays. For DR-GFP assays, the shNC + Vector, shCD55 + Vector, and shCD55 + CD55-Res conditions were assessed; CD55 was stably knocked down by lentiviral shRNA transduction, and the Vector or CD55-Res plasmid was co-transfected with the I-SceI-GR-RFP plasmid at a 1:1 molar ratio. Ectopic CD55 expression was confirmed by Western blot using both anti-CD55 and anti-HA antibodies.

### RNA extraction and quantitative RT-PCR

Total RNA was isolated from cultured cells using TRIzol reagent (Invitrogen, Carlsbad, CA, USA) following the manufacturer’s instructions. RNA concentration and purity were measured with a NanoDrop 2000 spectrophotometer (Thermo Fisher Scientific). One microgram of RNA was reverse transcribed to cDNA using the SureScript First-Strand cDNA Synthesis Kit (GeneCopoeia, Rockville, MD, USA). Quantitative RT-PCR was performed using SYBR Green Master Mix in a 20 µL reaction volume on a Rotor-Gene Q real-time PCR system (Qiagen, Hilden, Germany). Gene expression was normalized to GAPDH and calculated using the 2^−ΔΔCt method.

For qRT-PCR analysis, two CD55 primer pairs were used according to the experimental purpose.

For quantification of CD55 transcripts detected from the shared coding region (used in Fig. 1h and to monitor total CD55 transcript abundance in rescue experiments), the following primers were used:

-  CD55-ORF forward (P1-F): 5’-CAGCACCACCACAAATTGAC-3’
-  CD55-ORF reverse (P1-R): 5’-TGCTCTCCAATCATGGTGAA-3’

This primer pair (P1) amplifies a 112-bp region within the shared CD55 open reading frame (NM_000574.5, nucleotides 765–876), upstream of both the shCD55 target site (NM_000574.5 nt ~ 1175–1193) and the silent substitutions introduced into CD55-Res. Accordingly, P1 detects endogenous CD55 transcripts as well as both CD55-WT- and CD55-Res-derived transcripts, and was used as a readout of total CD55 transcript abundance in rescue experiments.

To distinguish endogenous non-plasmid CD55 transcripts from ectopic rescue transcripts, an additional primer pair was used:

-  CD55 forward (P2-F): 5’-TCTATCTGGTTCTCGTCCTGTCAC-3’
-  CD55 reverse (P2-R): 5’-TTGGGAGGCTGAAGTGGAAGG-3’

This primer pair (P2) amplifies a 110-bp region within an alternate 3’ CD55 sequence present in endogenous transcripts but absent from the rescue expression plasmid, which contains only the canonical CD55 open reading frame (NM_000574.5, nt 89–1234). Therefore, P2 does not amplify plasmid-derived rescue transcripts and was used to monitor endogenous CD55 transcript depletion independently of ectopic CD55 re-expression.

Combined use of P1 (total CD55; endogenous + ectopic) and P2 (endogenous alternate-3’ transcripts only) allowed the rescue experiment to be read out along two complementary axes: P2 confirmed that shCD55 effectively depleted endogenous CD55 transcripts in all shCD55 groups regardless of which plasmid was co-transfected, while P1 demonstrated restoration of total CD55 transcript abundance specifically in the shCD55 + CD55-Res group, consistent with successful shRNA-resistant rescue.

For GAPDH normalization:

- GAPDH forward: 5’-CTCCTCCTGTTCGACAGTCAGC-3’
- GAPDH reverse: 5’-CCCAATACGACCAAATCCGTT-3’

### Western blot analysis

Total proteins were extracted using RIPA lysis buffer (Beyotime, Shanghai, China) containing protease inhibitor cocktail. Protein concentrations were determined using the BCA Protein Assay Kit (Beyotime). Equal amounts of protein (20–30 µg per lane) were subjected to SDS-PAGE electrophoresis and transferred to polyvinylidene difluoride (PVDF) membranes (Millipore, Burlington, MA, USA). Membranes were blocked with 5% non-fat milk in Tris-buffered saline with 0.1% Tween-20 (TBST) at room temperature for 1 h, then incubated with primary antibodies overnight at 4 °C:

-  Anti-CD55 (rabbit polyclonal, 1:2000, Invitrogen, #PA5-80438)
-  Anti-CD55 (rabbit polyclonal,1:2000, BOSTER, #PB9487)
-  Anti-CD55 (mouse monoclonal, 1:200, Santa Cruz, #sc-51733)
-  Anti-BCL-2 (1:2000, Proteintech, #12789-1-AP)
-  Anti-BAX (1:1000, BOSTER, #ba0315-2)
-  Anti-BRCA1 (1:2000, Proteintech, #22362-1-AP)
-  Anti-CtIP/RBBP8 (1:1000, Activemotif, #61142)
-  Anti-RAD51 (1:1000, Proteintech, #14961-1-AP)
-  Anti-γ-H2AX (1:5000, Proteintech, #83307-2-RR)
-  Anti-Histone H3 (1:5000, Abcam, #ab1791)
-  Anti-α-Tubulin (1:5000, Proteintech, #11224-1-AP)
-  Anti-β-actin (1:5000, Abcam, #ab8226)
-  Anti-GAPDH (1:50000, Proteintech, #60004-1-lg)
-  Anti-ATM (1:2000, Proteintech, #27156-1-AP)
-  Anti-Phospho-ATM(Ser1981) (1:1000, Beyotime, # AA866)
-  Anti-HA (1:10000, Proteintech, #66006-2-Ig)

After washing with TBST, membranes were incubated with HRP-conjugated secondary antibodies (1:10000, Proteintech) for 1 h at room temperature. Protein bands were visualized using enhanced chemiluminescence (ECL) reagents (BOSTER) and detected with a ChemiDoc XRS+ imaging system (Bio-Rad, Hercules, CA, USA). Protein expression was quantified using ImageLab software (Bio-Rad) with GAPDH, β-actin, or α-tubulin as loading controls.

### Immunofluorescence microscopy

Cells were cultured on sterile chamber slides (Thermo Fisher Scientific) for 24 h and, where indicated, treated with oxaliplatin as specified for each experiment below. Cells were fixed with 4% paraformaldehyde for 15 min at room temperature, permeabilized with 0.1% Triton X-100 for 5 min, and blocked with 5% bovine serum albumin (BSA) for 1 h. Primary antibodies were applied overnight at 4 °C; cells were then washed with PBS and incubated for 1 h at room temperature in the dark with Alexa Fluor 488-conjugated anti-rabbit IgG and Alexa Fluor 594-conjugated anti-mouse IgG secondary antibodies (1:1000, Invitrogen). Nuclei were counterstained with DAPI-containing anti-fade mounting medium (Beyotime P0131). Images were acquired on a Nikon C2 laser-scanning confocal microscope (Nikon Corporation, Tokyo, Japan) under identical exposure settings for all conditions within an experiment and quantified in ImageJ (NIH). Unless otherwise specified, quantification was performed across five random fields per condition from three independent experiments.

Primary antibodies:

- Anti-CD55 (rabbit polyclonal, 1:400, BOSTER #PB9487)
- Anti-CD55 (mouse monoclonal, 1:40, Santa Cruz #sc-51733)
- Anti-β-tubulin (mouse, 1:400, Abbkine #a1030)
- Anti-γ-H2AX (rabbit, 1:400, Proteintech #83307-2-RR)
- Anti-RAD51 (mouse monoclonal, 1:400, Proteintech #67024-1-Ig)

(i) CD55 subcellular localization. HCT116 and HCT116-OXR cells were left untreated or treated with oxaliplatin (2 µM for HCT116, 10 µM for HCT116-OXR) for 24 h, then co-stained for CD55 (#PB9487; Alexa 488) and β-tubulin (#a1030; Alexa 594). CD55 intensity was normalized to β-tubulin as an internal reference; 50–100 cells per condition were analyzed.
(ii) Antibody-specificity validation. shNC and shCD55 HCT116-OXR cells were treated with vehicle or 10 µM oxaliplatin (24 h) and co-stained for CD55 (mouse monoclonal, #sc-51733; Alexa 594) and γ-H2AX (#83307-2-RR; Alexa 488). Nuclear CD55 intensity and γ-H2AX foci were quantified across ≥ 100 cells per condition.
(iii) RAD51/γ-H2AX dual staining. HCT116-OXR shNC and shCD55 cells were pulsed with oxaliplatin (10 µM, 1 h), allowed to recover for 0, 1, 4 and 8 h, and co-stained for RAD51 (#67024-1-Ig; Alexa 594), γ-H2AX (#83307-2-RR; Alexa 488) and DAPI. Foci were scored by an automated pipeline (DAPI segmentation → background suppression → Laplacian-of-Gaussian spot detection → per-nucleus adaptive thresholding; border and low-confidence nuclei excluded), yielding 1,370 nuclei across three independent experiments.

### Colony formation assay

Cells were seeded in 6-well plates at a density of 500–1000 cells per well and treated with indicated concentrations of drugs. For HCT116 parental cells, treatment concentrations included 0.5-2 µM oxaliplatin, 0.5 µM olaparib, or combinations thereof. For HCT116-OXR resistant cells, higher concentrations (2–5 µM oxaliplatin, 2 µM olaparib) were used. After 10–14 days of culture with medium changed every 3 days, colonies were fixed with methanol for 15 min and stained with 0.1% crystal violet for 30 min. Colonies containing > 50 cells were counted manually. Relative colony formation rate was calculated as (number of colonies in treatment group/number of colonies in control group) × 100%, with the control group normalized to 100%. For CD55 knockdown experiments, shNC or siNC served as the control group.

### Transwell migration and invasion assays

For migration assays, 2 × 10⁴ cells in serum-free medium were seeded in the upper chamber of 24-well Transwell plates (8-µm pore size, Corning, Corning, NY, USA). For invasion assays, chambers were pre-coated with Matrigel (BD Biosciences, San Jose, CA, USA; diluted 1:8 in serum-free medium) and incubated at 37 °C for 1 h before cell seeding. Complete medium containing 20% FBS was added to the lower chamber as a chemoattractant. After incubation for 24 h (migration) or 36 h (invasion), non-migrating cells on the upper surface were removed with a cotton swab. Migrated or invaded cells were fixed with 4% paraformaldehyde for 15 min, stained with 0.1% crystal violet for 30 min, and counted under a microscope. Five random fields were counted at 200× magnification per well.

### Flow cytometry analysis: apoptosis detection

Apoptosis was assessed using the Annexin V-FITC/PI Apoptosis Detection Kit (BD Biosciences) according to the manufacturer’s instructions. Following oxaliplatin treatment (4 µM for 48 h), cells were harvested, washed with PBS, and stained according to manufacturer’s instructions. Samples were analyzed within 1 h using a BD FACSCalibur flow cytometer (BD Biosciences). Data were analyzed with FlowJo software (TreeStar, Ashland, OR, USA) to determine early apoptotic (Annexin V⁺/PI⁻) and late apoptotic (Annexin V⁺/PI⁺) cell populations.

### DR-GFP homologous recombination assay

HR efficiency was measured using the DR-GFP reporter system. Stable DR-GFP cell lines (HCT116-DR, HCT116-OXR-DR) were established by transfecting cells with pDR-GFP using Lipofectamine 2000 (Invitrogen), selecting with puromycin (2 µg/mL), and isolating single-cell clones by limiting dilution. Multiple clones per line were screened, and clones meeting all three criteria were selected: basal GFP < 1% in the uninduced state, single-copy DR-GFP integration confirmed by genomic qPCR, and reproducible I-SceI–induced GFP signal.

Reporter copy number was assessed by genomic qPCR using two independent primer sets within the GFP cassette (GFP-95: forward 5′-AAGGGCATCGACTTCAAGG-3′, reverse 5′-TGCTTGTCGGCCATGATATAG-3′; GFP-55: forward 5′-AGTCCGCCCTGAGCAAAGA-3′, reverse 5′-TCCAGCAGGACCATGTGATC-3′) normalized to the single-copy reference gene *RPP30* (forward 5′-GATTTGGACCTGCGAGCG-3′, reverse 5′-GCGGCTGTCTCCACAAGT-3′). All primer sets demonstrated amplification efficiencies between 96% and 102%. Relative copy number was calculated using the ΔΔCt method, with parental HCT116 or HCT116-OXR cells (lacking the reporter) included as specificity controls. Both selected clones were confirmed to carry single-copy DR-GFP integration.

For HR assays, DR-GFP cells were transfected with siCD55 or siNC for 48 h, then with I-SceI-GR-RFP plasmid. Twenty-four hours later, triamcinolone acetonide (0.1 µM) was added; cells were harvested 24 h post-induction for flow cytometry. Gating hierarchy: debris exclusion (FSC-A/SSC-A) → doublet exclusion (FSC-H/FSC-A) → RFP⁺ gate → GFP⁺ gate, with both thresholds defined against no-plasmid controls (cells transfected with siRNA only) to ensure a HR-naïve reference population. No-induction controls, and copy-number verification are shown in Supplementary Figure S4a. All experiments were performed in three biological replicates.

### Cell cycle analysis

HCT116-OXR cells stably expressing shNC or shCD55 were left untreated or treated with oxaliplatin (5 or 25 µM) for 24 h. Cells were harvested, fixed in ice-cold 70% ethanol overnight at − 20 °C, washed in PBS, and stained with propidium iodide (50 µg/mL) containing RNase A (100 µg/mL) for 30 min at 37 °C in the dark. At least 10,000 events per sample were acquired on a BD FACSCalibur flow cytometer (BD Biosciences) and analysed using ModFit software. Three independent experiments were performed.

### Alkaline comet assay

DNA damage and repair kinetics were assessed using the Alkaline Comet Assay Kit (KeyGEN, KGA1302-20, Nanjing, China) according to the manufacturer’s instructions. Cells were treated with oxaliplatin (2 µM for HCT116, 10 µM for HCT116-OXR) for 1 h, washed with PBS, and allowed to recover in complete medium for 0, 1, 8, or 24 h.

Cells (approximately 1 × 10⁴) were mixed with 0.7% low melting point agarose and spread onto slides pre-coated with 1% normal melting point agarose. After solidification at 4 °C, cells were lysed in alkaline lysis buffer at 4 °C for 1 h in the dark. DNA was unwound in alkaline electrophoresis buffer (1 mM EDTA, 300 mM NaOH, pH > 13) for 30 min at room temperature, followed by electrophoresis at 20 V (approximately 1 V/cm) for 20 min at 4 °C in the dark. Slides were neutralized three times with 0.4 M Tris-HCl (pH 7.5) for 10 min each, stained with propidium iodide (PI), and analyzed using fluorescence microscopy (515–560 nm excitation). DNA damage was quantified by measuring Olive Tail Moment (OTM) values using OpenComet software (version 1.3.1). At least 100 cells per condition were analyzed from three independent experiments.

### Nuclear-cytoplasmic fractionation

HCT116 parental and HCT116-OXR resistant cells were seeded, allowed to attach, and treated with 2 µM (HCT116) or 10 µM (HCT116-OXR) oxaliplatin for 24 h; control groups received an equal volume of medium. Nuclear and cytoplasmic proteins were extracted using the Nuclear-Cytosol Extraction Kit (Applygen, P1200, Beijing, China) according to the manufacturer’s instructions. Briefly, cells were lysed with cytoplasmic extraction buffers to isolate cytoplasmic proteins, and the remaining nuclei were lysed with nuclear extraction buffer to obtain nuclear proteins. Fraction purity was confirmed by Western blot analysis of α-tubulin (cytoplasmic marker) and histone H3 (nuclear marker).

### Drug combination analysis

Drug interactions between oxaliplatin and olaparib were evaluated using the combination index (CI) method based on the Chou-Talalay principle, calculated with CompuSyn software (ComboSyn Inc., Paramus, NJ, USA). Oxaliplatin was dissolved in culture medium (1 mM stock solution), and olaparib was dissolved in DMSO (40 mM stock solution) and diluted to working concentrations with < 0.1% final DMSO concentration.

For combination treatments, drugs were mixed at a constant 1:1 molar ratio across logarithmic concentration gradients ranging from 0.5 to 200 µM. A fixed-ratio design was used to enable CI calculation across a broad effect range in CompuSyn; dose ranges were chosen based on single-agent IC₅₀ values in each cell context. Cells were seeded in 96-well plates and treated with single agents or combinations for 48 h. Cell viability was assessed by CCK-8 assay. Dose-response data were entered into CompuSyn software to calculate CI values at multiple effect levels (Fa, fraction affected).

CI values were interpreted as: CI < 1 (synergistic), CI = 1 (additive), and CI > 1 (antagonistic). Isobologram analysis was performed to validate synergistic interactions, where data points falling below the line of additivity indicate synergism. All experiments were performed in triplicate and repeated three times independently.

### Animal studies

All animal experiments were approved by the Institutional Animal Care and Use Committee. Female BALB/c nude mice (6 weeks old, ~ 18 g) were obtained from Beijing HFK Bioscience Co., Ltd. (Beijing, China). To validate findings across genetically distinct backgrounds, two independent oxaliplatin-resistant models were used: HCT116-OXR (microsatellite-unstable, p53-wild-type) and HT29-OXR (microsatellite-stable, p53-mutant). Cells (5 × 10⁶) with stable CD55 knockdown (shCD55) or negative control (shNC) were mixed with Matrigel (1:1) and injected subcutaneously into the right flank. Once tumors became palpable, mice were randomized into six treatment groups: shNC + Vehicle (saline), shNC + Oxaliplatin (5 mg/kg), shNC + Combination; shCD55 + Vehicle, shCD55 + Oxaliplatin (5 mg/kg), and shCD55 + Combination. The HT29-OXR cohort comprised *n* = 6 per group (36 mice) and the HCT116-OXR cohort *n* = 4 per group (24 mice). Caliper measurements and tumor weighing were performed by an operator blinded to group assignment. All treatments were administered via intraperitoneal (i.p.) injection every 3 days.

Oxaliplatin was freshly prepared in sterile water at 0.5 mg/mL. Olaparib was dissolved in DMSO and diluted in 20% sulfobutyl-β-cyclodextrin (SBE-β-CD) to 2 mg/mL final concentration. Treatment was initiated when tumors became palpable and continued for 18 days.

Tumor volumes were measured every 3 days using digital calipers and calculated using the formula: V = (a × b²)/2, where a is the longest diameter and b is the shortest perpendicular diameter. Body weights were monitored every 3 days as indicators of systemic toxicity. Mice were euthanized by cervical dislocation when control tumors reached approximately 1000 mm³ or after 18 days of treatment. Tumors were harvested, photographed, and weighed. Portions were fixed in 4% paraformaldehyde for 24 h and embedded in paraffin for histological analysis. The maximal tumor size permitted by the Ethics Committee of The Second Hospital of Hebei Medical University is 1500 mm³, and this limit was not exceeded in any experiments.

### Histological and immunohistochemical analysis

Paraffin-embedded tumor tissues were sectioned at 4–5 μm thickness. Hematoxylin and eosin (H&E) staining was performed to assess tumor morphology. Immunohistochemistry was performed using antibodies against CD55 (1:200), Ki-67 (1:200, Abcam ab15580),BRCA1 (1:200, Proteintech, #22362-1-AP), Cleaved Caspase3 (1:200, Selleck, #F0135) and γ-H2AX (1:200, Cell Signaling Technology #9718). Sections were deparaffinized, rehydrated, subjected to antigen retrieval in citrate buffer (pH 6.0), blocked with 5% BSA, and incubated with primary antibodies overnight at 4 °C. After washing, sections were incubated with HRP-conjugated secondary antibodies and developed with DAB substrate (Vector Laboratories). Images were captured at 40× and 400× magnification using an Olympus BX53 microscope.

### RNA sequencing and bioinformatics analysis

Total RNA was extracted from HCT116 and HCT116-OXR cells (*n* = 3 per group) and subjected to RNA sequencing on the Illumina NovaSeq 6000 platform (paired-end 150 bp reads). Quality control metrics confirmed high-quality sequencing data with Q20 > 97.7% and unique mapping rates > 95.1% (Figure S1d-g). Clean reads were aligned to the human reference genome (GRCh38.p13) using STAR aligner (version 2.7.7a), and differential expression analysis was performed using DESeq2 (version 1.44.0) with |log₂ fold change| > 0.5 and false discovery rate (FDR) < 0.05 as significance criteria. Gene Ontology (GO) and Kyoto Encyclopedia of Genes and Genomes (KEGG) (Figure S1f) pathway enrichment analyses were performed using clusterProfiler (version 4.4.0).

Two independent GEO datasets (GSE119603 and GSE138647) containing RNA-seq data from HCT116 and HCT116-OXR cells were downloaded and analyzed using the same pipeline. Differential expression analysis was performed using DESeq2 (version 1.44.0) with identical significance criteria. Benjamini-Hochberg method was used for multiple testing correction. Consistently upregulated genes across all three datasets were identified using Venn diagram analysis. Concordant genes were ranked by mean log2 fold change across the three datasets (with Fisher’s combined adjusted p as a secondary metric), and the up- and down-regulated sets were subjected to GO and KEGG over-representation analysis (clusterProfiler, Benjamini-Hochberg correction).

### Pan-cancer HRD score analysis

TCGA Pan-Cancer (PANCAN) normalized gene expression data (10,535 samples, 60,499 genes) were obtained from the UCSC Xena database. CD55 gene expression (ENSG00000196352) was extracted and log₂(x + 0.001) transformed. Sample types were limited to primary tumors and blood-derived cancers. HRD scores and LOH scores for corresponding samples were obtained from published data [35]. Cancer types with fewer than 3 samples were excluded, resulting in 37 cancer types for analysis.

Spearman rank correlation analysis was performed to assess the relationship between CD55 expression and HRD/LOH scores for each cancer type. Multiple testing correction was performed using the Benjamini-Hochberg method to control FDR. For COAD samples (*n* = 283 for HRD, *n* = 275 for LOH), separate correlation analyses were performed. All analyses were conducted using the Sangerbox online platform and R software (version 4.4.1) with ggplot2 and ggpubr packages.

### Clinical data analysis: TCGA-COAD analysis

CD55 expression data and clinical information from TCGA-COAD dataset (481 tumor samples, 41 normal samples) were obtained through TCGAbiolinks. Expression differences between tumor and normal tissues were compared using Wilcoxon rank-sum test. Associations between CD55 expression and clinical variables (stage, age, gender) were assessed using Kruskal-Wallis test or Wilcoxon test as appropriate.

### Clinical data analysis: GEO dataset analysis

GEO datasets GSE17536 [36] (*n* = 145 colorectal cancer patients with clinical follow-up) and GSE72970 [37] (*n* = 31 patients treated with FOLFOX chemotherapy) were downloaded from the GEO database. For GSE17536, CD55 expression was extracted (probe 201925_s_at), and patients were stratified into high and low expression groups based on median expression. Associations between CD55 expression and disease recurrence were assessed using Wilcoxon test.

Survival analysis was performed using the Kaplan-Meier method with log-rank test for disease-free survival (DFS) and overall survival (OS). Multivariate Cox proportional hazards regression models were constructed including CD55 expression, AJCC stage, age, gender, and other relevant clinical variables. Hazard ratios (HR) with 95% confidence intervals (CI) were calculated. All survival analyses were performed using the survival (version 3.7-0) and survminer (version 0.5.0) packages in R.

### Statistical analysis

All in vitro experiments were performed in ≥ 3 independent biological replicates (*n* = 3); data are mean ± SEM unless stated, with the exact n given in each figure legend. Tests were two-tailed and *P* < 0.05 was considered significant. Two-group comparisons used the unpaired Student’s t-test unless specified otherwise.

Because IC₅₀ values are log-normally distributed, IC₅₀ comparisons were made on log₁₀(IC₅₀) by Welch’s t-test; absolute IC₅₀ values were interpolated within range from variable-slope fits with plateaus constrained to 100%/0%, and fold-changes are reported as geometric means. Western blot and qRT-PCR ratios were normalized to the paired control, log₂-transformed, and tested against 0 by a one-sample t-test (*n* = 3); dose- and time-dependent CD55 induction was analyzed by repeated-measures one-way ANOVA (Geisser–Greenhouse) with linear-trend and Dunnett’s tests. DR-GFP knockdown was compared by Student’s t-test and the rescue series by one-way ANOVA with Tukey’s HSD.

For single-cell comet and immunofluorescence assays, scored by automated operator-independent pipelines in Python, the biological replicate was the unit of analysis (*n* = 3; γ-H2AX intensity *N* = 4): γ-H2AX intensity, RAD51/γ-H2AX foci, p-ATM/ATM and combination γ-H2AX were analyzed by two-way ANOVA with Šídák correction, and comet Olive Tail Moment by Welch’s t-test at each recovery time (24 h pre-specified endpoint). Oxaliplatin–olaparib combination indices (Chou–Talalay; fixed ratios; Fa > 0.1) were assessed by Welch’s t-test per ratio, two-way ANOVA on log-CI, and a pooled Mann–Whitney U test.

In vivo tumor growth was modeled by linear mixed effects (mouse as random effect; Šídák-corrected endpoint contrasts), cross-validated in Prism/R with Shapiro–Wilk and Kolmogorov–Smirnov normality checks; endpoint tumor volume and weight by two-way ANOVA, and body weight by repeated-measures/one-way ANOVA with Tukey’s (full output in Supplementary Table S4). Transcriptomic differential expression used DESeq2 and clusterProfiler (Benjamini–Hochberg, FDR < 0.05); CD55–HRD/LOH correlations by Spearman; clinical comparisons by Wilcoxon, Kruskal–Wallis or limma; and survival by Kaplan–Meier/log-rank with multivariate Cox regression. Analyses used GraphPad Prism 8.0 and R 4.4.1. *P < 0.05, **P < 0.01, ***P < 0.001, ****P < 0.0001; ns, not significant.

## Supplementary Information

Supplementary Material 1.

Supplementary Material 2.

Supplementary Material 3.

Supplementary Material 4.

Supplementary Material 5.

## Data Availability

The RNA-sequencing data generated in this study have been deposited in the Gene Expression Omnibus (GEO) repository under accession number GSE314768. Publicly available datasets analyzed in this study were obtained from The Cancer Genome Atlas (TCGA; https://portal.gdc.cancer.gov/) and GEO datasets GSE119603, GSE138647, GSE17536, and GSE72970.
