# Supplementary material for: Stress-induced CD55 supports homologous recombination capacity and drives oxaliplatin resistance in colorectal cancer
Source: Cell Commun Signal. 2026 Jul 10;24:532. doi: 10.1186/s12964-026-03047-z (PMC13637197; doi:10.1186/s12964-026-03047-z)
Supplement: Supplementary file 1 [file 12964_2026_3047_MOESM1_ESM.pdf]

Figure S1. Supplementary characterization of oxaliplatin-resistant models and CD55 expression.

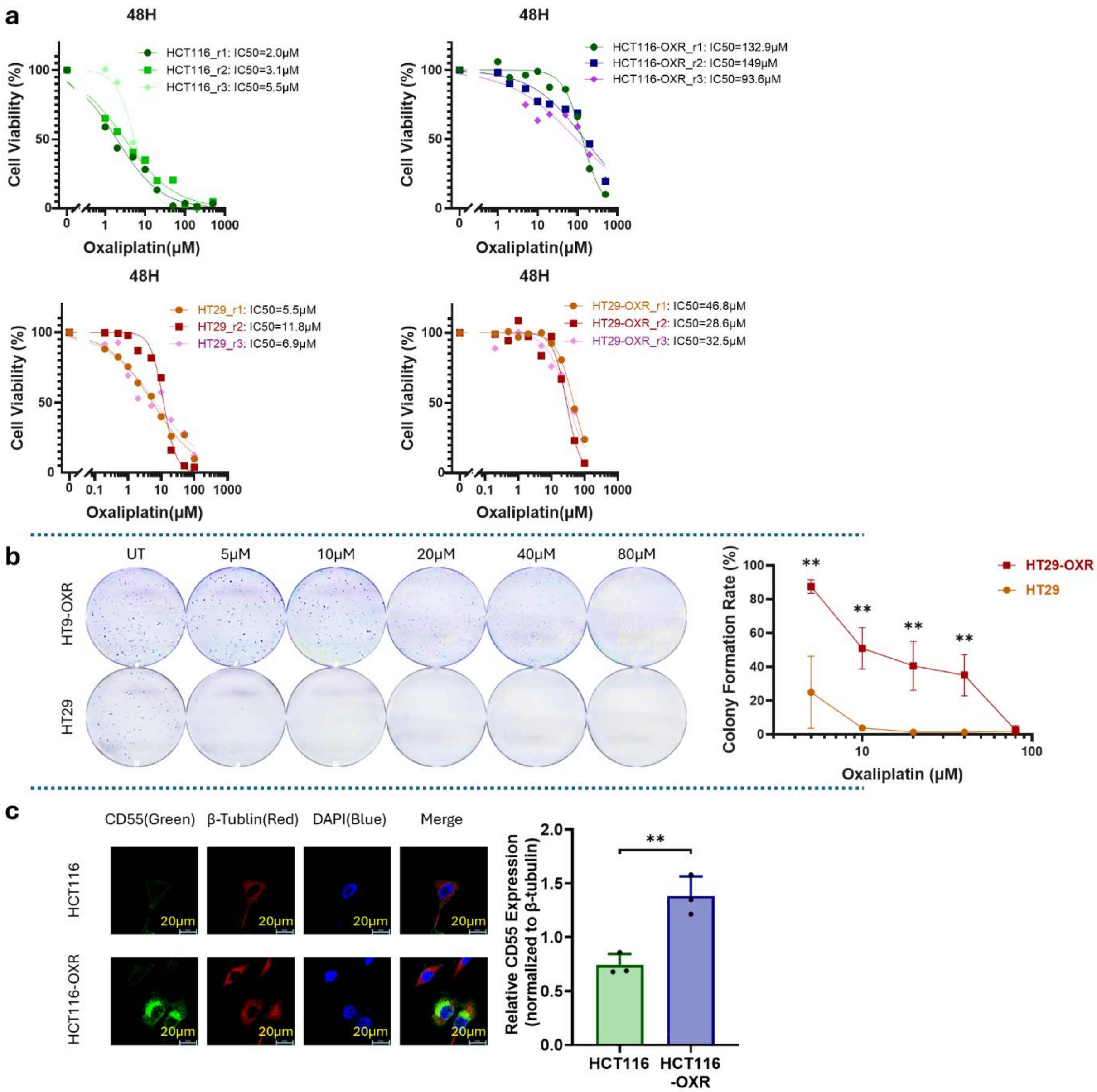

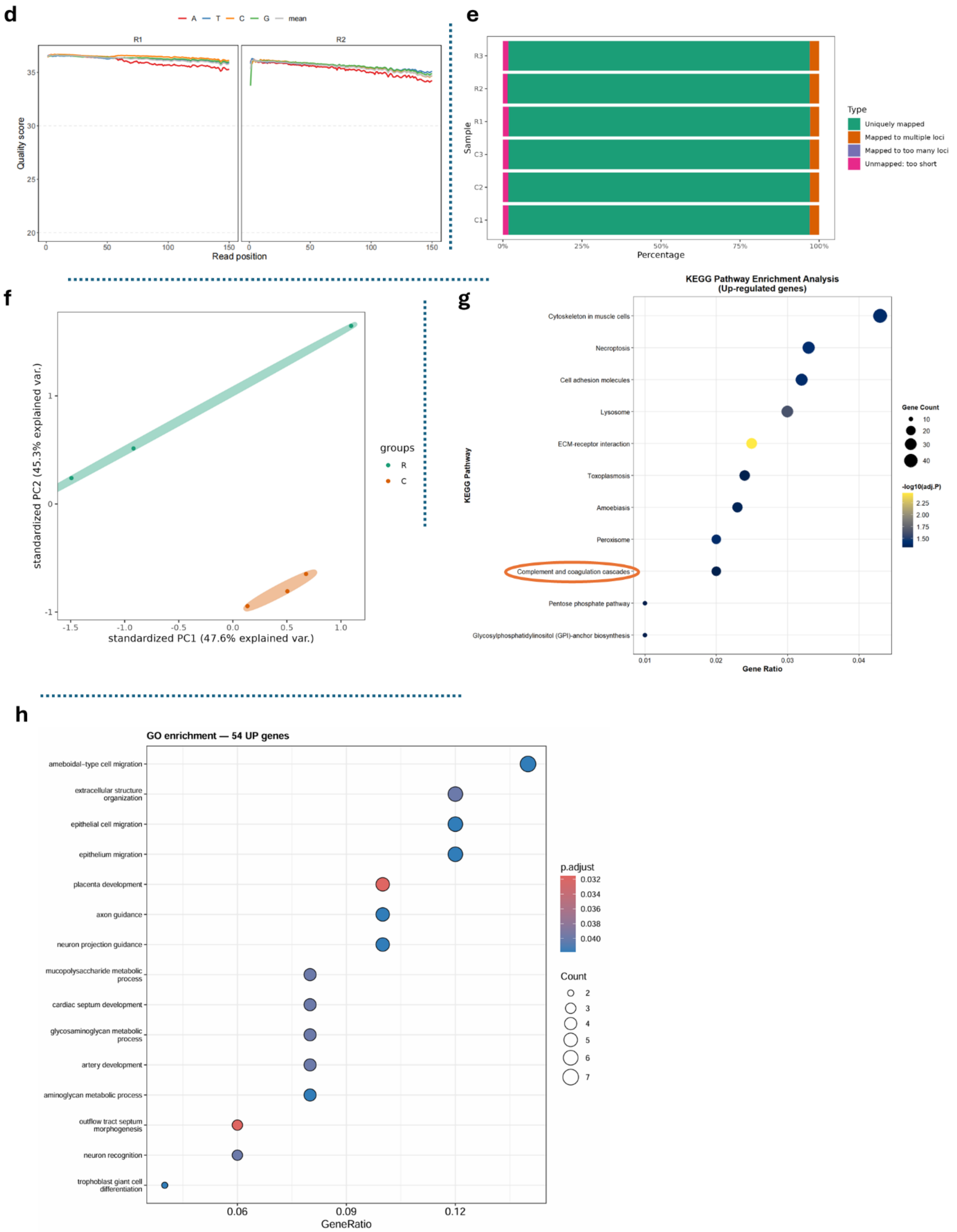

(a) Per-replicate oxaliplatin dose–response curves for the parental and resistant cell lines, with each biological replicate fitted individually. Viability was normalized to vehicle-treated controls (100%) and fitted with a variable-slope (four-

parameter) model with top/bottom constrained to 100%/0%; curves are plotted only across the tested concentration range, and all absolute IC_{50} values were interpolated within range without extrapolation. Per-replicate fit parameters (absolute IC_{50}, Hill slope, 95% CI, R^2, n) are provided in Supplementary Table S2. **(b)** Colony formation assays comparing HT29 and HT29-OXR cells under oxaliplatin treatment, showing enhanced clonogenic survival in resistant cells. **(c)** Immunofluorescence of CD55 subcellular localization: CD55 (green) showed increased intensity in HCT116-OXR cells, with a subpopulation displaying cytoplasmic/perinuclear signal; β-tubulin (red), cytoskeletal marker; DAPI (blue), nuclei. CD55 intensity was increased 1.86-fold. Scale bar: 20 μm; ⩾50 cells per group across five fields from three independent experiments. **(d)** RNA-seq quality-control metrics (Q20 > 97.7%; unique mapping rate > 95.1%). **(e)** Principal component analysis showing transcriptional separation between HCT116 and HCT116-OXR cells (n = 3/group). **(f)** Alignment statistics for mapping to the human reference genome (unique mapping rate > 95.1%; see panel d. **(g)** KEGG pathway enrichment of the up-regulated differentially expressed genes from the HCT116 vs. HCT116-OXR RNA-seq comparison, revealing significant enrichment in complement and coagulation cascades (including DAF/CD55), ECM-receptor interactions, and necroptosis pathways. **(h)** GO over-representation analysis of the 54 concordant up-regulated genes, showing significant enrichment in cell-migration, extracellular-structure-organization and glycosaminoglycan/mucopolysaccharide-metabolism processes; dot size indicates gene count and color the adjusted p-value (top enriched biological processes shown). GO and KEGG over-representation analyses were performed with clusterProfiler using Benjamini–Hochberg correction (FDR < 0.05); for the three-dataset concordant gene sets, KEGG returned no significant pathway for the 54 up-regulated genes and GO returned no significant term for the 179 down-regulated genes. Quantitative wet-lab data in (b, c) are mean ± SEM from three independent experiments (two-tailed Student's t-test); $*P < 0.05$, $**P < 0.01$; ns, not significant.

Figure S2. Supplementary validation of CD55 knockdown and functional effects.

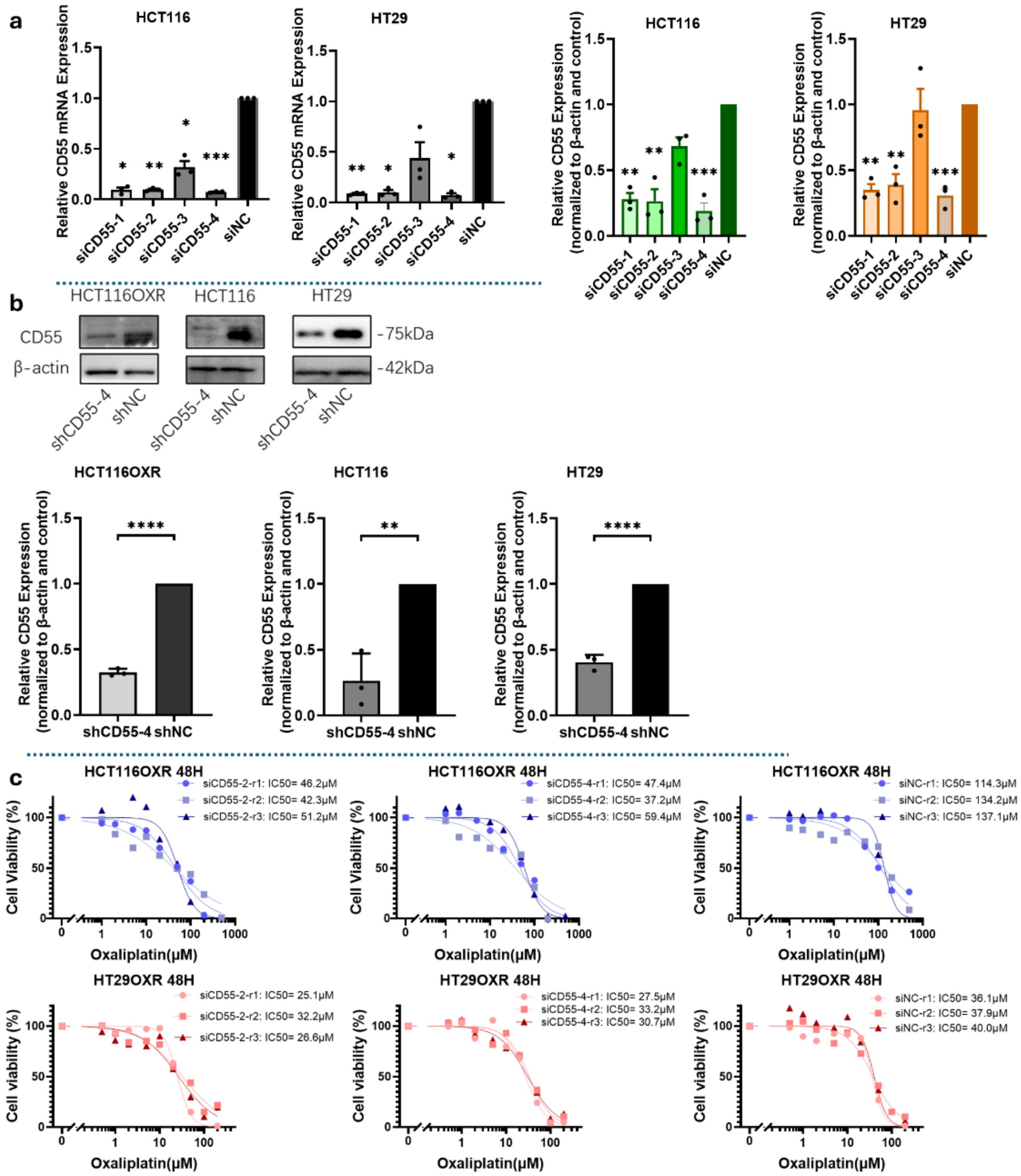

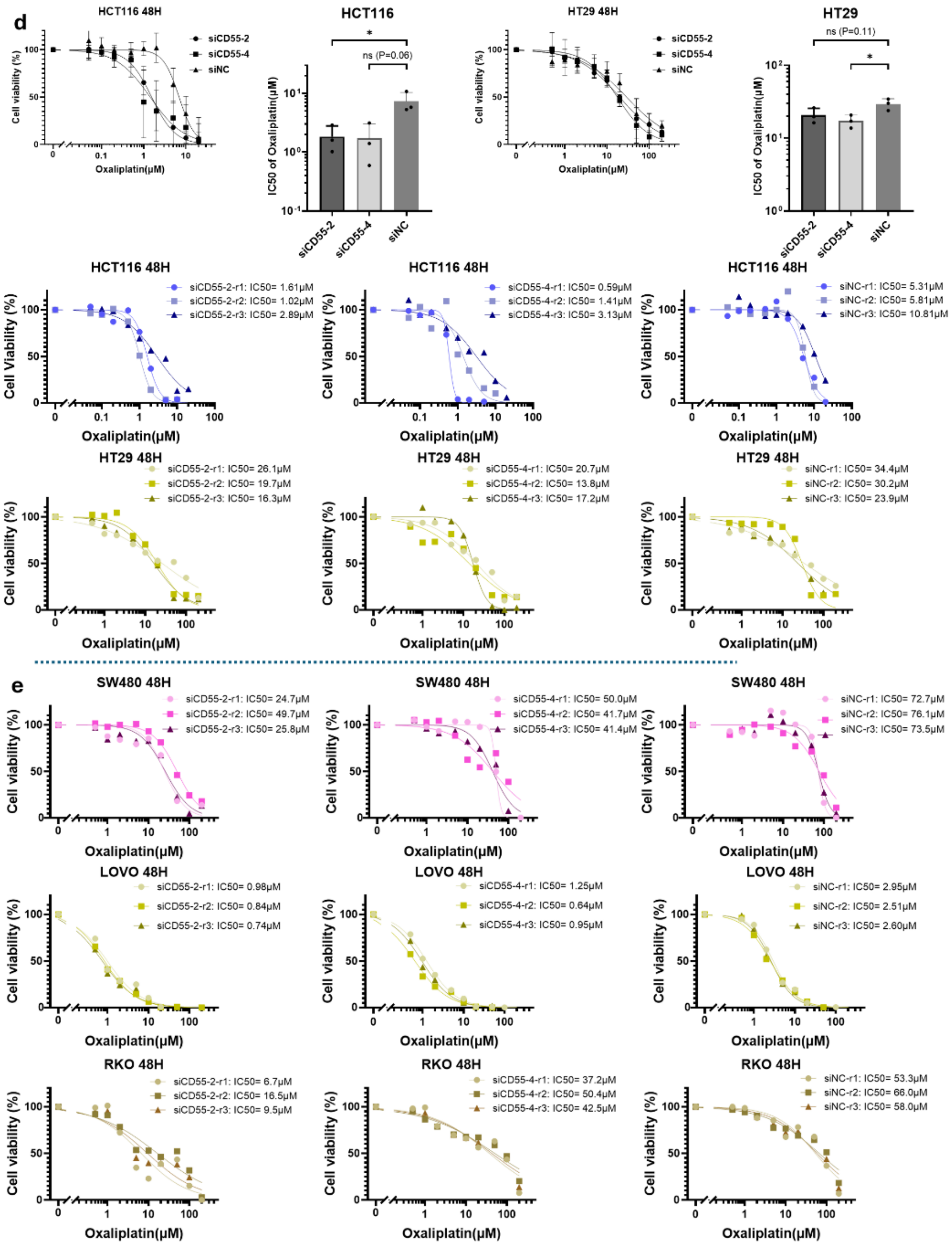
d
HCT116 48H
siCD55-2
siCD55-4
siNC
Cell viability (%)
Oxaliplatin(μM)
HCT116
IC50 of Oxaliplatin(μM)
*
ns (P=0.06)
siCD55-2
siCD55-4
siNC
HT29 48H
HT29
ns (P=0.11)
HCT116 48H
siCD55-2-r1: IC50= 1.61μM
siCD55-2-r2: IC50= 1.02μM
siCD55-2-r3: IC50= 2.89μM
siCD55-4-r1: IC50= 0.59μM
siCD55-4-r2: IC50= 1.41μM
siCD55-4-r3: IC50= 3.13μM
siNC-r1: IC50= 5.31μM
siNC-r2: IC50= 5.81μM
siNC-r3: IC50= 10.81μM
Cell Viability (%)
HT29 48H
siCD55-2-r1: IC50= 26.1μM
siCD55-2-r2: IC50= 19.7μM
siCD55-2-r3: IC50= 16.3μM
siCD55-4-r1: IC50= 20.7μM
siCD55-4-r2: IC50= 13.8μM
siCD55-4-r3: IC50= 17.2μM
siNC-r1: IC50= 34.4μM
siNC-r2: IC50= 30.2μM
siNC-r3: IC50= 23.9μM
e
SW480 48H
siCD55-2-r1: IC50= 24.7μM
siCD55-2-r2: IC50= 49.7μM
siCD55-2-r3: IC50= 25.8μM
siCD55-4-r1: IC50= 50.0μM
siCD55-4-r2: IC50= 41.7μM
siCD55-4-r3: IC50= 41.4μM
siNC-r1: IC50= 72.7μM
siNC-r2: IC50= 76.1μM
siNC-r3: IC50= 73.5μM
LOVO 48H
siCD55-2-r1: IC50= 0.98μM
siCD55-2-r2: IC50= 0.84μM
siCD55-2-r3: IC50= 0.74μM
siCD55-4-r1: IC50= 1.25μM
siCD55-4-r2: IC50= 0.64μM
siCD55-4-r3: IC50= 0.95μM
siNC-r1: IC50= 2.95μM
siNC-r2: IC50= 2.51μM
siNC-r3: IC50= 2.60μM
RKO 48H
siCD55-2-r1: IC50= 6.7μM
siCD55-2-r2: IC50= 16.5μM
siCD55-2-r3: IC50= 9.5μM
siCD55-4-r1: IC50= 37.2μM
siCD55-4-r2: IC50= 50.4μM
siCD55-4-r3: IC50= 42.5μM
siNC-r1: IC50= 53.3μM
siNC-r2: IC50= 66.0μM
siNC-r3: IC50= 58.0μM
Cell viability (%)
Oxaliplatin(μM)

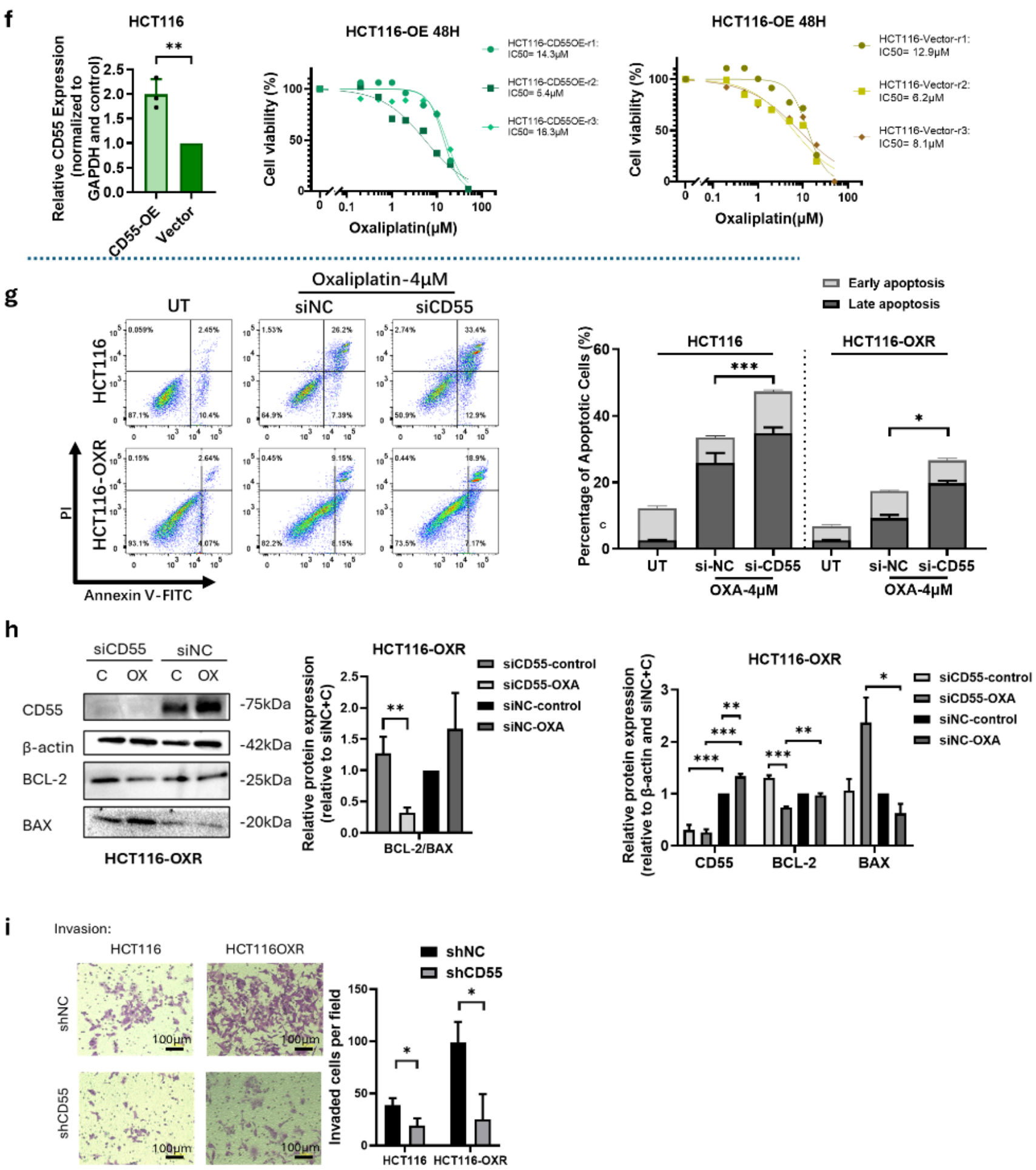

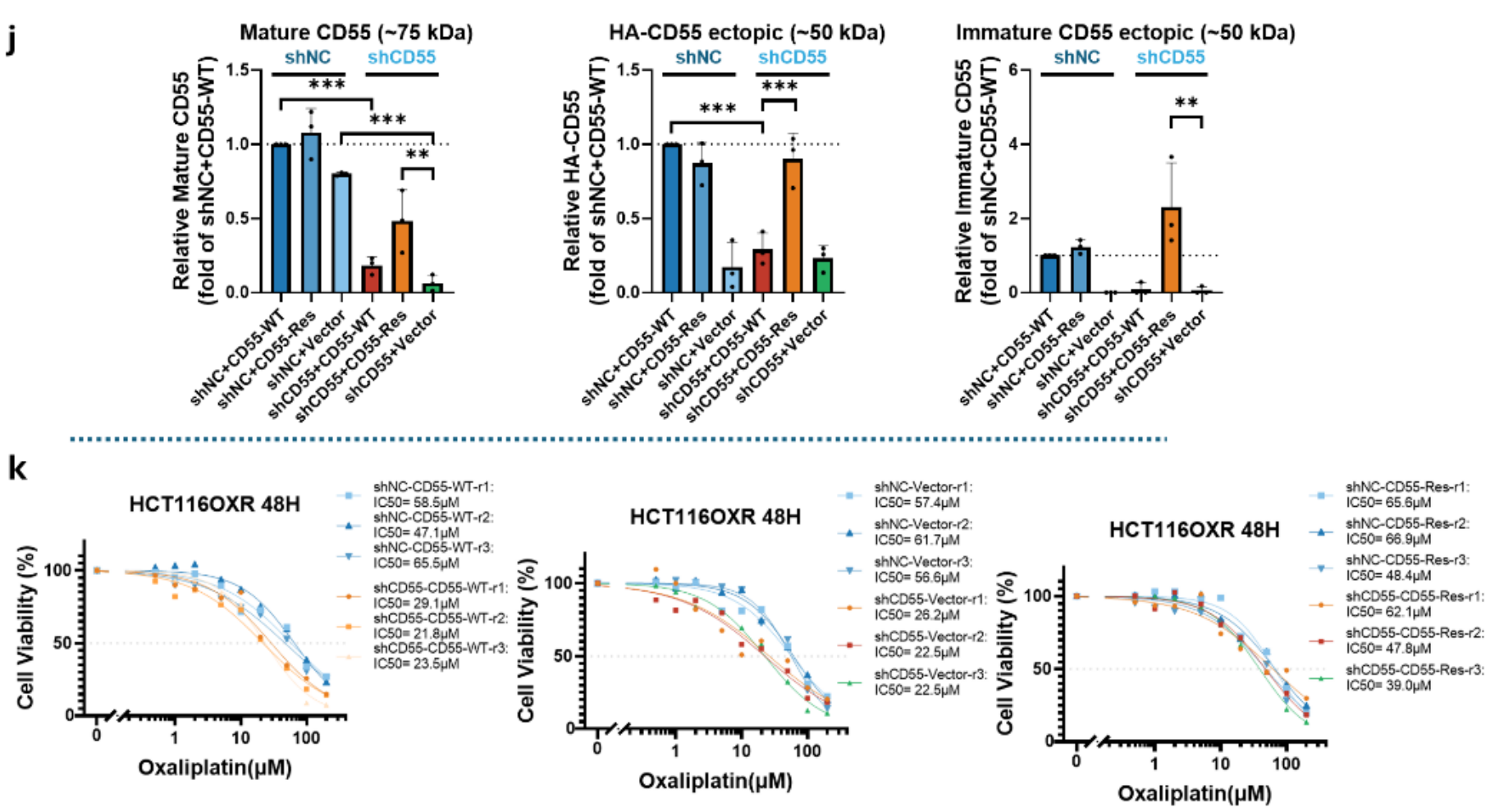

(a) Validation of siCD55 knockdown in HCT116 and HT29 cells: qRT-PCR of CD55 mRNA (left two panels) and densitometric quantification of the CD55 Western blots in Fig. 2a (right two panels), confirming >80% mRNA reduction and reduced protein to 18.9% (HCT116) and 30.7% (HT29) of control. **(b)** Characterization of stable shCD55 lentiviral

knockdown lines. Western blot (top) and qRT-PCR (bottom) confirmed 60–74% reduction of CD55 protein across HCT116, HT29, and HCT116-OXR cells. **(c)** Oxaliplatin dose–response curves (CCK-8, 48 h) for resistant HCT116-OXR and HT29-OXR cells following CD55 knockdown with two independent siRNAs, with individual biological replicates overlaid. Corresponding absolute IC_{50} values are summarized in Fig. 2b. **(d)** Absolute IC_{50} of oxaliplatin in parental HCT116 and HT29 cells following CD55 knockdown with two independent siRNAs, with individual biological replicates overlaid. HCT116: siNC 7.3 ± 3.0 μM vs. siCD55-2 1.8 ± 1.0 μM (≈4.0-fold, $P < 0.05$) and siCD55-4 1.7 ± 1.3 μM (≈4.3-fold, $P = 0.060$, ns); HT29: siNC 29.5 ± 5.2 μM vs. siCD55-2 20.7 ± 4.9 μM (≈1.4-fold, $P = 0.109$, ns) and siCD55-4 17.2 ± 3.5 μM (≈1.7-fold, $P < 0.05$). Per-replicate fit parameters are provided in Supplementary Table S2. **(e)** Oxaliplatin dose–response curves (CCK-8, 48 h) for SW480, LOVO, and RKO cells following CD55 knockdown, with individual biological replicates overlaid. Corresponding absolute IC_{50} values are summarized in Fig. 2c. **(f)** Oxaliplatin dose–response curve (CCK-8, 48 h) for HCT116 cells overexpressing CD55 (CD55-OE) versus empty vector, with individual biological replicates overlaid. Corresponding IC_{50} values are summarized in Fig. 2f. **(g)** Flow-cytometric analysis of apoptosis (Annexin V/PI) after 4 μM oxaliplatin (48 h). CD55 knockdown increased total apoptosis from 33.4% to 47.3% (HCT116) and from 17.4% to 26.6% (HCT116-OXR); late apoptosis was particularly elevated in resistant cells (19.7% vs. 9.2%, $P < 0.001$). **(h)** Western blot of apoptosis-related proteins. CD55 knockdown decreased BCL-2 and increased BAX, reducing the BCL-2/BAX ratio ($P < 0.01$). **(i)** Transwell invasion assay. CD55 knockdown reduced invasion by 50.5% in HCT116 and 73.3% in HCT116-OXR cells. Scale bar, 100 μm. **(j)** Supporting data for the rescue Western blot (Fig. 2h): the anti-HA blot showing the non-specific ~75 kDa band that necessitated restricting HA-CD55 quantification to the ~50 kDa precursor, with replicate densitometry of the ~50 kDa ectopic precursor, which accumulated selectively in shCD55 + CD55-Res.

(k) Per-replicate oxaliplatin dose–response curves (CCK-8, 48 h) for the six rescue conditions, with individual biological replicates overlaid. Corresponding IC_{50} values are summarized in Fig. 2j. Data: mean ± SEM, n = 3 independent biological replicates. Knockdown efficiency (a) by one-way ANOVA with Dunnett's test (each siRNA vs siNC); IC_{50} comparisons (c–f) on $\log_{10}(IC_{50})$ by Welch's unpaired two-tailed t-test; rescue panels (j, k) by one-way ANOVA with Tukey's HSD (k on the $\log_{10}(IC_{50})$ scale); other two-group comparisons (b, g–i) by two-tailed Student's t-test. *P < 0.05, **P < 0.01, ***P < 0.001; ns, not significant.

Figure S3. Supplementary clinical data analysis validates CD55's prognostic value.

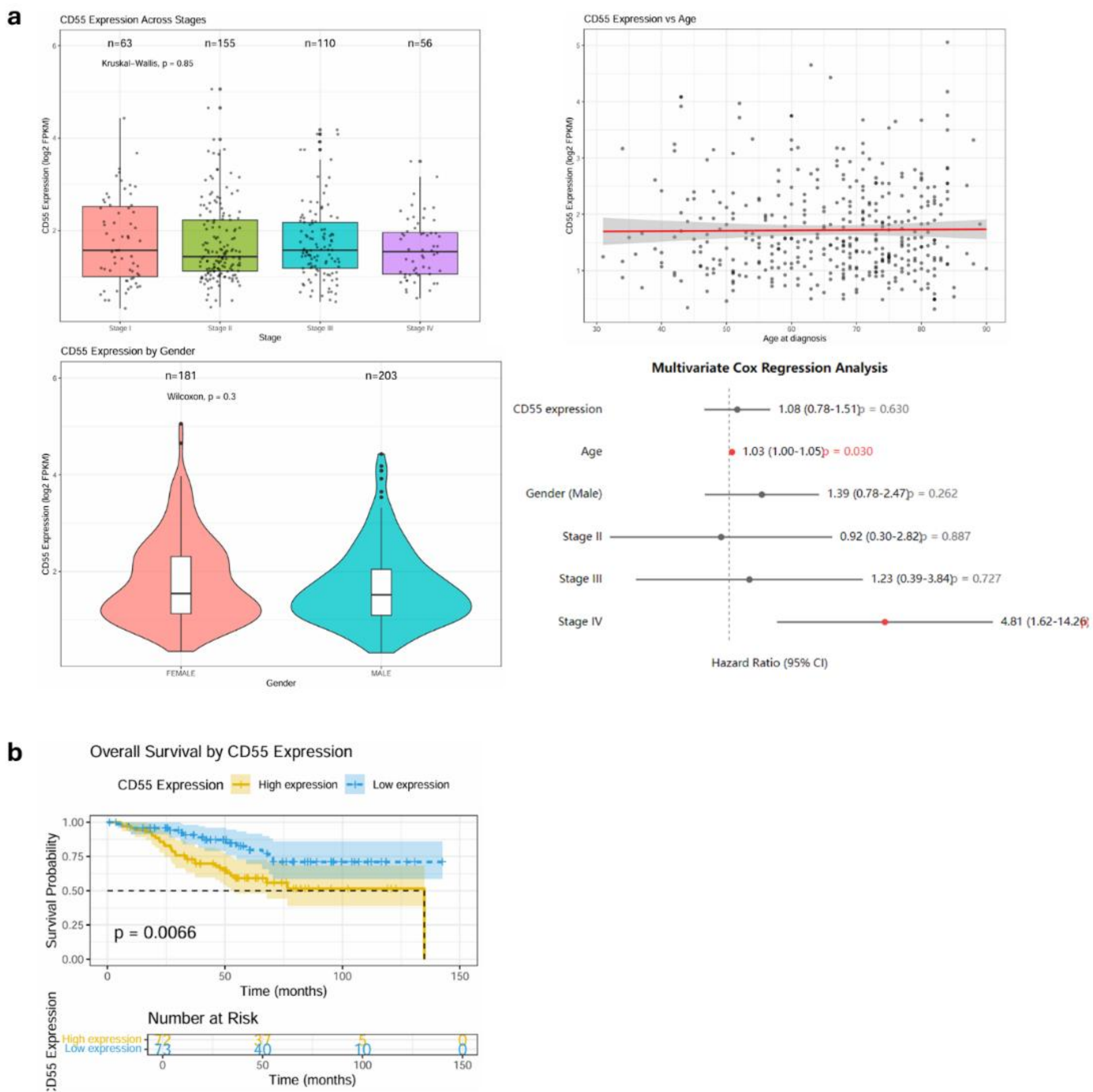

(a) TCGA-COAD database comprehensive analysis of CD55 expression patterns and clinical correlations. Top panels show CD55 expression across different tumor stages (Stage I n=63, Stage II n=155, Stage III n=110, Stage IV n=56) with no significant stage-dependent variation (Kruskal-Wallis test, $P = 0.85$), and scatter plot analysis revealing no significant correlation between CD55 expression and patient age at diagnosis (Spearman correlation, $P > 0.05$). Bottom left: Violin plot comparing CD55 expression between female (n=181) and male (n=203) patients shows no significant gender-based difference (Some samples lack the corresponding information, Wilcoxon test, $P = 0.3$). Bottom right: Forest plot of multivariate Cox regression analysis for overall survival showing hazard ratios with 95% confidence intervals. CD55 expression shows a non-significant trend toward increased risk (HR = 1.08, 95% CI: 1.08-1.51...

levels. High CD55 expression group (orange line, n = 72) shows significantly shorter overall survival compared to low expression group (blue line, n = 73) (P < 0.01, log-rank test). Number at risk table displays patient counts remaining at different time points (0, 50, 100, 150 months).

Figure S4. Validation of the DR-GFP reporter and supporting analyses.

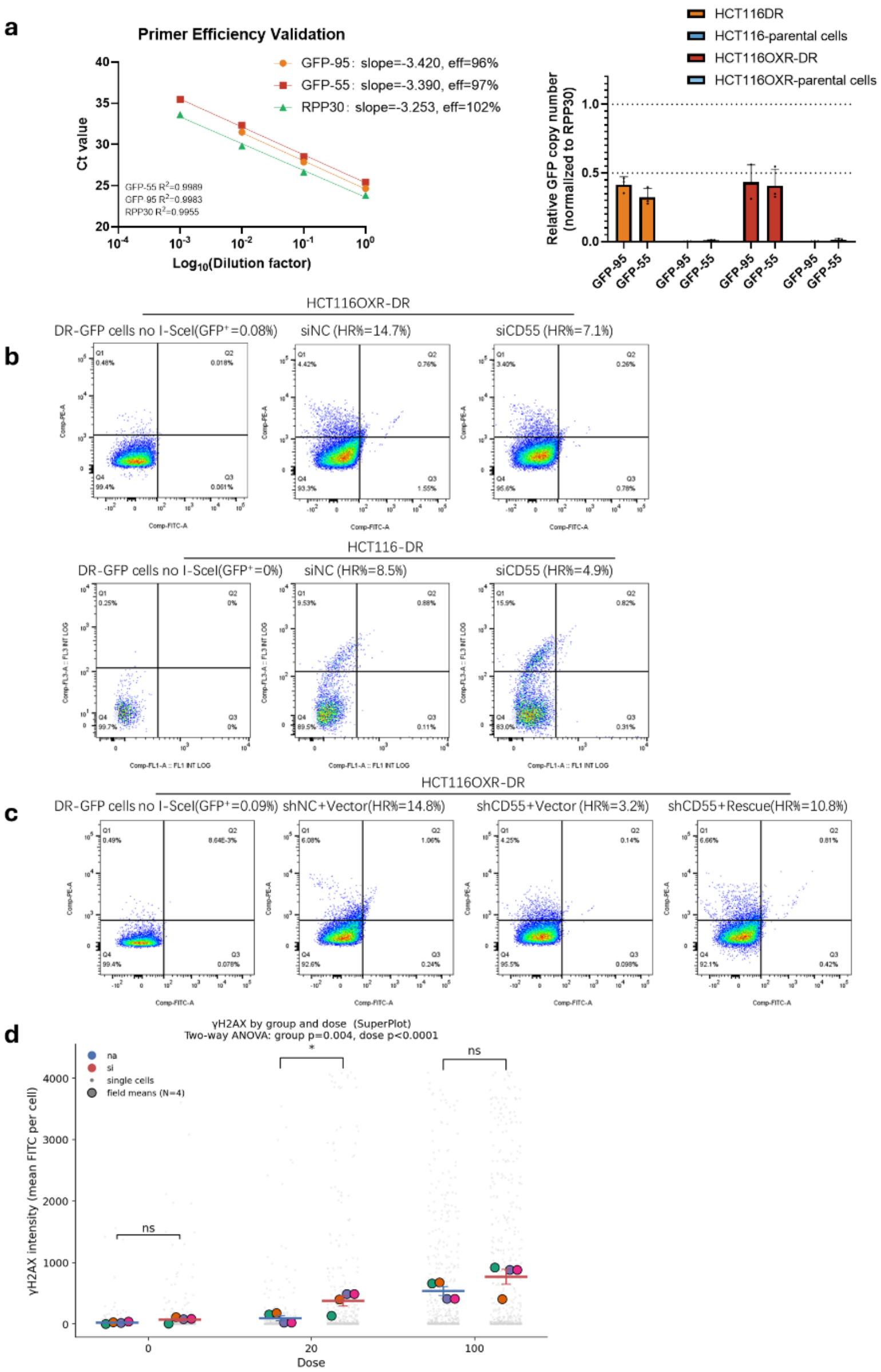

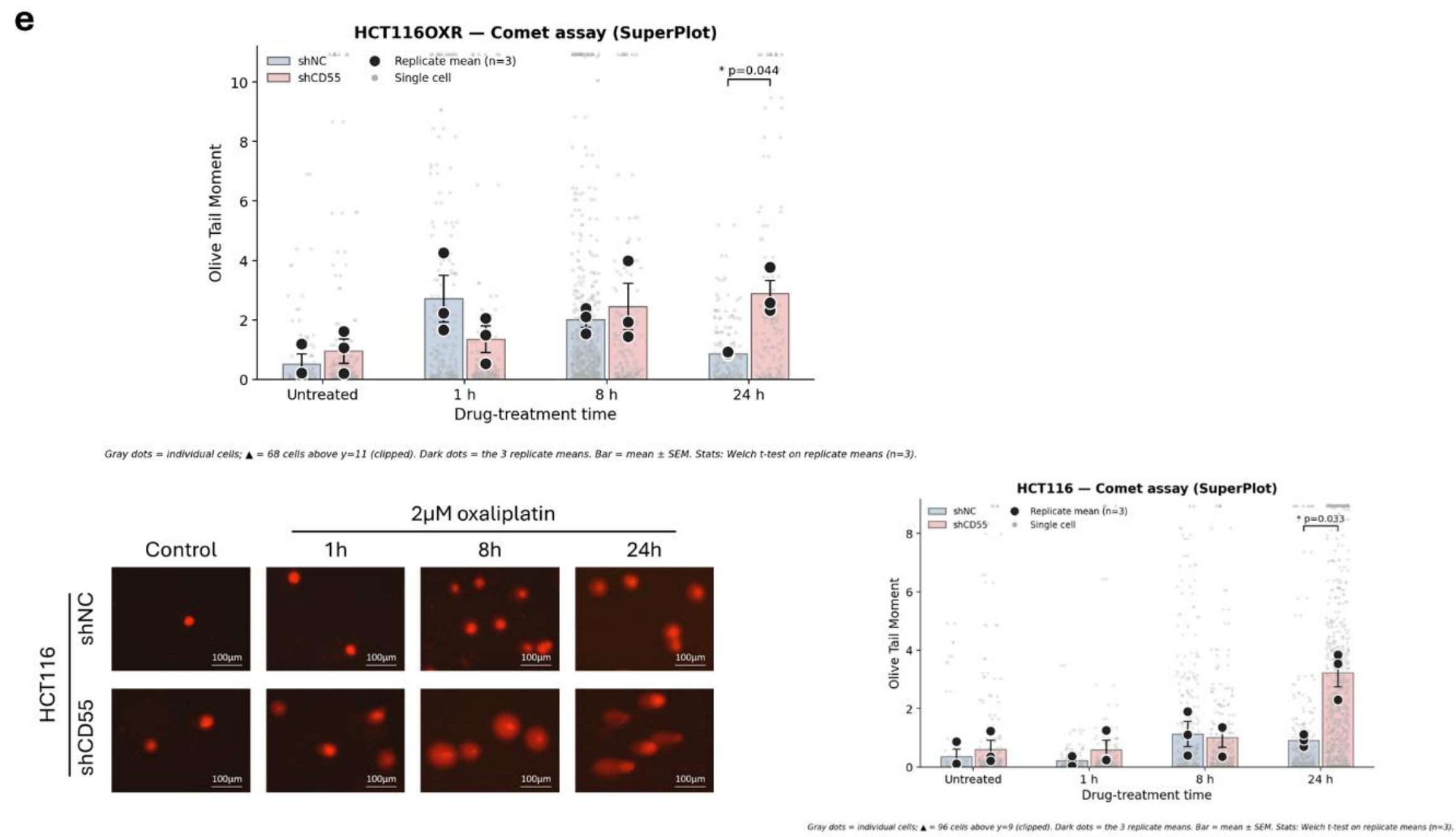

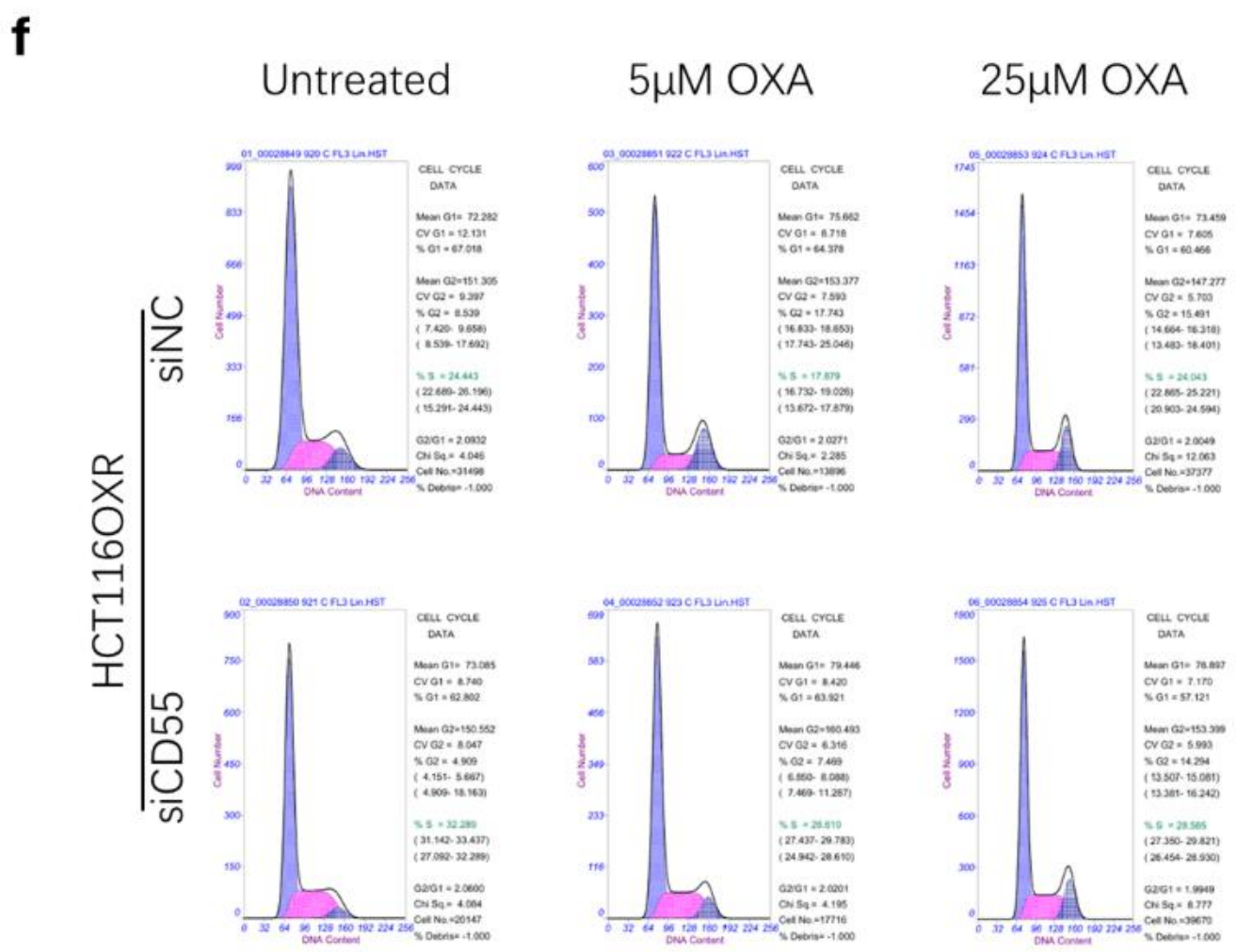

(a) Reporter copy-number verification. Left: standard curves and amplification efficiencies for two independent GFP primer sets (GFP-95: 96%, slope −3.420; GFP-55: 97%, slope −3.390) and the single-copy reference gene RPP30 (102%, slope −3.253). Right: RPP30-normalized relative DR-GFP copy number (ΔΔCt) for the selected HCT116-DR and HCT116-OXR-DR clones, determined with both GFP primer sets; values are consistent with single-copy integration. Parental HCT116 and HCT116-OXR cells (lacking the reporter) yielded essentially background signal, confirming primer specificity. Data: mean ± SEM,, n = 3. **(b)** Representative flow-cytometry dot plots for the knockdown experiment in Fig. 4b: HCT116-DR and HCT116-OXR-DR cells transfected with siNC or siCD55, together with no-I-SceI controls. The gating hierarchy was debris exclusion (FSC-A/SSC-A) → singlet exclusion (FSC-H/FSC-A) → RFP^+ gate → GFP^+ gate,

with thresholds defined on no-plasmid controls; HR efficiency = %GFP^+ within RFP^+ [Q2/(Q2 + Q1)]. **(c)** Representative flow-cytometry dot plots for the rescue experiment in Fig. 4c: HCT116-OXR-DR cells expressing shNC + Vector, shCD55 + Vector, or shCD55 + shRNA-resistant CD55 (rescue), with no-I-SceI controls. **(d)** Per-cell SuperPlot of nuclear γ-H2AX intensity for the experiment in Fig. 4d (small points, individual cells colour-coded by biological replicate; large points, replicate means; bars, mean ± SEM). **(e)** Per-cell comet SuperPlot for HCT116-OXR (Fig. 4e), and representative comet images plus the corresponding SuperPlot for parental HCT116 cells (10 µM oxaliplatin, 1 h; recovery 0–24 h). CD55-depleted HCT116 cells likewise retained elevated OTM at 24 h (3.23 vs 0.91, ~3.5-fold). Scale bar: 100 µm. **(f)** Full cell-cycle distribution (G1/S/G2-M) and representative DNA-content histograms for the experiment in Fig. 4f. Data are mean ± SEM (N = 4 for d; n = 3 for all others); tests as in the corresponding main-figure panels. $^*P < 0.05$, $^{**}P < 0.01$; ns, not significant.

Figure S5. qRT-PCR analysis of oxaliplatin-induced CD55 mRNA expression.

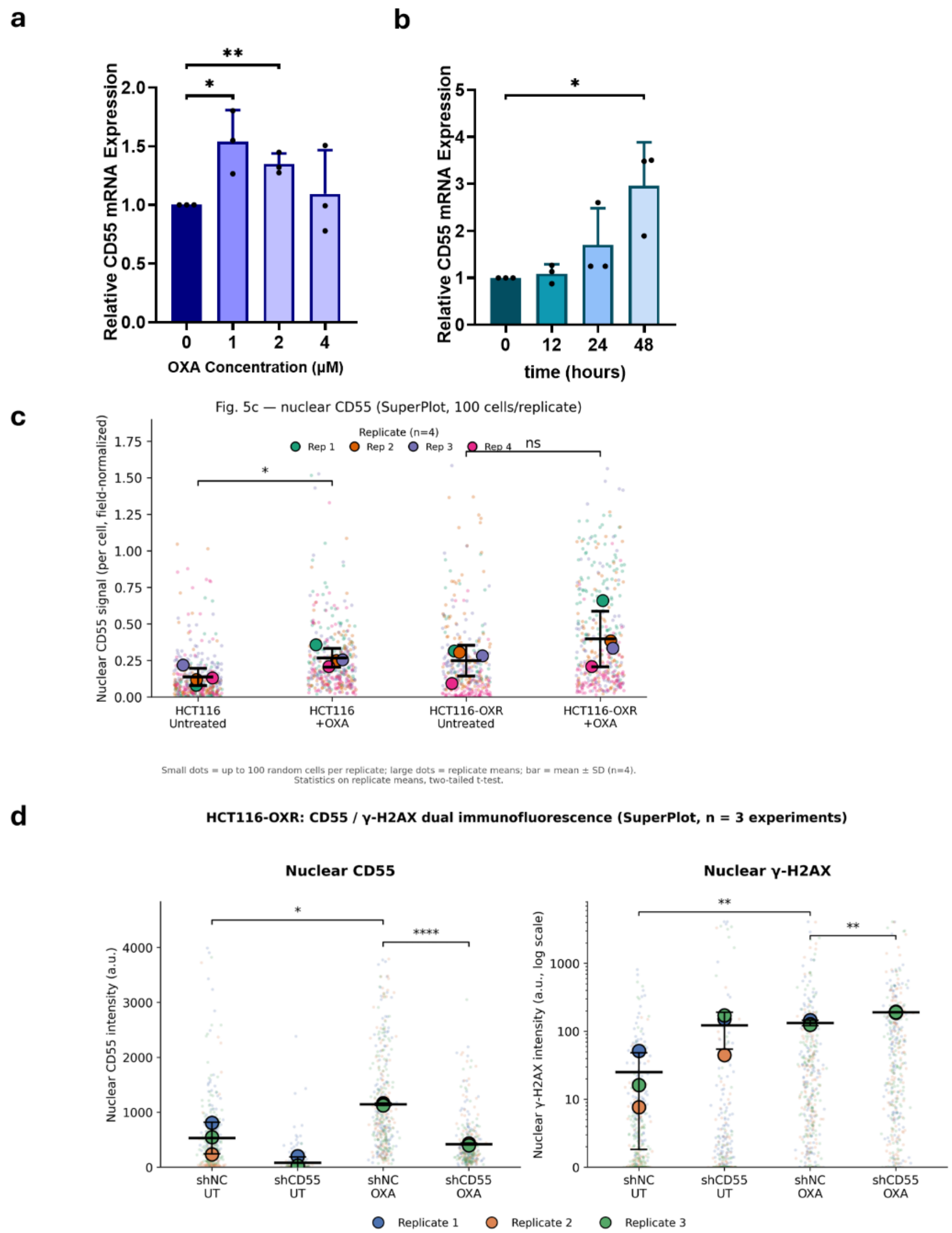

(a) Dose response of CD55 mRNA in HCT116-OXR cells (oxaliplatin 0–4 μM, 48 h): a biphasic profile, rising ~1.5-fold at 1 μM and returning toward baseline by the cytotoxic 4 μM dose. **(b)** Time course of CD55 mRNA (oxaliplatin 2 μM; 0, 12, 24, 48 h), increasing progressively to a maximum of ~2.9-fold at 48 h. **(c)** SuperPlot quantification of nuclear CD55 underlying the representative images in Figure 5c, in HCT116 and HCT116-OXR cells ± oxaliplatin (4 μM). Approximately 40% of HCT116-OXR cells were nuclear CD55–positive after treatment. Small dots, individual cells (⩾ 100 per condition); large dots, per-replicate means; bar, mean ± SEM. **(d)** SuperPlot quantification of nuclear CD55 intensity underlying Figure 5d, in shNC versus shCD55 HCT116-OXR cells ± oxaliplatin (10 μM). Nuclear CD55 was

reduced by 74.7% upon CD55 knockdown. Small dots, individual cells; large dots, per-replicate means; bar, mean ± SEM. Data are mean ± SEM; n = 3 independent experiments. For (a, b), log_2-transformed expression values were analyzed by repeated-measures one-way ANOVA with Geisser–Greenhouse correction and a test for linear trend (time, P = 0.029; dose showed no linear trend, P = 0.83), followed by Dunnett's test versus control. For (c, d), comparisons were performed on the per-replicate means by two-tailed Student's t-test(c, n = 4; d, n = 3). *P < 0.05, **P < 0.01, ***P < 0.001, ****P < 0.0001; ns, not significant.

Figure S6. CD55 acts downstream of ATM activation to support coordinated DDR signaling and RAD51 loading.

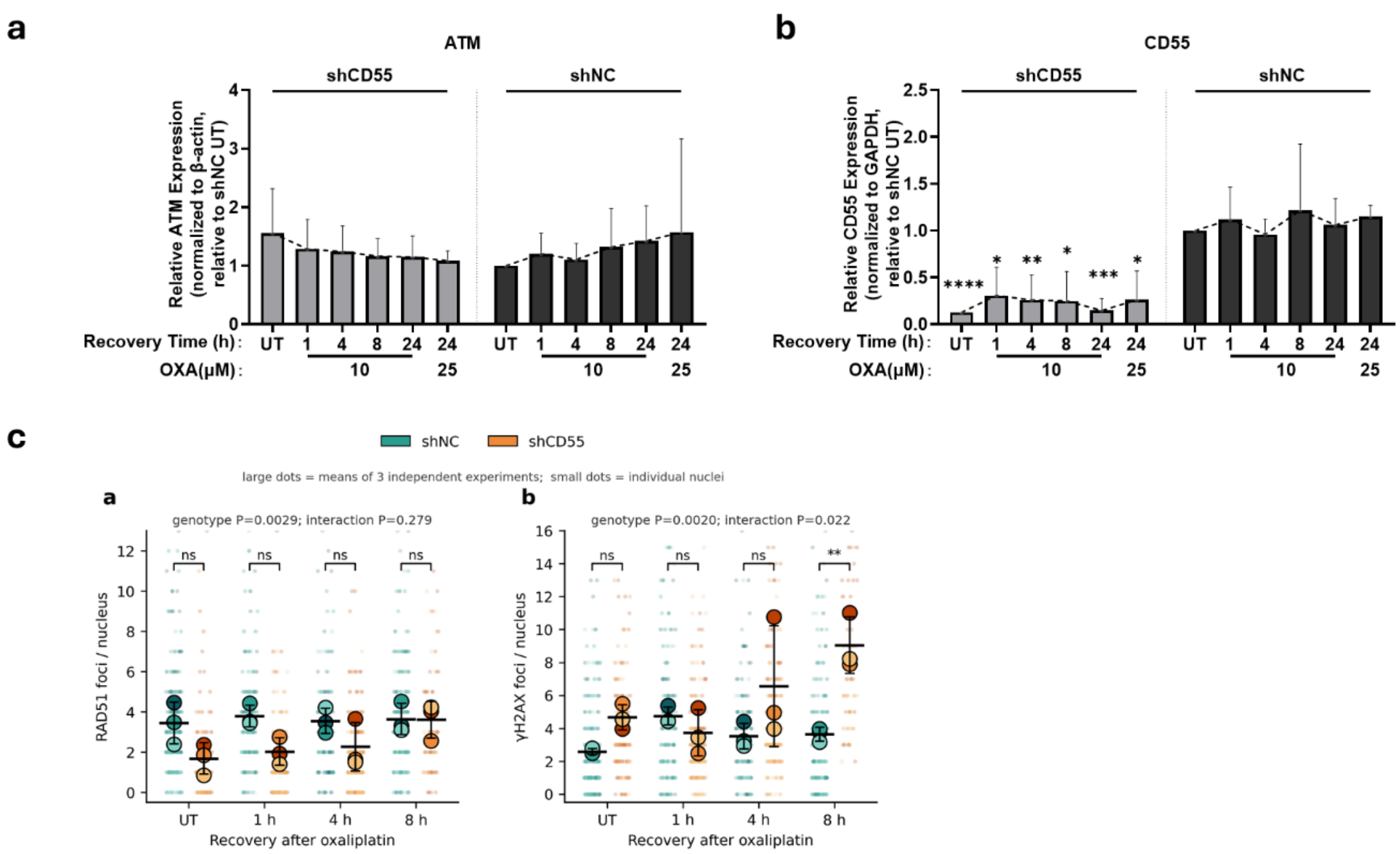

(a) Total ATM (relative to β-actin, normalized to shNC-UT) across the Figure 6a recovery series; total ATM was unchanged between genotypes (ns), confirming the p-ATM/ATM divergence reflects phosphorylation rather than ATM abundance. **(b)** Quantification of CD55 protein from the Figure 6b blots (relative to α-tubulin), confirming sustained CD55 knockdown in shCD55 versus shNC cells across the recovery time course. n = 3; mean ± SEM. **(c)** Superplots of RAD51 and γ-H2AX foci per nucleus in HCT116-OXR shNC and shCD55 cells, untreated and after 10 μM oxaliplatin (1, 4, 8 h recovery), corresponding to the quantification in Figure 6c. Large dots, means of three independent experiments; small dots, individual nuclei. Data are mean ± SEM; n = 3 independent experiments. Two-way ANOVA on experiment-level means with Šídák's test. *P < 0.05, **P < 0.01, ***P < 0.001, ****P < 0.0001; ns, not significant.

Figure S7. Supporting CI analyses and parental-cell controls.

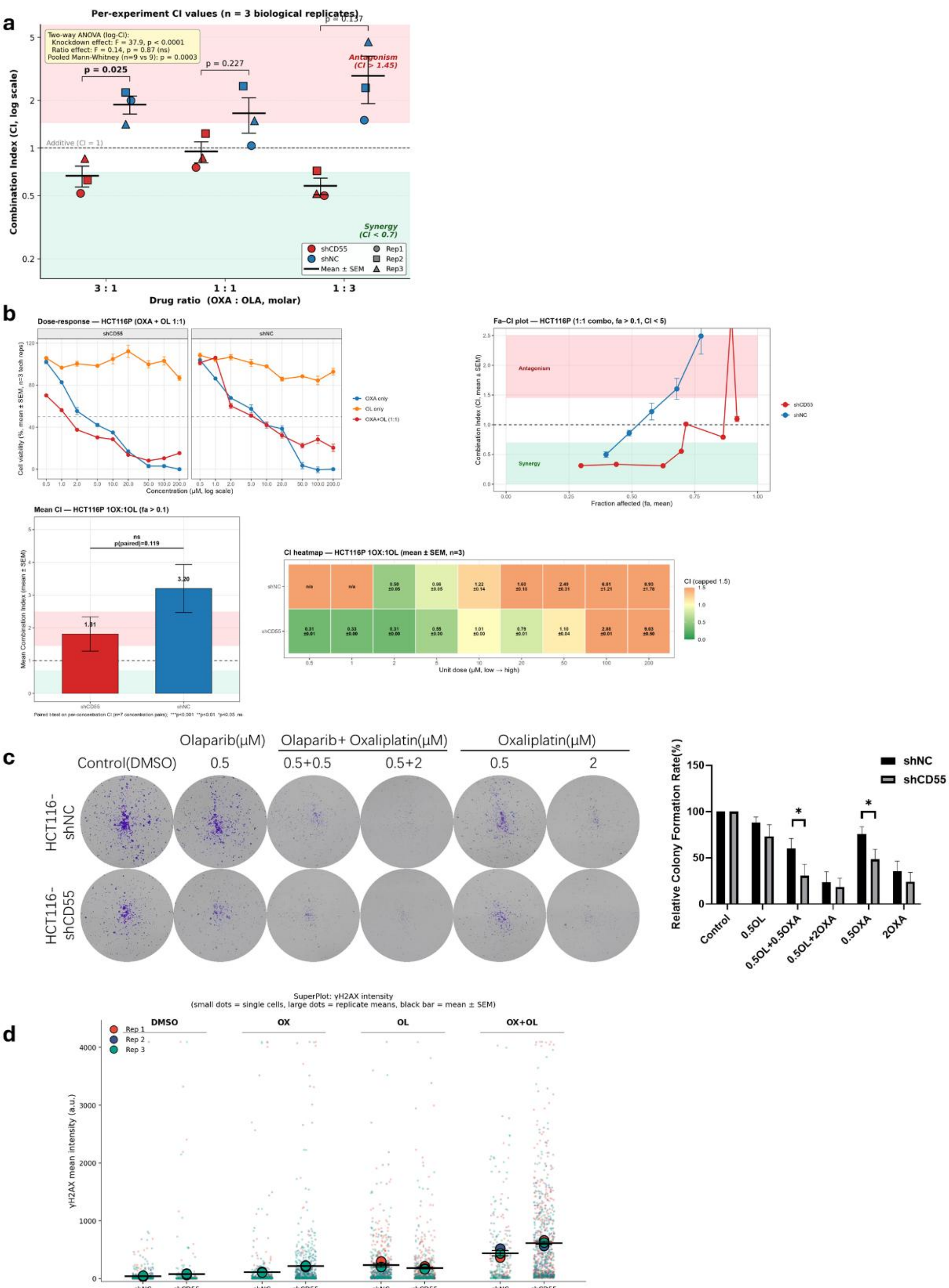

(a) Per-experiment CI values (individual biological replicates, N = 3) in HCT116-OXR shNC vs shCD55 across the three molar ratios, underlying the means in Fig. 7c. **(b)** Parental HCT116: dose–response (OXA, OLA, OXA+OLA 1:1), CI–Fa

plot, mean CI, and per-dose CI heatmap for shNC vs shCD55. The 1:1 combination remained antagonistic regardless of CD55 status, confining the antagonism-to-synergy conversion to the resistant background. **(c)** Long-term clonogenic survival in parental HCT116 cells treated with OLA, OXA, or their combinations; representative colonies and quantification. **(d)** Representative γ-H2AX confocal images for the four-arm experiment quantified in Fig. 7f. Data are mean ± SEM, N = 3; CI interpreted only at Fa > 0.1. Statistics as in Fig. 7. *P < 0.05, **P < 0.01; ns, not significant.

Figure S8. Linear mixed-effects model diagnostics, individual tumor trajectories, and immunohistochemical validation in vivo.

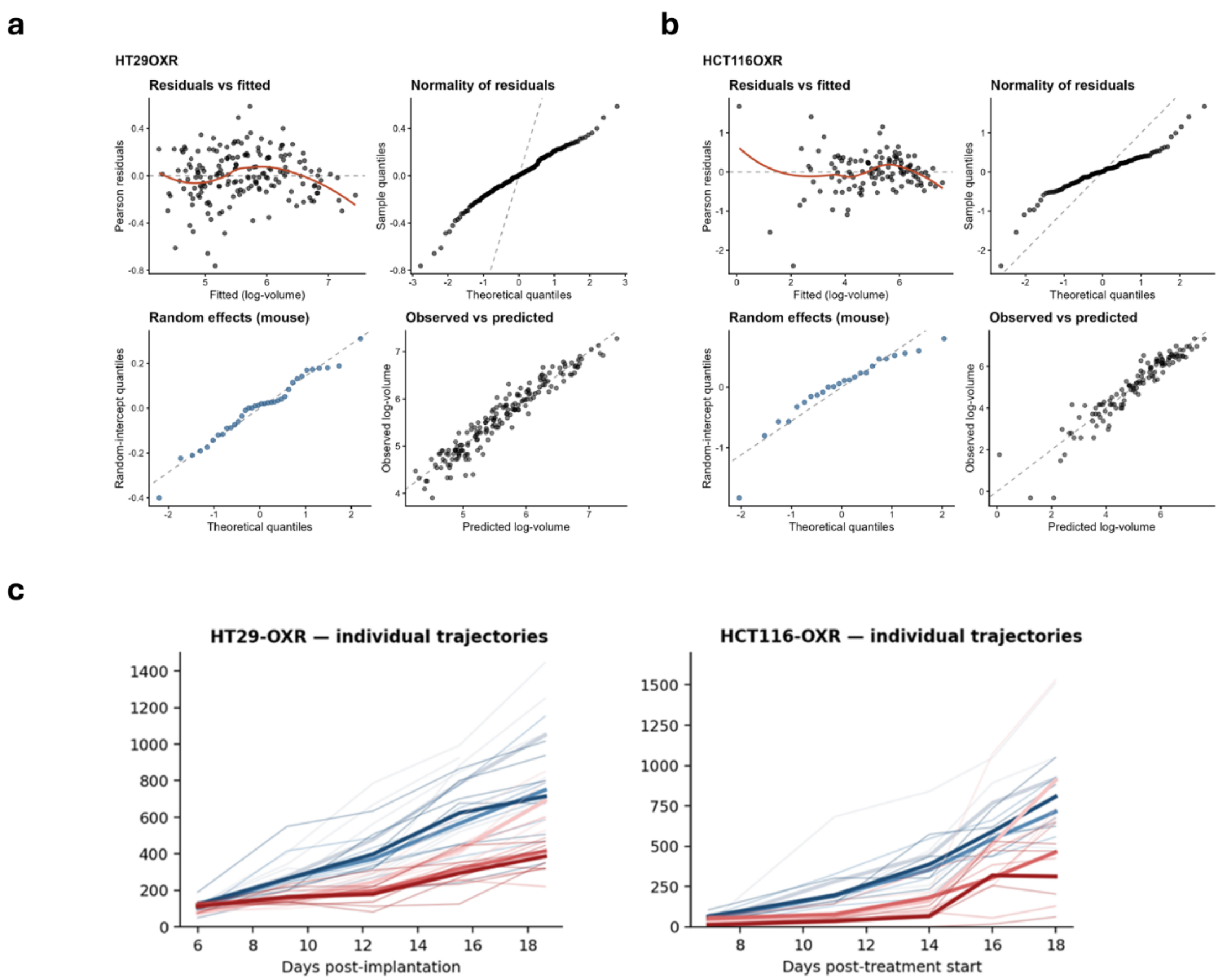

d

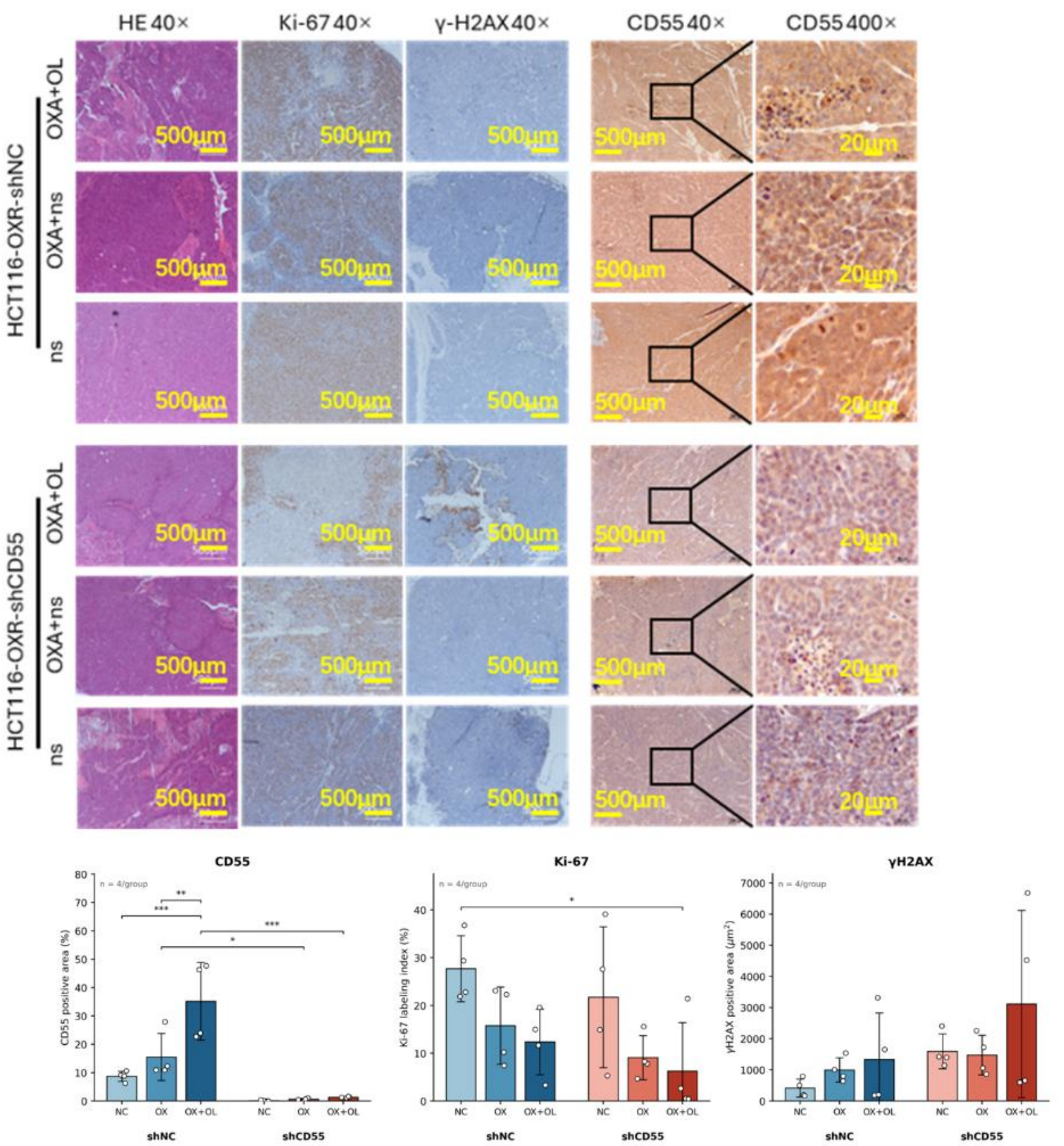

e

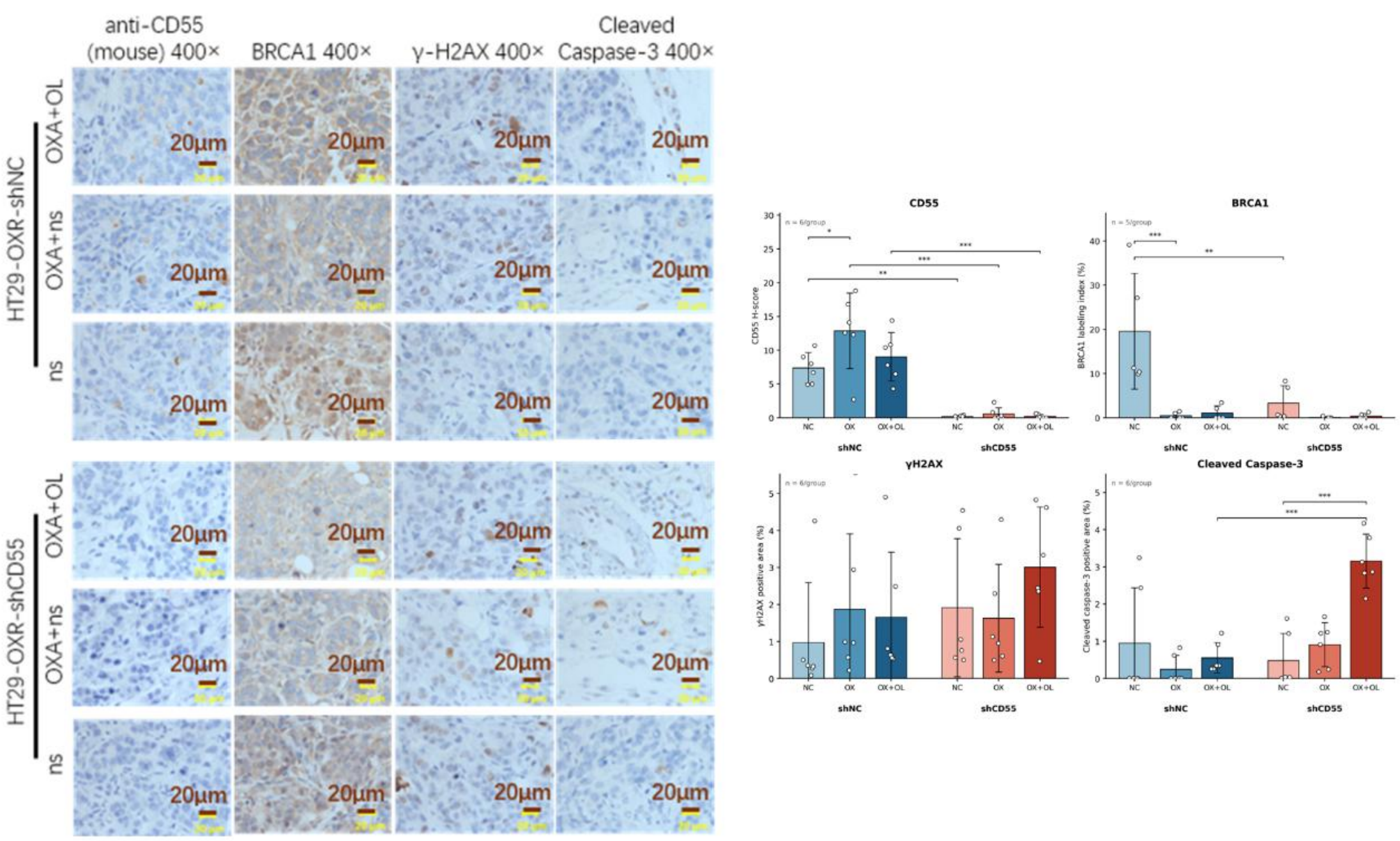

(a, b) Diagnostic plots for the linear mixed-effects model of tumor growth (log-volume) in HT29-OXR (a) and HCT116-OXR (b): Pearson residuals versus fitted values, normal quantile–quantile plot of residuals, quantile–quantile plot of the mouse-level random intercepts, and observed versus predicted log-volume. **(c)** Individual tumor-growth trajectories for

every animal in HT29-OXR (left) and HCT116-OXR (right). Thin lines denote single mice; bold lines denote group means (blue, shCD55; red, shNC). **(d)** Representative HE and immunohistochemical staining (Ki-67, γ-H2AX at 40×; CD55 at 40× and 400×) of HCT116-OXR-shNC and HCT116-OXR-shCD55 tumors across treatment arms (OXA+OL, OXA+ns, ns), with quantification of CD55-positive area, Ki-67 labeling index, and γ-H2AX-positive area below (n = 4/group). **(e)** Representative immunohistochemistry (anti-CD55, BRCA1, γ-H2AX, cleaved caspase-3 at 400×) of HT29-OXR-shNC and HT29-OXR-shCD55 tumors across treatment arms, with quantification of CD55 H-score, BRCA1 labeling index, γ-H2AX-positive area, and cleaved caspase-3-positive area below (n = 5–6/group). Statistics: CD55 knockdown was verified with two independent anti-CD55 antibodies. IHC quantification was compared by two-way ANOVA with Tukey's post hoc test. Data are mean ± SD. *P < 0.05, **P < 0.01, ***P < 0.001; ns, not significant.
